# The genetic architecture of human brainstem structures and their involvement in common brain disorders

**DOI:** 10.1038/s41467-020-17376-1

**Published:** 2020-08-11

**Authors:** Torbjørn Elvsåshagen, Shahram Bahrami, Dennis van der Meer, Ingrid Agartz, Dag Alnæs, Deanna M. Barch, Ramona Baur-Streubel, Alessandro Bertolino, Mona K. Beyer, Giuseppe Blasi, Stefan Borgwardt, Birgitte Boye, Jan Buitelaar, Erlend Bøen, Elisabeth Gulowsen Celius, Simon Cervenka, Annette Conzelmann, David Coynel, Pasquale Di Carlo, Srdjan Djurovic, Sarah Eisenacher, Thomas Espeseth, Helena Fatouros-Bergman, Lena Flyckt, Barbara Franke, Oleksandr Frei, Barbara Gelao, Hanne Flinstad Harbo, Catharina A. Hartman, Asta Håberg, Dirk Heslenfeld, Pieter J. Hoekstra, Einar A. Høgestøl, Rune Jonassen, Erik G. Jönsson, Karolinska Schizophrenia Project (KaSP) consortium, L. Farde, L. Flyckt, G. Engberg, S. Erhardt S, H. Fatouros-Bergman, S. Cervenka, L. Schwieler, F. Piehl, I. Agartz, K. Collste, P. Victorsson, A. Malmqvist, M. Hedberg, F. Orhan, C. M. Sellgren, Peter Kirsch, Iwona Kłoszewska, Trine Vik Lagerberg, Nils Inge Landrø, Stephanie Le Hellard, Klaus-Peter Lesch, Luigi A. Maglanoc, Ulrik F. Malt, Patrizia Mecocci, Ingrid Melle, Andreas Meyer-Lindenberg, Torgeir Moberget, Jan Egil Nordvik, Lars Nyberg, Kevin S. O’ Connell, Jaap Oosterlaan, Marco Papalino, Andreas Papassotiropoulos, Paul Pauli, Giulio Pergola, Karin Persson, Dominique de Quervain, Andreas Reif, Jaroslav Rokicki, Daan van Rooij, Alexey A. Shadrin, André Schmidt, Emanuel Schwarz, Geir Selbæk, Hilkka Soininen, Piotr Sowa, Vidar M. Steen, Magda Tsolaki, Bruno Vellas, Lei Wang, Eric Westman, Georg C. Ziegler, Mathias Zink, Ole A. Andreassen, Lars T. Westlye, Tobias Kaufmann

**Affiliations:** 1 NORMENT, Division of Mental Health and Addiction, Oslo University Hospital, Oslo, Norway; 2 Department of Neurology, Division of Clinical Neuroscience, Oslo University Hospital, Oslo, Norway; 3 Institute of Clinical Medicine, University of Oslo, Oslo, Norway; 4 School of Mental Health and Neuroscience, Faculty of Health, Medicine and Life Sciences, Maastricht University, Maastricht, The Netherlands; 5 Department of Psychiatric Research, Diakonhjemmet Hospital, Oslo, Norway; 6 Centre for Psychiatry Research, Department of Clinical Neuroscience, Karolinska Institutet & Stockholm Health Care Services, Stockholm Region, Stockholm, Sweden; 7 NORMENT, Institute of Clinical Medicine, University of Oslo, Oslo, Norway; 8 Departments of Psychological & Brain Sciences, Psychiatry, and Radiology, Washington University in St. Louis, St. Louis, USA; 9 Department of Psychology I and Centre of Mental Health, University of Würzburg, Würzburg, Germany; 10 Institute of Psychiatry, University of Bari, Bari, Italy; 11 Department of Basic Medical Science, Neuroscience, and Sense Organs, University of Bari Aldo Moro, Bari, Italy; 12 Division of Radiology and Nuclear Medicine, Oslo University Hospital, Oslo, Norway; 13 Department of Psychiatry (UPK), University of Basel, Basel, Switzerland; 14 Institute of Psychiatry, King’s College, London, UK; 15 Department of Psychiatry and Psychotherapy, University of Lübeck, Lübeck, Germany; 16 Psychosomatic and CL Psychiatry, Division of Mental Health and Addiction, Oslo University Hospital, Oslo, Norway; 17 Department of Behavioural Sciences in Medicine, University of Oslo, Oslo, Norway; 18 Department of Cognitive Neuroscience, Donders Institute for Brain, Cognition and Behaviour, Radboud University Medical Center, Nijmegen, The Netherlands; 19 Karakter Child and Adolescent Psychiatry University Centre, Nijmegen, The Netherlands; 20 Children and Adolescence Psychiatry, University of Tübingen, Tübingen, Germany; 21 Transfaculty Research Platform Molecular and Cognitive Neurosciences, University of Basel, CH-4055 Basel, Switzerland; 22 Division of Cognitive Neuroscience, Department of Psychology, University of Basel, CH-4055 Basel, Switzerland; 23 Department of Medical Genetics, Oslo University Hospital, Oslo, Norway; 24 NORMENT, Department of Clinical Science, University of Bergen, Bergen, Norway; 25 Department of Psychiatry and Psychotherapy, Central Institute of Mental Health, Medical Faculty Mannheim, Heidelberg University, Mannheim, Germany; 26 Institute of Psychiatric and Psychosomatic Psychotherapy, Central Institute of Mental Health, Medical Faculty Mannheim, Heidelberg University, Mannheim, Germany; 27 Department of Psychology, University of Oslo, Oslo, Norway; 28 Bjørknes College, Lovisenberggata, 13, 0456 Oslo, Norway; 29 Departments of Human Genetics and Psychiatry, Donders Institute for Brain, Cognition and Behaviour, Radboud University Medical Center, Nijmegen, The Netherlands; 30 Department of Psychiatry, University of Groningen, University Medical Center Groningen, Groningen, The Netherlands; 31 Department of Neuromedicine and Movement Science, Norwegian University of Science and Technology, Trondheim, Norway; 32 Department of Radiology and Nuclear Medicine, St. Olavs Hospital, Trondheim, Norway; 33 Clinical Neuropsychology section, Vrije Universiteit Amsterdam, Amsterdam, The Netherlands; 34 Department of Cognitive Psychology, Vrije Universiteit Amsterdam, Amsterdam, The Netherlands; 35 Department of Child and Adolescent Psychiatry, University Medical Center Groningen, University of Groningen, Groningen, The Netherlands; 36 Faculty of Health Sciences, Oslo Metropolitan University, Oslo, Norway; 37 Department of Clinical Psychology, Central Institute of Mental Health, Medical Faculty Mannheim, Heidelberg University, Mannheim, Germany; 38 Bernstein Center for Computational Neuroscience Heidelberg/Mannheim, Mannheim, Germany; 39 Department of Old Age Psychiatry and Psychotic Disorders, Medical University of Lodz, Lodz, Poland; 40 Division of Molecular Psychiatry, Center of Mental Health, University of Würzburg, Würzburg, Germany; 41 Laboratory of Psychiatric Neurobiology, Institute of Molecular Medicine, Sechenov First Moscow State Medical University, Moscow, Russia; 42 Department of Psychiatry and Neuropsychology, School for Mental Health and Neuroscience (MHeNS), Maastricht University, Maastricht, The Netherlands; 43 Institute of Gerontology and Geriatrics, University of Perugia, Perugia, Italy; 44 The CatoSenteret Rehabilitation Center, Son, Norway; 45 Departments of Radiation Sciences and Integrative Medical Biology, Umeå Center for Functional Brain Imaging, Umeå University, Umeå, Sweden; 46 Emma Children’s Hospital Amsterdam UMC, University of Amsterdam, Emma Neuroscience Group, Department of Pediatrics, Amsterdam Reproduction & Development, Amsterdam, The Netherlands; 47 Psychiatric University Clinics, University of Basel, Basel, Switzerland; 48 Department Biozentrum, Life Sciences Training Facility, University of Basel, Basel, Switzerland; 49 Department of Geriatric Medicine, Oslo University Hospital, Oslo, Norway; 50 Norwegian National Advisory Unit on Ageing and Health, Vestfold Hospital Trust, Tønsberg, Norway; 51 Department of Psychiatry, Psychosomatic Medicine and Psychotherapy, University Hospital Frankfurt, Frankfurt am Main, Germany; 52 Department of Neurology, Institute of Clinical Medicine, University of Eastern Finland, Kuopio, Finland; 53 Neurocenter, Neurology, Kuopio University Hospital, Kuopio, Finland; 54 Dr. E. Martens Research Group for Biological Psychiatry, Department of Medical Genetics, Haukeland University Hospital, Bergen, Norway; 55 1st University Department of Neurology, Aristotle University of Thessaloniki, Makedonia, Greece; 56 INSERM U 1027, University of Toulouse, Toulouse, France; 57 Feinberg School of Medicine, Northwestern University, Chicago, IL USA; 58 Department of Neurobiology, Care Sciences and Society, Karolinska Institute, Stockholm, Sweden; 59 District hospital Mittelfranken, Ansbach, Germany; 60 Department of Physiology and Pharmacology, Karolinska Institutet, Stockholm, Sweden; 61 Neuroimmunology Unit, Department of Clinical Neuroscience, Karolinska Institutet, Stockholm, Sweden

**Keywords:** Genome-wide association studies, Diseases of the nervous system, Genetics of the nervous system, Brain

## Abstract

Brainstem regions support vital bodily functions, yet their genetic architectures and involvement in common brain disorders remain understudied. Here, using imaging-genetics data from a discovery sample of 27,034 individuals, we identify 45 brainstem-associated genetic loci, including the first linked to midbrain, pons, and medulla oblongata volumes, and map them to 305 genes. In a replication sample of 7432 participants most of the loci show the same effect direction and are significant at a nominal threshold. We detect genetic overlap between brainstem volumes and eight psychiatric and neurological disorders. In additional clinical data from 5062 individuals with common brain disorders and 11,257 healthy controls, we observe differential volume alterations in schizophrenia, bipolar disorder, multiple sclerosis, mild cognitive impairment, dementia, and Parkinson’s disease, supporting the relevance of brainstem regions and their genetic architectures in common brain disorders.

## Abstract (web summary)

The genetic architecture underlying brainstem regions and how this links to common brain disorders is not well understood. Here, the authors use MRI and GWAS data from 27,034 individuals to identify genetic and morphological brainstem features that influence common brain disorders.

## Introduction

The brainstem is a critical regulator of vital bodily functions and includes the midbrain, pons, and the medulla oblongata^1,2^. These regions subserve emotions and behavior and are implicated in the pathophysiology of psychiatric and neurological diseases^3–6^. The midbrain is involved in reward-related behavior and associated with addictive, psychotic, and neurodegenerative disorders^7–9^. Midbrain and pons neurons support mood and cognition and may have central roles in the etiology and treatment of affective disorders^10,11^. The medulla oblongata regulates cardiovascular and respiratory function and atrophy and lesions of medulla oblongata and the other brainstem structures are hallmarks of neurological disorders^5,6,8^. Despite their importance in human health and disease, the brainstem regions remain markedly understudied.

Magnetic resonance imaging (MRI) studies have revealed cortical and subcortical structural alterations in psychiatric and neurological disorders^12–14^, and the discovery of genetic contributions to brain structure variation has begun^15,16^. Recent imaging-genetics studies detected the first genetic loci linked to whole brainstem volume^17–19^, yet no large-scale neuroimaging study has focused on the differential genetic architecture of the midbrain, pons, and medulla oblongata and their involvement in common brain disorders. The unprecedented availability of large imaging-genetics resources and recent development of a Bayesian brainstem segmentation algorithm^20^ allow us to estimate volumes of midbrain, pons, medulla oblongata, superior cerebellar peduncle (SCP, which interconnects the pons and the cerebellum), and the whole brainstem in a large sample. We employ three complementary approaches to increase our knowledge of the genetic underpinnings of brainstem regions and their roles in common brain disorders.

First, we conduct genome-wide association studies (GWAS) of brainstem volumes in UK Biobank participants^21^ and identify the first genetic loci linked to the midbrain, pons, SCP, and the medulla oblongata. Second, we use summary statistics from recent large-scale GWAS of common brain disorders and detect genetic overlap between volumes of the brainstem regions and eight psychiatric and neurological disorders. Finally, we examine volumes of the brainstem structures in individuals with psychiatric or neurological illnesses in comparison with healthy controls (HC) (*n* = 16,319) and find volume alterations in schizophrenia (SCZ), bipolar disorder (BD), multiple sclerosis (MS), dementia, mild cognitive impairment (MCI), and Parkinson’s disease (PD). Collectively, these results provide new insights into the genetic architectures of brainstem structures and their roles in common brain disorders.

## Results

### Brainstem segmentation and samples

We obtained raw T1 3D brain MRI data from a total of *n* = 57,298 individuals, collected through collaborations, data sharing platforms, and from in-house samples (Supplementary Tables 1–2). The MRI data were segmented into the whole brainstem, midbrain, pons, SCP, and medulla oblongata using Freesurfer 6.0^22^ and Bayesian brainstem segmentation, robust to differences in MRI scanners and pulse sequence details^20^. We assessed the delineations in all 57,298 data sets by visually inspecting twelve sagittal view figures of the segmentations for each participant (Supplementary Fig. 1). This procedure was conducted blind to case-control status and excluded 11.4% (*n* = 6513) of the data sets, mainly due to insufficient field of view, image quality, and segmentation errors in the clinical samples.

The resulting 50,785 MRI data sets comprised three main samples (Supplementary Table 3): (1) 27,034 genotyped individuals from the UK Biobank (GWAS discovery sample; age 45–82 years); (2) 7432 additional genotyped individuals from the UK Biobank (GWAS replication sample; age 50–82 years), and 3) 16,319 individuals with psychiatric or neurological disorders and HC (clinical sample; age 3–96 years; 5062 patients and 11,257 controls). Only a small minority in the clinical sample had genotype data and thus no genetic analyses were run for these individuals.

### GWAS reveals 61 loci associated with brainstem volumes

Using MRI and single-nucleotide polymorphism (SNP) data from the GWAS discovery sample (*n* = 27,034), we conducted GWAS with PLINK v2.0^23^ on volumes of the midbrain, pons, SCP, medulla oblongata, and whole brainstem. All GWAS accounted for age, age², sex, scanning site, intracranial volume (ICV), genotyping batch, and the first ten genetic principal components to control for population stratification. In addition, the GWAS for the midbrain, pons, SCP, and medulla oblongata accounted for whole brainstem volume, thus revealing genetic signals beyond commonality in volume, analogous to a recent study of hippocampal subfields^24^. In additional supplemental analyses, the GWAS for the brainstem structures were also run without covarying for whole brainstem volume and when excluding individuals related up to 4th degree.

**Fig. 1:**
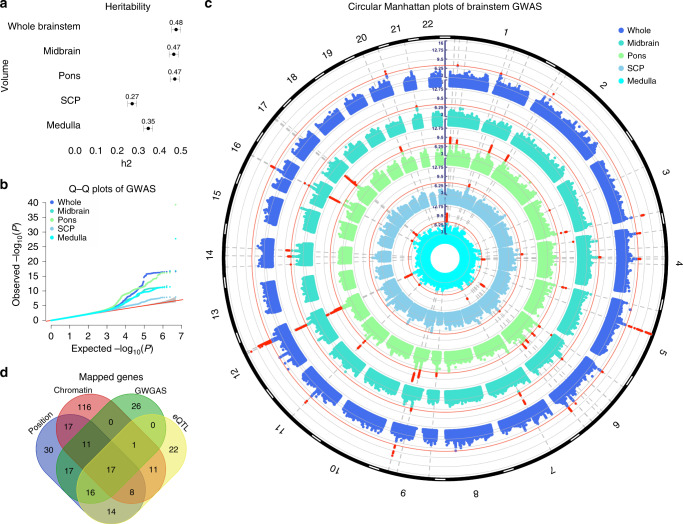
Analysis of GWAS discovery sample identifies 61 loci associated with brainstem volumes. **a** GCTA-based heritability estimates for the brainstem volumes in the discovery sample of *n* = 27,034 participants from the UK Biobank. All brainstem volumes showed substantial heritability, with higher estimates for the whole brainstem (*h*_*2*_ = 0.48), midbrain (*h*_*2*_ = 0.47), and pons (*h*_*2*_ = 0.47) and lower for the medulla oblongata (*h*_*2*_ = 0.35) and SCP (*h*_*2*_ = 0.27). Error bars, s.e. **b** Q−Q plots for the brainstem volumes of the discovery sample. **c** Circular Manhattan plots of GWAS for brainstem volumes of the discovery sample. The outermost plot in blue reflects the GWAS of whole brainstem volume, whereas, from the periphery to center, the turquoise, green, gray/blue, and cyan plots indicate the GWAS of the midbrain, pons, SCP, and medulla oblongata volumes, respectively. Red circular lines indicate genome-wide significance and the red radial lines are significant loci (two-sided *P* < 5e−8). **d** Venn diagram showing number of genes mapped by the four different strategies in the discovery sample, i.e., positional gene (blue), expression quantitative trait loci (eQTL; yellow), and chromatin interaction mapping (red), and identification by the GWGAS (green). Seventeen genes were identified by all four approaches. Whole whole brainstem. SCP superior cerebellar peduncle. Medulla medulla oblongata. GW(G)AS genome-wide (gene-based) association analyses. GCTA genome-wide complex trait analysis.

SNP-based heritability estimated using genome-wide complex trait analysis (GCTA) v1.92^25^ on the GWAS summary statistics was 48% for the whole brainstem, 47% for the midbrain, 47% for pons, 27% for SCP, and 35% for the medulla oblongata (all s.e. <5%; all *P* < 1e−16), illustrating the substantial genetic influence on brainstem volumes (Fig. 1a and Supplementary Table 4). We found genome-wide significant hits (*P* < 5e−8) for all brainstem volumes and identified a total of 125 independent significant SNPs across structures located in 61 genomic loci, using the Functional Mapping and Annotation of GWAS (FUMA) platform v1.3.5^26^ (Fig. 1b–c and Supplementary Data 1). Individual Manhattan and quantile–quantile (Q–Q) plots for each brainstem volume are provided in Supplementary Figs. 2–3. Supplementary Fig. 4 shows regional plots for the most significant genetic locus for each brainstem volume. GWAS hits and heritability estimates for the brainstem regions without covarying for whole brainstem volume are provided in Supplementary Fig. 5, Supplementary Table 4, and Supplementary Data 2. Supplementary Data 3 shows the GWAS results when excluding individuals related up to 4th degree from the analyses (*n* = 705). A large majority of the lead SNPs identified in the main analyses had *P* < 5e−8 also in the sample without any up to 4th degree related individuals (*n* = 26,329), supporting that the GWAS results were not driven by relatedness.

Sixteen of the 61 genetic loci were associated with whole brainstem volume and 10, 23, 3, and 9 loci were associated with volumes of the midbrain, pons, SCP, and medulla oblongata, respectively. Sixteen loci were associated with more than one brainstem volume, thus resulting in 45 unique brainstem-associated genetic regions. Twenty-nine of these unique loci were associated with volumes of the individual brainstem regions and not with whole brainstem volume. Moreover, 6, 15, 3, and 3 loci were only significant for midbrain, pons, SCP, and medulla oblongata volumes, respectively, whereas 10 of the loci were only associated with whole brainstem volume (Supplementary Data 1).

When employing a more stringent statistical threshold of *P* < 1e−8 (corrected for analyses of 5 volumes), there were 46 significant genetic loci across the brainstem structures (Supplementary Data 4). Thirteen loci were associated with whole brainstem volume and 6, 21, and 6 loci were associated with volumes of the midbrain, pons, and medulla oblongata, respectively. No locus was significant for SCP. The 46 genomic loci included 34 unique brainstem-associated regions of which 21 were only associated with volumes of the individual brainstem regions and not with whole brainstem volume.

### Further evidence of association in the replication GWAS

The brainstem-associated lead SNPs of the discovery sample with *P* < 5e−8 were further evaluated in the replication GWAS (*n* = 7432). We found that all SNPs had the same effect direction for most of the volumes (all but a few SNPs for medulla oblongata and midbrain volumes when accounting for whole brainstem volume; Supplementary Table 5). Next, we found that for most volumes, the majority of the lead SNPs had uncorrected *P* < 0.05 in the GWAS replication sample. Moreover, as expected due to the modest sample size, only two of the lead SNPs reached the *P* < 5e−8 threshold in the replication sample (both associated with pons volume). Finally, we found that the discovery and replication GWAS for all volumes were significantly genetically correlated (all *R*_*g*_ > 0.73; Supplementary Table 5).

### Functional annotation of discovery GWAS loci

We functionally annotated SNPs across the brainstem volumes that were in linkage disequilibrium (*r*^2^ ≥ 0.6) with one of the independent significant SNPs with *P* < 5e−8 in the discovery sample using FUMA. A majority of these SNPs were intronic (60.3%) or intergenic (23.7%) and 1.5% were exonic (Supplementary Data 5–9). About 94% of the SNPs had a minimum chromatin state of 1–7, thus suggesting they were in open chromatin regions^27^. Supplementary Fig. 6 provides information for functional SNP categories for each brainstem volume. Two of the lead SNPs were exonic and associated with medulla oblongata (rs13107325) and whole brainstem (rs13388394) volumes. The combined annotation-dependent depletion (CADD) scores of those SNPs were 23.1 (rs13107325) and 17.7 (rs13388394), thus indicating deleterious protein effects^28^. rs13107325 is located in *SLC39A8* and has previously been associated with multiple traits, including SCZ and PD^29^.

### Implicated genes and genome-wide gene-based associations

We used positional, expression quantitative trait loci (eQTL), and chromatin interaction mapping in FUMA^26^ to map the 125 independent significant SNPs with *P* < 5e−8 of the discovery sample to genes. These three strategies identified 280 unique genes, where 168 of these were implicated by one mapping strategy, 68 genes by two strategies, and 25 of the genes were implicated by three strategies (Fig. 1d, and Supplementary Data 10). Supplementary Fig. 7 provides visualization of mapped genes for each brainstem volume in Circos plots.

**Fig. 2:**
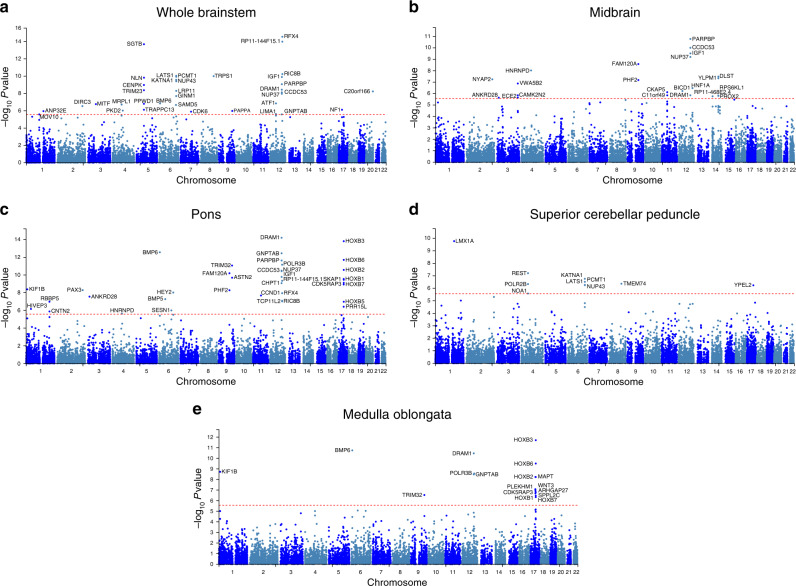
Manhattan plots from the genome-wide gene-based association analyses for volumes of the whole brainstem. **a** midbrain **b** pons **c** superior cerebellar peduncle **d**, and medulla oblongata **e** in the discovery sample. Thirty-six genes were associated with whole brainstem volume and 22, 37, 10, and 17 genes were associated with volumes of the midbrain, pons, superior cerebellar peduncle, and the medulla oblongata, respectively. Twenty-two of the genes were only associated with whole brainstem volume, whereas 13, 14, 6, 5 genes were only significant for volumes of the midbrain, pons, superior cerebellar peduncle, and the medulla oblongata. The red horizontal lines indicate genome-wide significance threshold of two-sided *P* < 2.7e−6, i.e., 0.05/18,447 genes.

We then conducted genome-wide gene-based association analyses (GWGAS; *P* < 2.7e−6, i.e., 0.05/18,447 genes) using MAGMA v1.07^30^ and detected 87 unique genes across the brainstem volumes (Fig. 2 and Supplementary Data 11). Thirty-six were associated with whole brainstem volume and 22, 37, 10, and 17 genes were associated with volumes of the midbrain, pons, SCP, and the medulla oblongata, respectively. Twenty-two were only associated with whole brainstem volume, whereas 13, 14, 6, 5 genes were only significant for midbrain, pons, SCP, and the medulla oblongata volumes. Supplementary Fig. 8 provides Q–Q plots for these GWGAS. We also found that 25 of the genes identified by GWGAS were not mapped by the GWAS analyses, resulting in a total number of 305 brainstem-linked genes identified by either GWAS or GWGAS. Moreover, supporting robustness, 17 of the 87 genes identified by the GWGAS were also implicated by all three FUMA mapping strategies (Fig. 1d, Supplementary Table 6).

We also mapped independent SNPs significant at the *P* < 1e−8 threshold (corrected for analyses of five volumes) by positional, eQTL, and chromatin interaction mapping and identified 96, 57, and 100 genes, respectively (Supplementary Data 12). GWGAS detected 66 unique genes across the brainstem volumes when applying a significance threshold of *P* < 5.4e−7 (i.e., 2.7e−6/5 volumes; Supplementary Fig. 9 and Supplementary Data 13). Fifteen were only associated with whole brainstem volume, whereas 6, 12, 4, and 5 genes were only significant for midbrain, pons, SCP, and medulla oblongata volumes, respectively.

### Gene sets implicated by the significant genes

We conducted gene-sets analyses for the genes prioritized in the GWAS discovery sample (hypergeometric tests based on independent significant SNPs with *P* < 5e−8) and identified seven Gene Ontology sets significantly associated with whole brainstem volume, and 2, 8, 1, and 15 gene sets associated with volumes of the midbrain, pons, SCP, and medulla oblongata, respectively, after Bonferroni correction (Supplementary Table 7). The most significant gene set for whole brainstem volume was susceptibility to natural killer cell mediated cytotoxicity (*P* = 1.32e−6), amyloid beta formation for midbrain (*P* = 0.01), skeletal system morphogenesis for pons (*P* = 4.6e−5), de novo imp biosynthetic process for SCP (*P* = 0.03), and embryonic skeletal system development for medulla oblongata (*P* = 1.0e−7). Notably, *HOX* genes, which encode transcription factors with central roles in nervous system development^31^ were included in seven of the significant gene sets for pons and in all gene sets associated with medulla oblongata. We also employed the ConsensusPathDB^32^ to identify overrepresented pathways for the mapped genes and found 13 significant pathways for whole brainstem volume, and 1, 25, and 58 significant for pathways for pons, SCP, and medulla oblongata volume, after false discovery rate (FDR)-correction (Supplementary Table 8).

The gene-sets analyses and the pathway analyses were also run with prioritized genes from the discovery GWAS sample when adjusting for five volumes (based on independent significant SNPs with *P* < 1e−8) and implicated the same gene sets and pathways as in the main analysis (based on independent significant SNPs with *P* < 5e−8). Ten Gene Ontology gene sets were associated with whole brainstem volume, and 1, 6, and 16 gene sets were associated with volumes of the midbrain, pons, and medulla oblongata, respectively (Supplementary Table 9). ConsensusPathDB^32^ identified 53, 19, 1, and 3 significant pathways for volumes of the whole brainstem, midbrain, pons, and medulla oblongata, respectively (Supplementary Table 10).

### Genetic overlap between volumes and common brain disorders

**Fig. 3:**
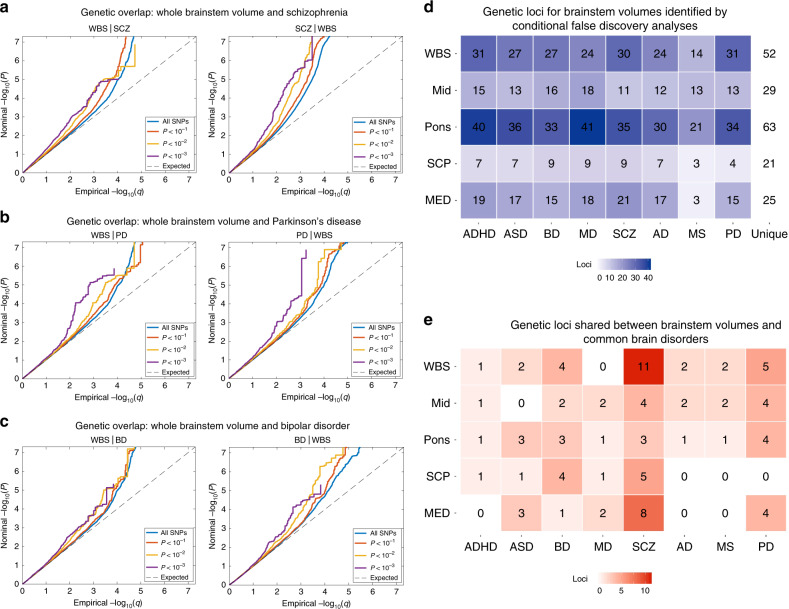
Genetic overlap between brainstem volumes and common brain disorders. **a** Conditional Q–Q plots for whole brainstem volume conditioned on SCZ (left) and vice versa (right), demonstrating genetic overlap. **b** Conditional Q–Q plots for whole brainstem volume conditioned on PD (left) and vice versa (right), showing genetic overlap between these phenotypes. **c** Conditional Q–Q plots for whole brainstem volume conditioned on BD (left) and vice versa (right), demonstrating genetic overlap. **d** Enhanced discovery of genetic loci for each of the brainstem volumes when conditional false discovery rate analyses were run for each of the brainstem volumes conditioned on the eight brain disorders. These analyses revealed a total of 208 genetic loci for whole brainstem volume, and 111, 270, 55, and 125 loci for volumes of the midbrain, pons, SCP, and medulla oblongata, respectively. These genetic regions were located in 52 unique genetic loci for whole brainstem volume, and 29, 63, 21, and 25 unique loci for volumes of the midbrain, pons, SCP, and medulla oblongata. **e** conjunctional false discovery rate analysis detected shared genetic loci across brainstem volumes and the eight clinical conditions. The largest numbers of shared loci were found for SCZ (31), BD (14), and PD (17), whereas 8, 4, 6, 9, and 5 genetic loci were jointly shared for ASD, ADHD, MD, AD, and MS, respectively, and the brainstem volumes, when applying a conjunctional FDR threshold of 0.05. WBS whole brainstem. MID midbrain. SCP superior cerebellar peduncle. MED medulla oblongata. ADHD attention-deficit/hyperactivity disorder. ASD autism spectrum disorders. BD bipolar disorder. MD major depression. SCZ schizophrenia. AD Alzheimer’s disease. MS multiple sclerosis. PD Parkinson’s disease.

To further examine the polygenic architecture of brainstem volumes and the potential genetic overlap between brainstem regions and common brain disorders, we used GWAS summary statistics for attention-deficit/hyperactivity disorder (ADHD), autism spectrum disorder (ASD), BD, major depression (MD), SCZ, Alzheimer’s disease (AD), MS, and PD, as outlined in Methods. We then generated conditional Q–Q plots^33–35^ for the brainstem regions and the eight clinical conditions. The conditional Q–Q plots compare the association with one trait (e.g., whole brainstem volume) across all SNPs and within SNPs strata determined by the significance of their association with another trait (e.g., SCZ). Polygenic overlap exists if the proportion of SNPs associated with the first trait increases as a function of the strength of association for the second trait and is visualized as a successive leftward deflection from the null distribution^33^. The conditional Q–Q plots for brainstem volumes and the clinical conditions showed successive increments of SNP enrichment for whole brainstem, midbrain, pons, SCP, and medulla oblongata (Supplementary Fig. 10), consistent with polygenic overlap across volumes and disorders. Conditional Q–Q plots illustrating the genetic overlap between whole brainstem volume and SCZ, BD, and PD are provided in Fig. 3a–c.

We leveraged the genetic overlap to discover more of the genetic underpinnings of brainstem volumes by employing conditional FDR statistics^33–35^. The conditional FDR builds on an empirical Bayesian statistical framework, combines summary statistics from a trait of interest with those of a conditional trait, and thus increases power to detect genetic variants associated with the primary trait. We ran the conditional FDR analyses for each of the brainstem volumes conditioned on the eight disorders and discovered a total of 208 genetic loci for the whole brainstem, and 111, 270, 55, and 125 loci for the midbrain, pons, SCP, and medulla oblongata, respectively. These regions were located in 52 unique genetic loci for whole brainstem volume, and 29, 63, 21, and 25 unique loci for volumes of the midbrain, pons, SCP, and medulla oblongata, respectively (Fig. 3d, Supplementary Data 14–18). The loci identified by the conditional FDR included all brainstem-associated genetic regions detected in the GWAS discovery sample. Supplementary Fig. 11 provides Manhattan plots for the genetic loci detected by the conditional FDR analyses for each brainstem region.

**Fig. 4:**
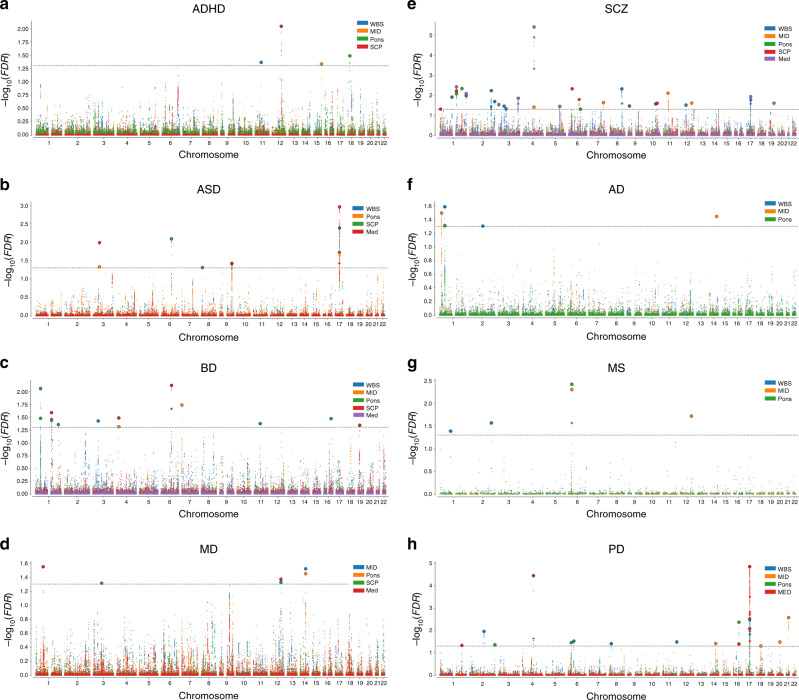
Manhattan plots for genetic loci shared between brainstem volumes and eight common brain disorders. **a** 4 shared loci in ADHD, **b** 9 shared loci in ASD, **c** 14 shared loci in BD, **d** 6 shared loci in MD, **e** 31 shared loci in SCZ, **f** 5 shared loci in AD, **g** 5 shared loci in MS, and **h** 17 shared loci in PD. WBS whole brainstem. MID midbrain. SCP superior cerebellar peduncle. MED medulla oblongata. ADHD attention-deficit/hyperactivity disorder. ASD autism spectrum disorders. BD bipolar disorder. MD major depression. SCZ schizophrenia. AD Alzheimer’s disease. MS multiple sclerosis. PD Parkinson’s disease. The black horizontal lines indicate conjunctional false discovery rate threshold of 0.05.

To further characterize the genetic overlap between brainstem volumes and the eight clinical conditions, we performed conjunctional FDR analyses, which enable detection of genetic loci shared between two phenotypes^33–35^. These analyses revealed shared loci across the brainstem structures and the clinical conditions (Fig. 3e). We found the largest number of loci shared between brainstem volumes and SCZ (31), BD (14), and PD (17). For ASD, ADHD, MD, AD, and MS, there were 9, 4, 6, 5, and 5 genetic loci jointly associated with the brainstem volumes and the disorders, respectively, when applying a conjunctional FDR threshold of 0.05 (Fig. 3e). Notably, the shared genetic loci exhibited a mixed pattern of allelic effect directions, i.e., disorder-linked genetic variants were associated with both larger and smaller brainstem volumes (Supplementary Fig. 12). No definitive conclusions can be drawn about effect directions for each disorder due to the modest number of shared loci, yet the majority of loci for SCZ showed opposite effect directions (74%; i.e., disorder-linked variants were associated with reduced volumes), whereas the majority of loci for PD showed same effect directions (70%; i.e., disorder-linked variants were associated with increased volumes). Manhattan plots and details for the genetic loci shared between the eight clinical conditions and the brainstem volumes are provided in Fig. 4a–h and in Supplementary Data 19. When using a conjunctional FDR threshold of 0.01 (corrected for five volumes), there were genetic loci jointly associated with the brainstem volumes and BD (2), SCZ (10), ASD (3), MS (2), ADHD (1), and PD (6) and no shared loci for MD and AD (Supplementary Data 19).

We ran Gene Ontology gene-sets analyses for genes nearest to the shared loci significant at a conjunctional FDR threshold of 0.05 across the brainstem regions for each disorder and found 33 significant gene sets for SCZ, mainly involving central nervous system, neuronal, and cellular developmental processes (Supplementary Table 11). There were no significant gene sets for the other disorders.

We also examined genetic correlations between brainstem volumes and the common brain disorders using LD score regression v1.0.0^36^ (Supplementary Fig. 13). There were correlations with uncorrected *P* < 0.05, including positive associations between brainstem volumes and PD, yet these were not significant after multiple testing corrections.

### Brainstem volumes in common brain disorders

**Fig. 5:**
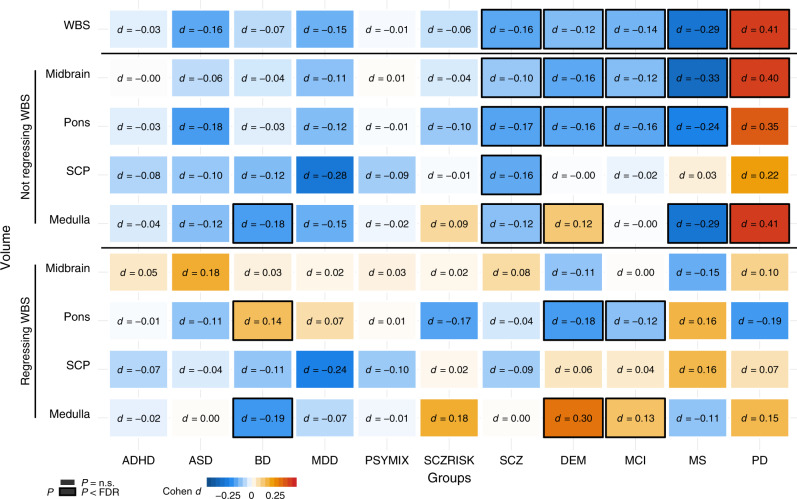
Volumes of brainstem structures in individuals with common brain disorders compared with healthy controls. Linear models were run covarying for sex, age, age², intracranial volume, and scanner site. The analyses for volumes of midbrain, pons, SCP, and medulla oblongata were run both with and without covarying for whole brainstem volume. The figure depicts the resulting case-control differences in Cohen’s d, whereas group differences in mm^3^ are presented in Supplementary Fig. 14. There were differential volumetric alterations in individuals with BD, SCZ, DEM, MCI, MS, and PD as indicated by black frames, after controlling for multiple testing using false discovery rate (Benjamini–Hochberg, accounting for all 99 tests). ADHD attention-deficit/hyperactivity disorder. ASD autism spectrum disorders. BD bipolar disorder. MDD major depressive disorder. PSYMIX non-SCZ psychosis spectrum diagnoses. SCZRISK prodromal SCZ or at risk mental state. SCZ schizophrenia. DEM dementia. MCI mild cognitive impairment. MS multiple sclerosis. PD Parkinson’s disease. WBS whole brainstem. SCP superior cerebellar peduncle. Medulla medulla oblongata.

We compared brainstem volumes between individuals with common brain disorders and HC (age range 3–96 years): ADHD (*n* = 681 patients/*n* = 992 HC), ASD (*n* = 125/*n* = 140), BD (*n* = 464/*n* = 1513), major depressive disorder (MDD; *n* = 211/*n* = 93), SCZ (*n* = 1044/*n* = 2079), prodromal SCZ or at risk mental state (SCZRISK; *n* = 91/*n* = 402), non-SCZ psychosis spectrum diagnoses (PSYMIX; *n* = 308/*n* = 1430), dementia (*n* = 756/*n* = 1921), MCI (*n* = 987/*n* = 1655), MS (*n* = 257/*n* = 1053), and PD (*n* = 138/*n* = 67). Supplementary Tables 1–3 provide information on the individual cohorts. Linear models were run covarying for sex, age, age², ICV, and scanner site using R 3.5^37^. The analyses for volumes of midbrain, pons, SCP, and medulla oblongata were run both with and without covarying for whole brainstem volume, and were adjusted for multiple testing using FDR (Benjamin–Hochberg, accounting for all 99 tests). Figure 5 depicts the resulting case-control differences in Cohen’s d, whereas group differences in mm^3^ and scatter plots are presented in Supplementary Figs. 14–15.

BD was associated with smaller medulla oblongata volume and larger pons volume, when accounting for the whole brainstem. Individuals with SCZ showed smaller volumes of all brainstem structures compared with HC, but not significantly for the midbrain, pons, and medulla oblongata when regressing out whole brainstem volume, consistent with a general effect across the brainstem regions. Volumes of whole brainstem, midbrain, and pons were smaller in the individuals with dementia compared with HC, whereas medulla oblongata volume was larger. A highly similar pattern was found for individuals with MCI, with smaller volumes of the whole brainstem, midbrain, and pons, and larger medulla oblongata volume when accounting for whole brainstem. Individuals with MS showed smaller volumes of the whole brainstem, midbrain, pons, and medulla oblongata, whereas individuals with PD had larger volume of the whole brainstem, midbrain, and medulla oblongata.

We ran further analyses of associations between brainstem volumes and clinical characteristics in the individuals with MCI, dementia, MS, SCZ, and PD and details of these analyses are provided in Supplementary Figs. 16–17 and Supplementary Note 1. There were significant associations between Mini-Mental State Examination scores and brainstem volumes in dementia and MCI, indicating smaller pons and larger medulla oblongata volumes in more severely affected individuals (linear models; all *P* < 2e−04). In MS, there were brainstem volume decreases also in the subgroup of patients without infratentorial lesions (linear models; *n* = 91; all *P* < 0.05) and significant negative associations between the Expanded Disability Status Scale scores and brainstem volumes in patients with infratentorial lesions (linear models; *n* = 153; *P* < 0.05). There was no significant association between the Global Assessment of Functioning scale or Positive and Negative Syndrome Scale scores and brainstem volumes in individuals with SCZ. We found no evidence for tremor severity influencing brainstem volumes in individuals with PD.

## Discussion

The midbrain, pons, and medulla oblongata have central roles in human health and disease, yet no large-scale neuroimaging study has focused on their structure and genetic underpinnings. Here, we discovered novel genetic loci associated with brainstem volumes and found genetic overlap with eight psychiatric and neurological disorders, revealing that these brainstem regions may play important roles in common brain disorders. Indeed, leveraging clinical imaging data we found differential alterations of midbrain, pons, and medulla oblongata volumes in individuals with SCZ, BD, MS, dementia, MCI, and PD.

We identified 61 genetic loci associated with brainstem volumes at a statistical threshold of *P* < 5e−8. Sixteen of the loci were associated with more than one volume, thus resulting in 45 unique brainstem-associated genetic regions. Twenty-nine of them were associated with volumes of the individual brainstem regions and not with whole brainstem volume. Thirty-four of the 45 unique loci remained significant at a threshold of *P* < 1e−8, i.e., adjusted for analyses of five volumes.

There is to our knowledge no previous study of the genetic underpinnings of midbrain, pons, SCP, and medulla oblongata volumes, yet a study of 3144 functional and structural brain imaging phenotypes in ~8400 individuals from UK Biobank identified four loci associated with Freesurfer-based volume of the whole brainstem^17^. These SNPs are within four of the sixteen genetic loci linked to whole brainstem volume in the present study. One recently published large-scale GWAS identified 48 genetic loci associated with seven subcortical brain volumes and 16 of these were linked to the whole brainstem^18^. Twelve of them overlap with genetic regions linked to volume of the whole brainstem in the current study. Another recent GWAS of 101 brain phenotypes detected 6 and 15 genetic loci associated with whole brainstem volume at thresholds of *P* < 4.9e−10 (adjusted for analyses of 101 phenotypes) and *P* < 5e−8, respectively^19^ and 9 of the latter are within regions associated with whole brainstem volume in the present study. Three genetic loci linked to whole brainstem volume in the current study on chromosomes four (rs10939607), nine (rs418382), and 12 (rs11111091) overlap with genetic loci significant in all of the three previous works. Four of the whole brainstem-associated loci identified by the current study on chrosomomes one (rs35536968 and rs698915), two (rs13388394), and six (rs75343521) have not been reported previously.

The GWAS identified ten genetic loci linked to midbrain volume at *P* < 5e−8. The most significant midbrain-associated genetic locus was found on chromosome 7 (rs151057105), which was associated with malignant brain tumor and well-being in previous studies^38,39^. *IGF1* was among the genes most strongly associated with midbrain volume in the GWGAS of the current study. *IGF1* encodes a growth factor in the brain which regulates synaptic plasticity and neurogenesis and affects all major neural cells during development^40^.

We detected 23 and 9 genetic loci associated with volumes of pons and medulla oblongata at *P* < 5e−8, respectively. The genetic loci most strongly associated with pons and medulla oblongaa volumes was located on chromosome 12 (rs11111091). Notably, rs11111091 is a SNP identified in one of three loci linked to whole brainstem volume across the present and three previous studies^17–19^. The gene nearest rs11111091 is *DRAM1*, which encodes an autophagy and apoptosis-regulating protein of the p53 tumor suppressor pathway^41^. We also found that *HOX* genes were included in the nine most significant Gene Ontology gene sets for pons and in the 24 gene sets most strongly associated with medulla oblongata volume. In addition, nine *HOX* genes (*HOXB1-9*) were associated with volumes of both pons and medulla oblongata in the GWGAS. *HOX* genes encode Hox proteins, which are transcription factors with central roles in nervous system development^42^. The *HOXB1-4* genes are critical for the development of the embryonic hindbrain, which gives rise to the pons, the medulla oblongata, and the cerebellum^31^. The *HOX* genes are not, however, expressed in the embryonic midbrain, which develops into the midbrain. Consistent with the embryonic genetic division between the hindbrain and the midbrain, *HOX* genes were not associated with the midbrain in the gene sets or in the GWGAS analyses of the current study.

We found three genetic loci associated with SCP volume at *P* < 5e−8. The most strongly associated SCP-linked locus was located on chromosome 1 (rs11809085). The gene nearest this locus—*LMX1A*—was also the most strongly SCP-associated gene in the GWGAS. *LMX1A* codes for a growth factor that is involved in development of brainstem and cerebellum structures and has been linked to psychiatric and neurological disorders, including SCZ and PD^43^. *REST* was the second-most strongly SCP-associated gene in the GWGAS in the present study. *REST* encodes a transcriptional repressor with neuroprotective properties in normal aging and *REST*-dysfunction has been linked to neurodegenerative disorders, including AD^44^.

There was polygenic overlap between the brainstem regions and the eight psychiatric and neurological disorders of the present study. We leveraged the genetic overlap to uncover more of the genetic architecture of the brainstem volumes and identified 52, 29, 63, 21, and 25 loci associated with volumes of the whole brainstem, midbrain, pons, SCP, and medulla oblongata, respectively, using conditional FDR. These loci included all brainstem-associated genetic regions identified by the GWAS. The polygenic overlap also indicates a role for brainstem regions in common brain disorders and gene-sets analyses implicated cellular and neurodevelopmental processes in the genetic loci shared with SCZ.

Further studies of how the overlapping genetic regions influence brainstem structure and the risk for common brain disorders are warranted, yet several of the shared loci are noteworthy. The most significant shared locus for SCZ and the second-most significant shared locus for PD was rs13107325, which was associated with midbrain volume in SCZ and medulla oblongata volume in both disorders. rs13107325 is located in the metal ion transporter gene *SLC39A8*. We also found that rs4845679 was jointly associated with volumes of pons, SCP, and medulla oblongata and both SCZ and BD. The nearest gene for rs4845679 is *KCNN3*, which is expressed at high levels in the adult brain and encodes a protein that contributes to the afterhyperpolarization in neurons^45^. rs8070942 and rs3865315 were shared between ASD and SCZ, respectively, and medulla oblongata volume. The nearest gene for these SNPs was *KANSL1*, which is expressed in the brain and encodes a nuclear protein involved in histone acetylation^46^.

We also found that the genetic loci shared between brainstem structures and the brain disorders exhibited a mixed pattern of allelic effect directions, i.e., disorder-linked genetic variants were associated with both larger (same effect direction) and smaller (opposite effect direction) brainstem volumes. A consistent direction of effect across overlapping genetic loci is a requirement for a significant genetic correlation as assessed using LD score regression^36^. For example, a recent study showed that SCZ and educational attainment may share >8 K causal genetic variants, yet their genetic correlation is close to zero due to shared variants with opposite effect directions^47^. Thus, a mixed pattern of allelic effect directions might be one explanation for the lack of robust genetic correlations between the brainstem volumes and the disorders in the present study.

We detected brainstem volume differences between individuals with SCZ, BD, dementia, MCI, MS, and PD and their respective HC groups. The monoaminergic nuclei of the brainstem are implicated in psychotic and mood disorders^4,48^, yet there are few volumetric studies of brainstem regions in these illnesses. The results of the present study suggest a general volume decrease across brainstem regions in SCZ, consistent with previous studies of the whole brainstem^49,50^. BD, on the other hand, was associated with reduced volume of the medulla oblongata and a relative sparing or even increase of pons volume in the current study. Whether brainstem differences in SCZ and BD are genetically mediated and involved in the development of these disorders or illness effects that emerge during the course of the diseases mandates future studies. We do however note that a majority of the genetic loci shared between SCZ and brainstem volumes in the current study had opposite effects directions, i.e., disorder-linked variants were associated with reduced volumes. This finding is suggestive of genetic contributions to brainstem volume decreases in SCZ.

Individuals with dementia had reduced volumes of the midbrain and pons and increased relative volume of medulla oblongata. Notably, we found a highly similar pattern in individuals with MCI. To our knowledge, there is no previous study showing reduced brainstem volumes in MCI, although one recent report found greater whole brainstem volume reduction over one year in individuals with MCI that converted to dementia than in those who did not convert^51^. There is a scarcity of structural brainstem studies in dementia, yet the results of the present study are consistent with a few previous findings suggesting volume decreases mainly in midbrain and pons in dementia^20,52^. Here, we extend these findings to MCI, thus suggesting that structural midbrain and pons alterations could be present in the early phases of dementia. The smaller volumes of whole brainstem, midbrain, pons, and the medulla oblongata in individuals with MS are consistent with the limited number of previous volumetric brainstem studies of the disorder^53,54^.

We found larger volumes of the whole brainstem, midbrain, and medulla oblongata in the individuals with PD. There was no indication that tremor severity could explain the volume increases. Notably, some previous studies detected enlargement of the brainstem and other brain structures in PD^55,56^ and the individuals with PD of the present study were in the early phase of the disorder and none used anti-Parkinson drugs. Furthermore, the majority of loci jointly associated with PD and brainstem volumes in the present study showed same effect directions, i.e., disorder-linked variants were associated with increased volumes. Although no definitive conclusions can be drawn due to the modest number of shared loci, this may suggest that brainstem volume increases in PD, if present, may be at least partly genetically mediated. However, the PD sample was small and replication studies are needed to further explore how genetic variants, clinical characteristics, and potential confounds, including within-scanner motion, may factor into measurements of brainstem volumes in PD.

In summary, the current study provides new insights into the genetic architecture of brainstem regions, identifies the first genetic loci linked to volumes of the midbrain, pons, SCP, and the medulla oblongata, and shows genetic and imaging evidence for an involvement of brainstem regions in common brain disorders. Altogether, these findings encourage further studies of brainstem structures in human health and disease.

## Methods

### Samples

We collected data from a total of *n* = 57,298 individuals, obtained through collaborations, data sharing platforms, and from in-house samples. All included samples have been part of previously published works and data collection for each sample was performed with participants’ written informed consent and with approval by the respective local Institutional Review Boards. Supplementary Table 1 provides details for each sample and refers to previously published works from the included samples.

### Preprocessing of MRI data, brainstem segmentations, and quality control procedures

Raw T1-weighted MRI data for all individuals was stored and analyzed locally at the University of Oslo. The whole brainstem, midbrain, pons, SCP, and medulla oblongata were then delineated using Freesurfer 6.0^22^ and Bayesian brainstem segmentation^20^. The brainstem segmentation method is based on a probabilistic atlas and Bayesian inference and is robust to changes in MRI scanners and pulse sequence details^20^. We then manually assessed the delineations in all MRI data sets (*n* = 57,298) by visually inspecting twelve sagittal view figures of the segmentations for each participant, as shown in Supplementary Fig. 1. This visual QC procedure for each data set was conducted blind to case-control status. Data sets were excluded from the study if one of the following requirements was not met: (1) the field of view included the whole brainstem, (2) the superior boundary of the midbrain approximated an axial plane through the mammillary body and the superior edge of the quadrigeminal plate, (3) the boundary between midbrain and pons approximated an axial plane through the superior pontine notch and the inferior edge of the quadrigeminal plate, (4) the boundary between pons and medulla oblongata approximated an axial plane at the level of the inferior potine notch, (5) the inferior boundary of the medulla oblongata approximated an axial plane at the level of the posterior rim of the foramen magnum, (6) there were no substantial segmentation errors for the anterior and posterior boundaries of midbrain, pons, and medulla oblongata, and (7) the superior boundary of the SCP approximated the inferior boundary of the midbrain tectum, the inferior boundary of the SCP was defined by the merging with the cerebellum, and the anterior boundary of the SCP was defined by the posterior boundary of the pons.

This QC procedure excluded 11.4% (*n* = 6513) of the data sets, mainly due to insufficient field of view (e.g., not fully covering the inferior part of the medulla oblongata), insufficient data quality, and segmentation errors in the clinical samples, resulting in a final sample size of *n* = 50,785. These comprised three main samples (Supplementary Table 3): (1) a GWAS discovery sample with 27,034 genotyped individuals from the UK Biobank; (2) a GWAS replication sample with 7432 additional genotyped individuals from the UK Biobank, and (3) a clinical sample of individuals with psychiatric or neurological disorders and HC (5062 patients and 11,257 controls).

### Genome-wide association studies for brainstem volumes and identification of genomic loci

We conducted GWAS on MRI and genetic data from the participants in the GWAS discovery and replication samples. We restricted all genetic analyses to individuals with White European ancestry, as determined by the UK Biobank study team. We applied standard quality control procedures to the UK Biobank v3 imputed genetic data, removing SNPs with an imputation quality score <0.5, a minor allele frequency <0.05, missing in more than 5% of individuals, and failing the Hardy Weinberg equilibrium tests at a *P* < 1e−6.

GWAS was run for the brainstem volumes in the discovery and replication samples using PLINK 2.0^23^. All GWAS accounted for age, age², sex, scanning site, ICV, genetic batch, and the first ten genetic principal components to account for population stratification. In addition, the GWAS for the midbrain, pons, SCP, and medulla oblongata was run both with and without accounting for whole brainstem volume. The MHC region was excluded from the analysis. To account for potential effects of relatedness in the discovery sample, we reran the GWAS for all brainstem volumes when excluding individuals related up to 4th degree (*n* = 705), leaving us with 26,329 individuals, and compared the GWAS results of these individuals with those of the whole discovery sample (*n* = 27,034).

We identified genetic loci related to brainstem volumes using the FUMA platform v1.3.5^26^. The settings and results of the FUMA analyses can be found at [https://fuma.ctglab.nl/browse] (FUMA ID 97–105). For these analyses, we used the 1000GPhase3 EUR as reference panel. We identified independent significant SNPs by the genome-wide significant threshold (*P* < 5e−8) and by their independency. The minimum *r*^2^ was used to determine the borders of the genomic risk loci and during the first clumping step, the SNPs that were at *r*^2^ ≤ 0.6 with each other within a 1 mb window were considered as independent significant SNPs. In the next step, independent significant SNPs with *r*^2^ < 0.1 within a 1 mb window were defined as lead SNPs. Genomic risk loci were found by merging lead SNPs if they were closer than 250 kb. Candidate SNPs were defined as all SNPs in LD (*r*^2^ ≥ 0.6) with one of the independent significant SNPs in the genetic loci. We also present findings when employing a statistical threshold of *P* < 1e−8, i.e., adjusted for analyses of five brainstem volumes.

### Functional annotation, gene-based association, and gene-set analysis

We functionally annotated all candidate SNPs of brainstem volumes that were in linkage disequilibrium (*r*^2^ ≥ 0.6) with one of the independent significant SNPs using FUMA. FUMA is based on information from 18 biological repositories and tools and functionally annotates GWAS results. The platform prioritizes the most likely causal SNPs and genes by combining positional, eQTL, and chromatin interaction mapping^26^. FUMA annotates significantly associated SNPs with functional categories, combined CADD scores^28^, RegulomeDB scores^57^, and chromatin states^26^. A CADD score above 12.37 is suggestive of a deleterious protein effect^28^. The RegulomeDB score indicates the regulatory functionality of SNPs based on eQTLs and chromatin marks. The chromatin state shows a genomic region’s accessibility for every 200 bp with 15 categorical states predicted by ChromHMM based on five histone modification marks (H3K4me3, H3K4me1, H3K36me3, H3K27me3, H3K9me3) for 127 epigenomes^27^. A lower score shows higher accessibility in the chromatin state and refers to a more open state. The 15-core chromatin states as suggested by Roadmap are as follows: 1 = Active Transcription Start Site (TSS); 2 = Flanking Active TSS; 3 = Transcription at gene 5′ and 3′; 4 = Strong transcription; 5 = Weak Transcription; 6 = Genic enhancers; 7 = Enhancers; 8 = Zinc finger genes & repeats; 9 = Heterochromatic; 10 = Bivalent/Poised TSS; 11 = Flanking Bivalent/Poised TSS/Enh; 12 = Bivalent Enhancer; 13 = Repressed PolyComb; 14 = Weak Repressed PolyComb; 15 = Quiescent/Low13^58^.

We conducted genome-wide gene-based association and gene-set analyses using MAGMA v1.07^30^ in FUMA on the complete GWAS input data. The MHC region was excluded before running the MAGMA analyses. MAGMA performs multiple linear regression to obtain gene-based *P* values and the Bonferroni-corrected significant threshold was *P* = 0.05/18158 genes = 2.75e−6. We performed a gene-set analysis using hypergeometric tests for curated gene sets and GO terms obtained from MsigDB^59^. To identify overrepresented pathways for the mapped genes, we used the ConsensusPathDB^32^. ConsensusPathDB is a database system that integrates functional interactions, including binary and complex protein–protein, genetic, metabolic, signaling, gene regulatory and drug-target interactions, as well as biochemical pathways^32^. The genome-wide gene-based association, the gene sets, and the pathway results are also presented when accounting for analyses of five brainstem volumes.

### Analyses of genetic overlap between brainstem volumes and eight brain disorders

To further examine the genetic architecture of brainstem volumes and the genetic relationships between brainstem regions and common brain disorders, we obtained GWAS summary statistics for ADHD^60^, ASD, SZ, and BD from the Psychiatric Genomics Consortium^61–63^, for MD from the Psychiatric Genomics Consortium and 23andMe^64,65^, for AD from the International Genomics of Alzheimer’s Project^66^, for MS from the International Multiple Sclerosis Genetics Consortium^67^, and for PD from the International Parkinson Disease Genomics Consortium^68,69^. We then employed conditional Q–Q plots and conditional FDR and conjunctional FDR statistics to assess polygenic overlap between brainstem volumes and the eight brain disorders using MATLAB 2017a and Python 3.7.14^33,34^. A mathematical description and review of the applications of these methods in neurological and psychiatric disorders can be found in Smeland et al.^35^.

The conditional FDR builds on an empirical Bayesian framework and uses auxiliary genetic information to re-adjust GWAS test statistics of a primary phenotype^35^. Conditional FDR includes separate GWAS data and leverages overlapping genetic associations to increase discovery of phenotype-associated SNPs. This approach asigns a conditional FDR value to each SNP which is defined as the probability that a SNP has no association with the first phenotype, given that the *P* values for the first and second phenotypes are as small or smaller than the observed *P* values^33–35^. The conditional FDR values are estimated for each nominal SNP *P* value for the first phenotype after computing the stratified empirical cumulative distribution functions of the nominal *P* values^35^.

The first step of the conditional FDR approach is to construct conditional Q–Q plots. These plots compare the association with a primary trait across all SNPs and within SNPs strata determined by their association with the secondary trait. Genetic overlap exists if the proportion of SNPs associated with a phenotype increases as a function of the strength of the association with a secondary phenotype. In conditional Q–Q plots, this enrichment is visualized as successive leftward deflections from the null distribution, and can be directly interpreted in terms of the true discovery rate (1−FDR)^33–35^. In this work, we plotted the empirical cumulative distribution of nominal *P* values in one phenotype (e.g., whole brainstem volume) for all SNPs and for subsets of SNPs with significance levels in another phenotype (e.g., SCZ) below the indicated cutoffs (*P* ≤ 1, *P* ≤ 0.1, *P* ≤ 0.01, and *P* ≤ 0.001).

To detect genetic loci jointly associated with the brainstem volumes and the eight clinical conditions, we used the conjunctional FDR method^33–35^ and present results based on both a threshold of 0.05 and a threshold of 0.01. The conjunctional FDR is an extension of conditional FDR and is defined by the maximum of the two conditional FDR values for a specific SNP. This method estimates a posterior probability that a SNP is null for either trait or both at the same time, given that the *P* values for both phenotypes are as small, or smaller, than the *P* values for each trait individually. Manhattan plots were constructed based on the ranking of the conjunctional FDR to show the genomic location of the shared genetic risk loci. The empirical null distribution in GWASs is affected by global variance inflation and all *P* values were therefore corrected for inflation using a genomic inflation control procedure. All analysis was performed after excluding SNPs in the major extended histocompatibility complex (hg19 location chromosome 6: 25119106–33854733) and 8p23.1 regions (hg19 location chromosome 8: 7242715–12483982) for all cases and *MAPT* and *APOE* regions for PD and AD, respectively, since complex correlations in regions with intricate LD can bias the FDR estimation. We also ran pairwise genetic correlations between brainstem volumes and the eight psychiatric and neurological disorders using LD score regression^36^. Here, the SNPs were pruned using a pairwise correlation coefficient approximation to LD (*r*²), where SNPs were disregarded at *r²* < 0.2 and pruning performed with 20 iterations.

### Statistical analysis of brainstem volumes, brain disorders, and clinical variables

Statistical analyses for group comparisons were conducted using linear models in R statistics^37^. We included all healthy individuals that were imaged on the same scanners as the patients they were compared with, in the respective control groups. For clinical conditions where participants were imaged on multiple scanners, we included scanner site as a covariate in the analyses. For each of the clinical conditions, we ran linear models covarying for sex, age, age², ICV, and adjusted for multiple testing using FDR (Benjamini–Hochberg). The group analyses for volumes of midbrain, pons, SCP, and medulla oblongata were run both with and without covarying for whole brainstem volume and group differences in Cohen’s d and mm^3^ are presented.

Information concerning illness severity was available from individuals with MCI, dementia, MS, SCZ, and PD. 1610 individuals with MCI or dementia had MMSE score, whereas 190 individuals with MS had EDSS scores. Linear models were run to examine the relationships between the clinical variables and brainstem volumes covarying for sex, age, age², ICV, and scanner site. Two neuroradiologists assessed the imaging data from the individuals with MS and found that *n* = 153 participants had infratentorial MS lesions detectable with MRI, whereas *n* = 91 did not. 384 individuals with SCZ had function scores of the Global Assessment of Functioning scale, whereas 264 individuals had symptom scores from the scale. 616 and 614 individuals with SCZ had positive and negative scores, respectively, from the Positive and Negative Syndrome Scale. 128 individuals with PD had Unified Parkinson’s Disease Rating Scale III scores^70^ and the Hoehn and Yahr Stage score. To examine whether tremor level might influence the measurements of brainstem volumes in PD, we used the self-report tremor item 2.10 of the Unified Parkinson’s Disease Rating Scale III and examined brainstem volumes across these tremor scores using linear models.

### Reporting summary

Further information on research design is available in the Nature Research Reporting Summary linked to this article.

## Supplementary information

Supplementary Information

Peer Review File

Description of Additional Supplementary Files

Reporting Summary

Supplementary Data 1

Supplementary Data 2

Supplementary Data 3

Supplementary Data 4

Supplementary Data 5

Supplementary Data 6

Supplementary Data 7

Supplementary Data 8

Supplementary Data 9

Supplementary Data 10

Supplementary Data 11

Supplementary Data 13

Supplementary Data 14

Supplementary Data 15

Supplementary Data 16

Supplementary Data 17

Supplementary Data 18

Supplementary Data 19

Supplementary Data 20

## Data Availability

Data used in preparation of this article were obtained from the Psychiatric Genomics Consortium [https://www.med.unc.edu/pgc/], 23andMe [https://www.23andme.com/], the International Genomics of Alzheimer’s Project [http://web.pasteur-lille.fr/en/recherche/u744/igap/igap_download.php], the International Multiple Sclerosis Genetics Consortium [http://imsgc.net/], and the International Parkinson Disease Genomics Consortium [https://pdgenetics.org/]. Data used in this article were also obtained from the UK Biobank [https://www.ukbiobank.ac.uk/], the Alzheimer’s Disease Neuroimaging Initiative (ADNI) database [adni.loni.usc.edu], from ABIDE [https://fcon_1000.projects.nitrc.org/], from ADHD200 [http://fcon_1000.projects.nitrc.org/], OASIS [http://www.oasis-brains.org/], PPMI [http://www.ppmi-info.org/], and SCHIZCONNECT [http://schizconnect.org/]. A detailed overview of the included cohorts and acknowledgement of their respective funding sources and cohort-specific details are also provided in Supplementary Table 1. The summary statistics for brainstem volumes of the GWAS discovery sample are available in the Zenodo repository [https://zenodo.org/record/3752700#.Xpb-suSm2bh/]. GWAS results are also available on the FUMA website [https://fuma.ctglab.nl/browse/; ID 97–105].

## Code availability

The codes for condFDR and conjFDR analyses are publicly available on [https://github.com/precimed/pleiofdr/]. Other codes needed to reproduce the results are available from the authors upon request.
