# Supplementary material for: The genetic architecture of human brainstem structures and their involvement in common brain disorders
Source: Nat Commun. 2020 Aug 11;11:4016. doi: 10.1038/s41467-020-17376-1 (PMC7421944; doi:10.1038/s41467-020-17376-1)
Supplement: Supplementary file 1 — Supplementary Information [file 41467_2020_17376_MOESM1_ESM.pdf]

Supplementary Information

for

The genetic architecture of human brainstem structures and their involvement in common brain disorders

by Elvsåshagen and Bahrami et al.

The Supplementary Information includes:

Supplementary Figures 1-17 on pages 2-31.

Supplementary Note 1 on page 32.

Supplementary Tables 1-11 on pages 33-55.

Supplementary References on pages 56-58.

Supplementary Figures

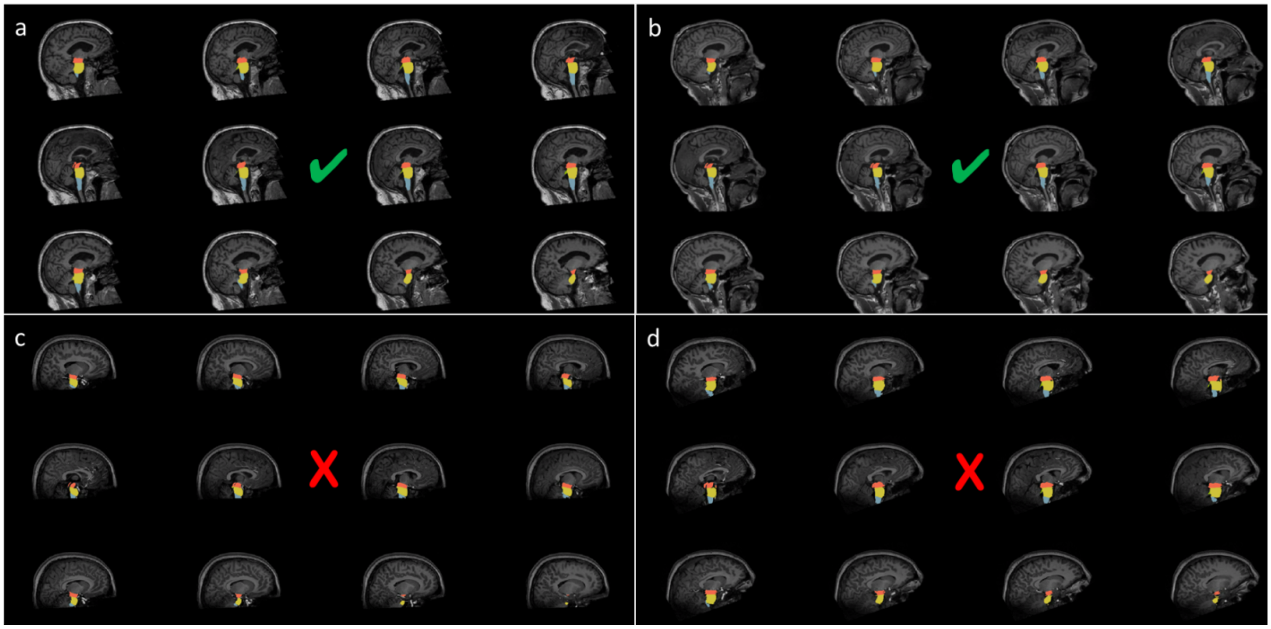

Supplementary Fig. 1 | We manually assessed the delineations in all magnetic resonance imaging data sets (*n* = 57,298) by visually inspecting twelve sagittal view figures of the segmentations for each participant, as illustrated in **a**-**d**. **a** and **b** are examples of two datasets included in the study, whereas **c** and **d** are data sets excluded due to insufficient field of view (FOV). Data sets were excluded from the study if one of the following requirements was not met: 1. the FOV included the whole brainstem, 2. the superior boundary of the midbrain approximated an axial plane through the mammillary body and the superior edge of the quadrigeminal plate, 3. the boundary between mibrain and pons approximated an axial plane through the superior pontine notch and the inferior edge of the quadrigeminal plate, 4. the boundary between between pons and medulla oblongata approximated an axial plane at the level of the inferior potine notch, 5. the inferior boundary of the medulla oblongata approximated an axial plane at the level of the posterior rim of the foramen magnum, 6. there were no substantial segmentation errors for the anterior and posterior boundaries of midbrain, pons, and medulla oblongata, and 7. the superior boundary of the SCP approximated the inferior boundary of the midbrain tectum, the inferior boundary of the SCP was defined by the merging with the cerebellum, and the anterior boundary of the SCP was defined by the posterior boundary of the pons. This visual quality control procedure excluded 11.4% (*n* = 6,513) of the data sets, mainly due to insufficient FOV, image quality, and segmentation errors in the clinical sample. SCP; superior cerebellar peduncle.

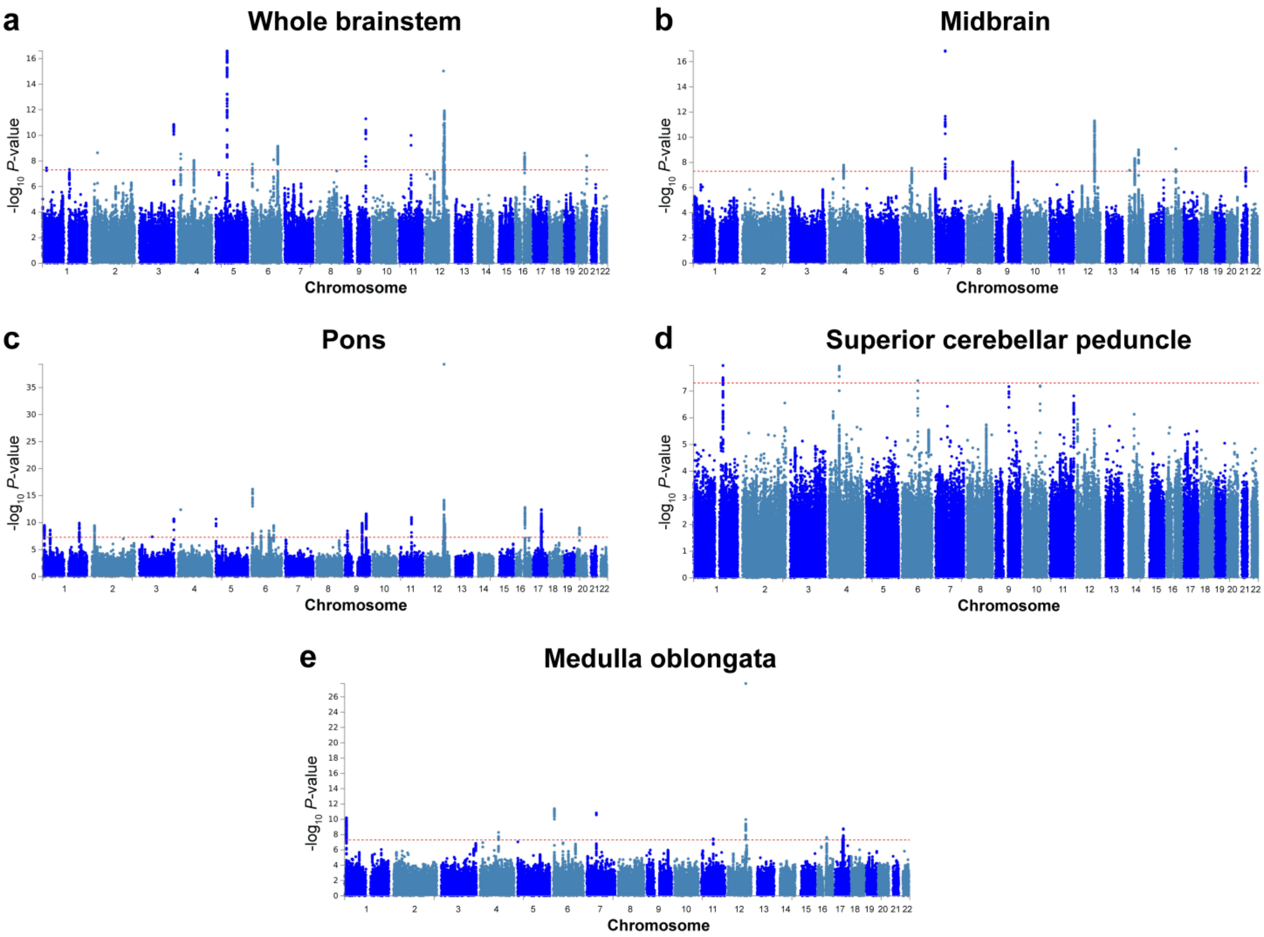

Supplementary Fig. 2 | Manhattan plots for volumes of the whole brainstem (**a**), midbrain (**b**), pons (**c**), superior cerebellar peduncle (d), and medulla oblongata (e) from the genome-wide association studies. 16 genetic loci were associated with whole brainstem volume and 10, 23, 3, and 9 loci were associated with volumes of the midbrain, pons, superior cerebellar peduncle, and medulla oblongata, respectively. The red horizontal lines indicate genome-wide significance (two-sided $P < 5e\text{-}8$).

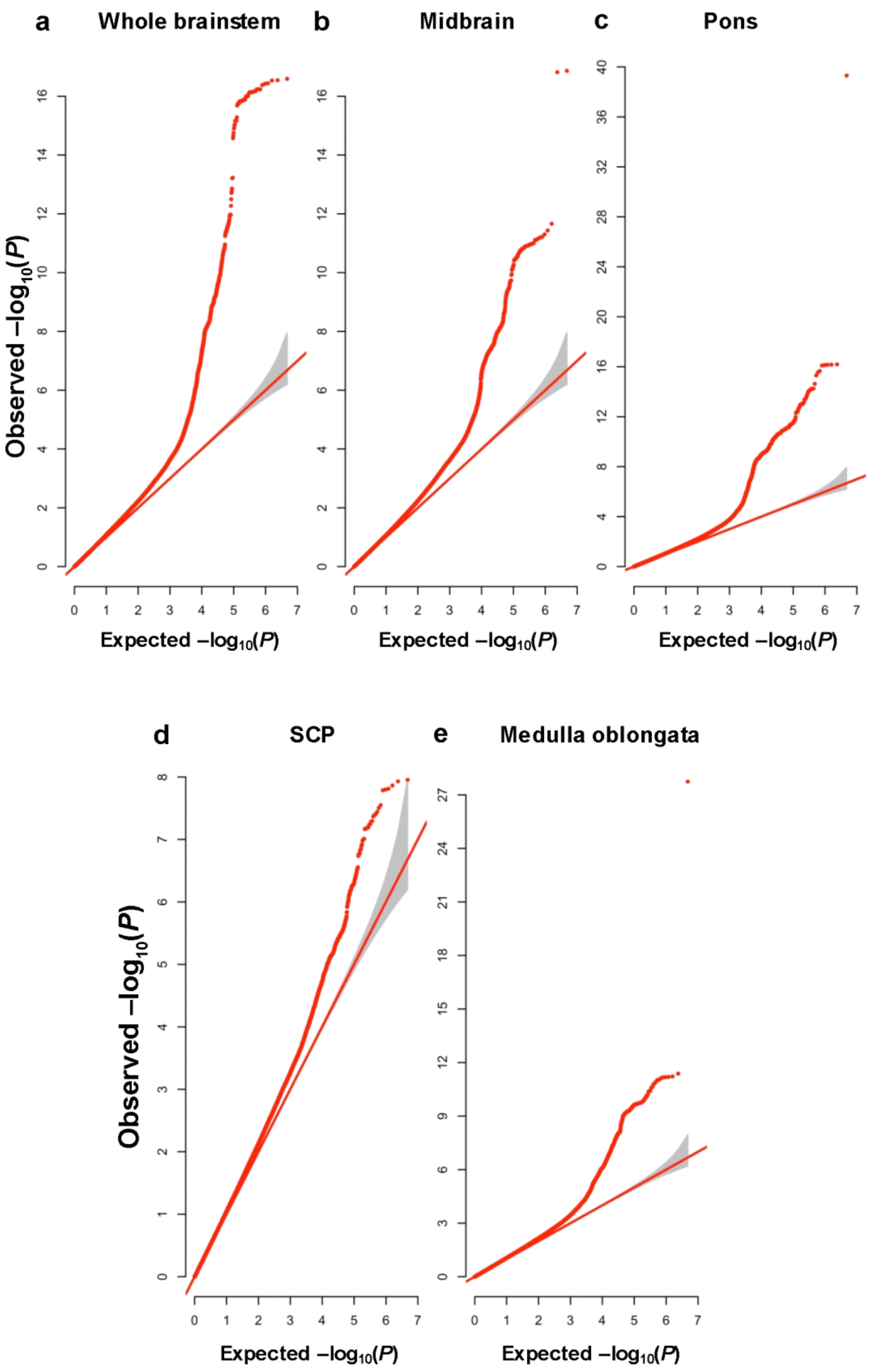

Supplementary Fig. 3 | Q-Q plots for volumes of the whole brainstem (**a**), midbrain (**b**), pons (**c**), superior cerebellar peduncle (**d**), and medulla oblongata (**e**) from the genome-wide association studies. SCP; superior cerebellar peduncle.

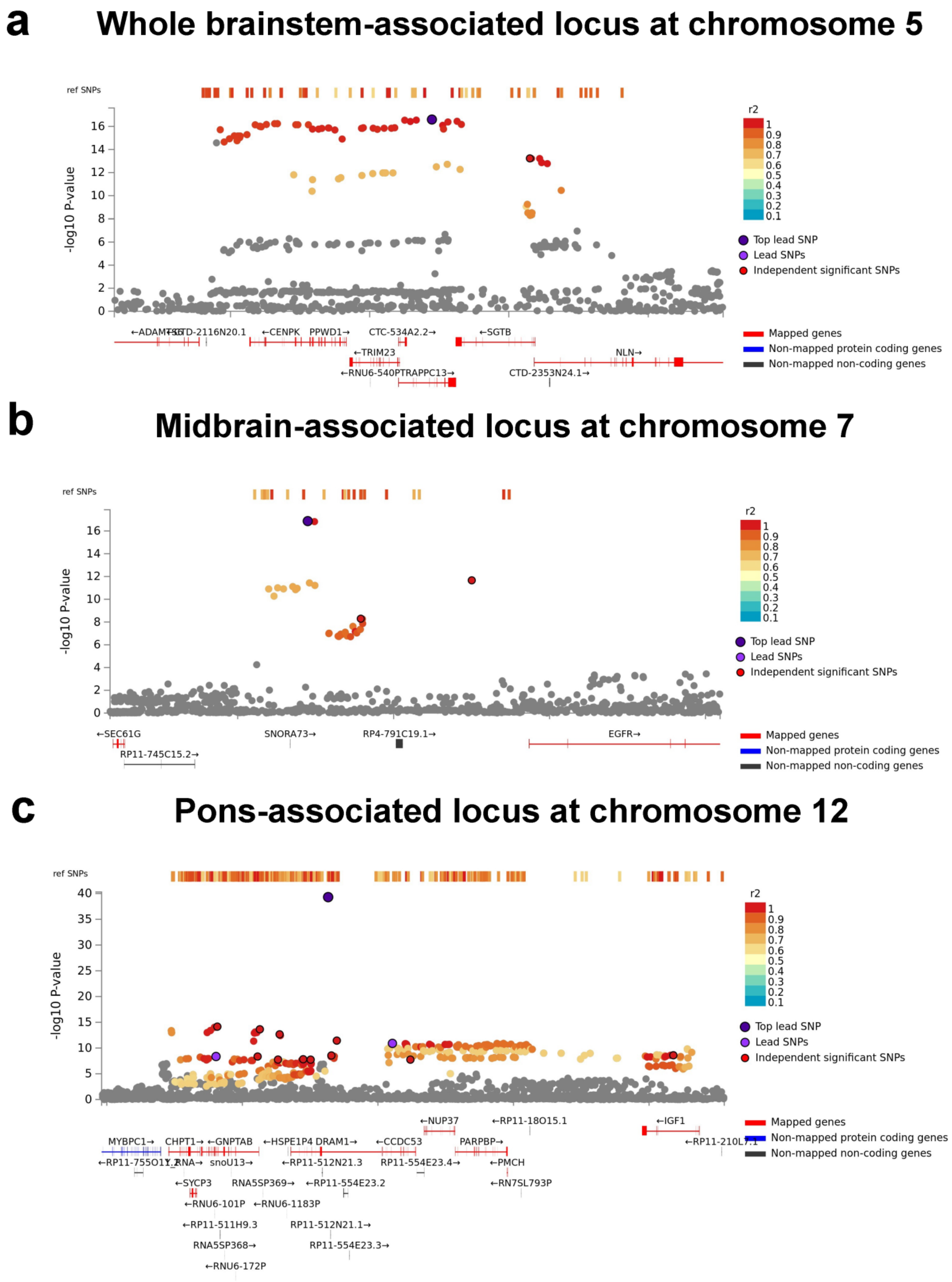

a
Whole brainstem-associated locus at chromosome 5
ref SNPs
-log10 P-value
r2
Top lead SNP
Lead SNPs
Independent significant SNPs
←ADAMTS6
CTD-2116N20.1
←CENPK
PPWD1→
CTC-534A2.2→
←SGTB
←TRIM23
NLN→
←RNU6-540P
TRAPPC13→
CTD-2353N24.1→
Mapped genes
Non-mapped protein coding genes
Non-mapped non-coding genes
b
Midbrain-associated locus at chromosome 7
ref SNPs
-log10 P-value
r2
Top lead SNP
Lead SNPs
Independent significant SNPs
←SEC61G
SNORA73→
RP4-791C19.1→
EGFR→
RP11-745C15.2→
Mapped genes
Non-mapped protein coding genes
Non-mapped non-coding genes
c
Pons-associated locus at chromosome 12
ref SNPs
-log10 P-value
r2
Top lead SNP
Lead SNPs
Independent significant SNPs
←NUP37
←RP11-18O15.1
←IGF1
MYBPC1→
CHPT1→
←GNPTAB
←HSPE1P4
DRAM1→
←CCDC53
PARPBP→
←RP11-755O11.2
Y_RNA→
snoU13→
←RP11-512N21.3
RP11-554E23.4→
←PMCH
←SYCP3
RNA5SP369→
←RP11-554E23.2
←RN7SL793P
←RNU6-101P
←RNU6-1183P
←RP11-511H9.3
RP11-512N21.1→
RNA5SP368→
RP11-554E23.3→
←RNU6-172P
Mapped genes
Non-mapped protein coding genes
Non-mapped non-coding genes

d SCP-associated locus at chromosome 1

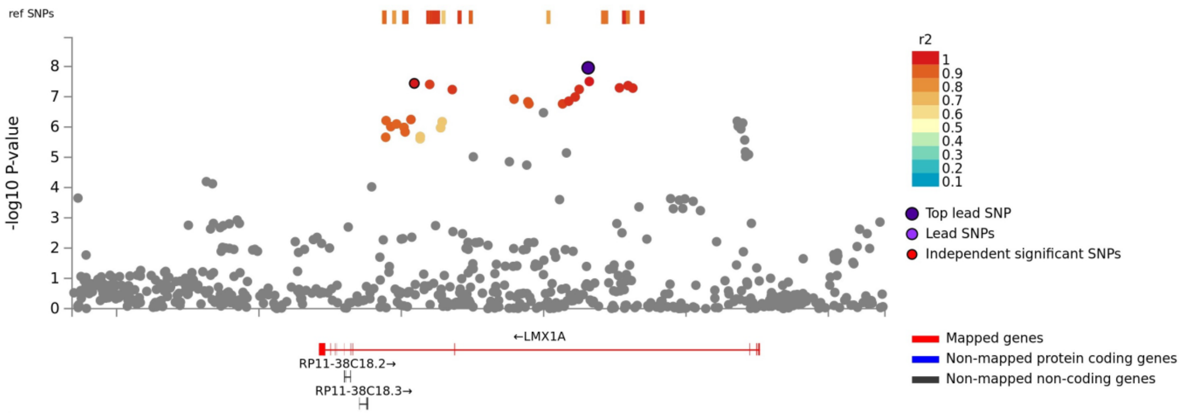

e Medulla oblongata-associated locus at chromosome 12

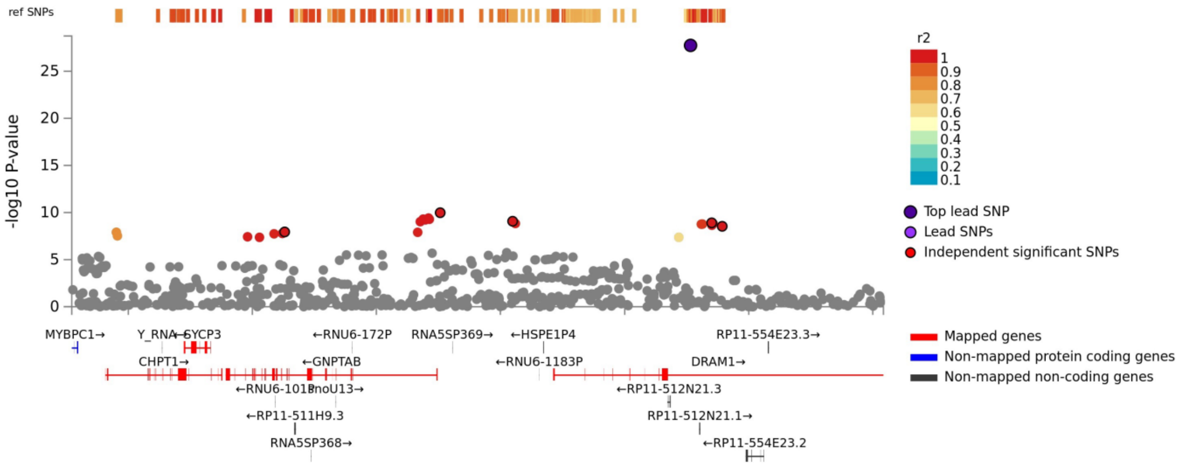

Supplementary Fig. 4 | Regional plots for the most significant genetic locus from the genome-wide association studies for volumes of the whole brainstem at chromosome 5 (**a**), midbrain at chromosome 7 (**b**), pons at chromosome 12 (**c**), superior cerebellar peduncle at chromosome 1 (**d**), and medulla oblongata at chromosome 12 (**e**). SCP; superior cerebellar peduncle. SNP; single-nucleotide polymorphism.

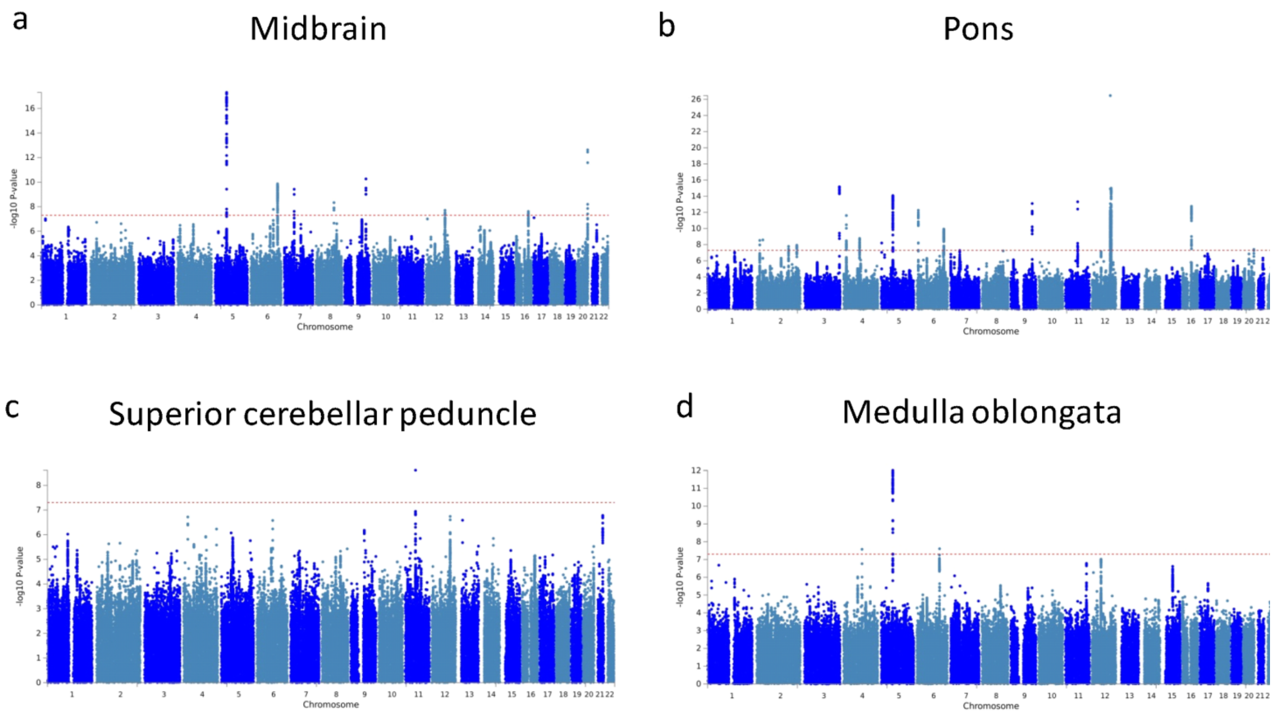

Supplementary Fig. 5 | Manhattan plots for volumes of the midbrain (**a**), pons (**b**), superior cerebellar peduncle (**c**), and medulla oblongata (**d**) from the genome-wide association studies when not accounting for whole brainstem volume. The red horizontal lines indicate genome-wide significance (two-sided P < 5e-8). Additional information for the significant loci is provided in Supplementary Table 6.

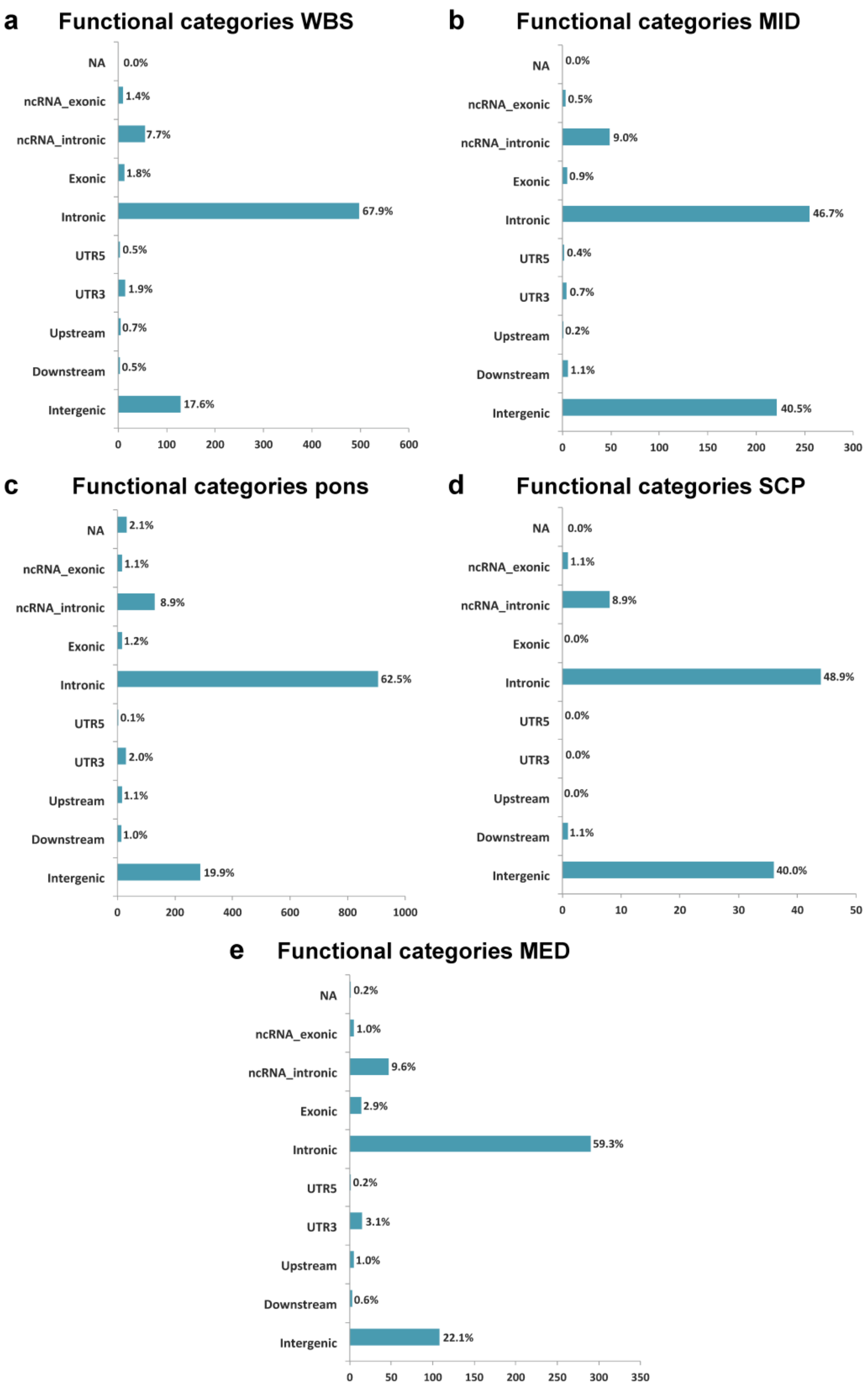

Supplementary Fig. 6 | Functional single-nucleotide polymorphism categories from the genome-wide association studies for volumes of the whole brainstem (**a**), midbrain (**b**), pons (**c**), superior cerebellar peduncle (**d**), and medulla oblongata (**e**). WBS; whole brainstem. MID; midbrain. SCP; superior cerebellar peduncle. MED; medulla oblongata. NA; not applicable. ncRNA; non-coding ribonucleic acid. UTR5; five prime untranslated region. UTR3; three prime untranslated region.

a Loci for whole brainstem at chromosome 12

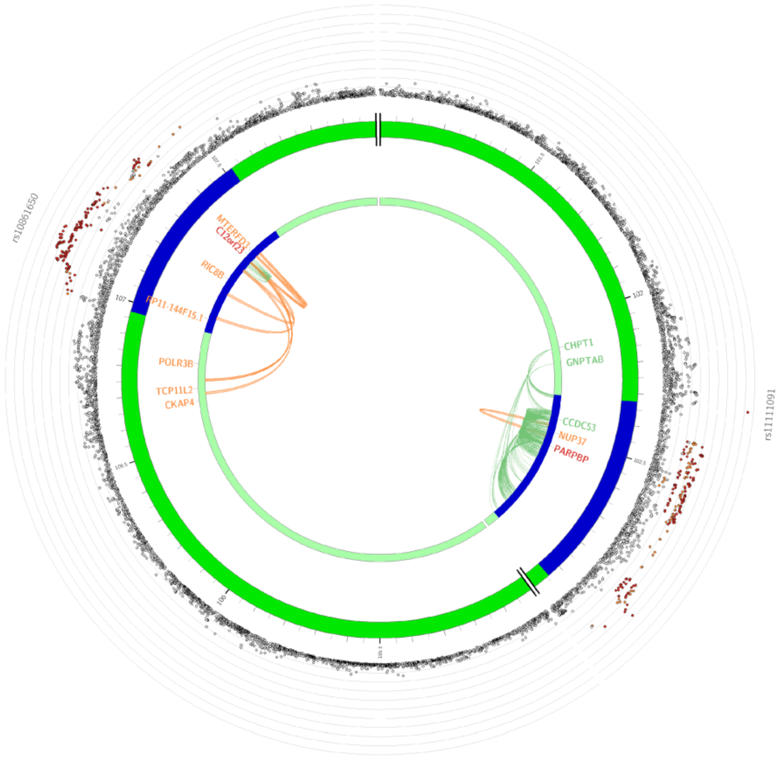

b Loci for midbrain at chromosome 14

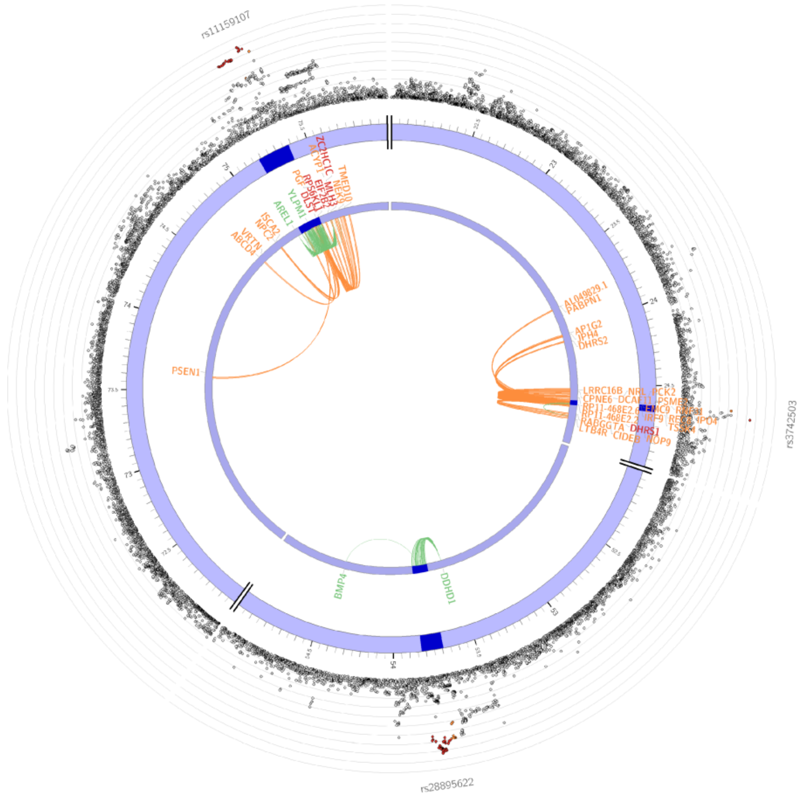

c **Loci for pons at chromosome 1**

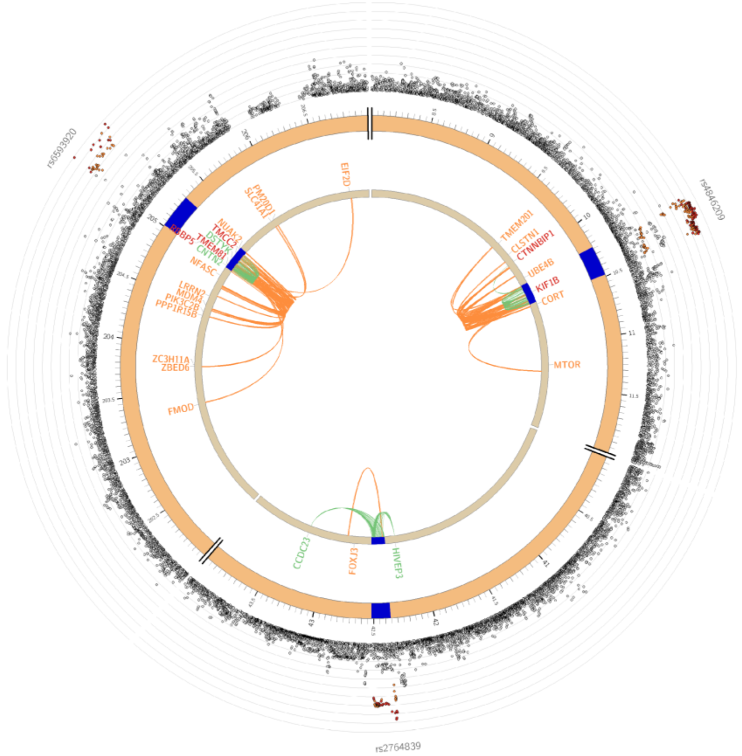

d **Loci for SCP at chromosome 4**

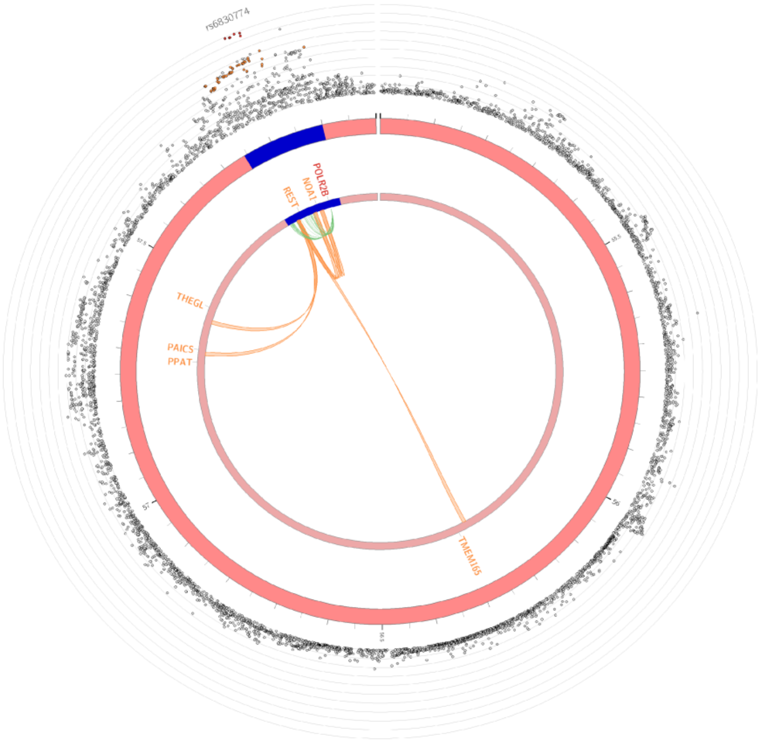

e **Loci for medulla oblongata at chromosome 4**

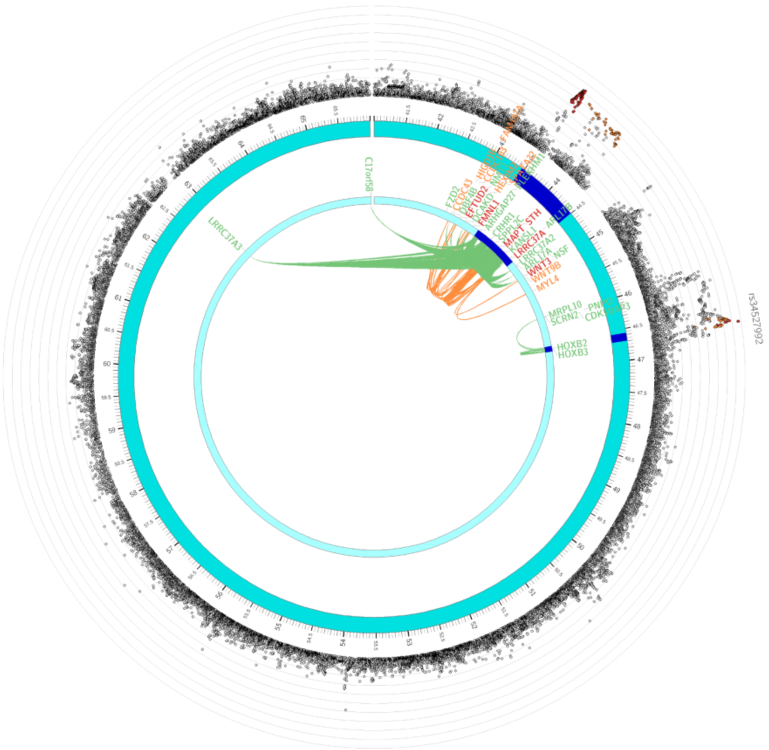

Supplementary Fig. 7 | Examples of Circos plots of mapped genes for the whole brainstem at chromosome 12 (**a**), midbrain at chromosome 14 (**b**), pons at chromosome 1 (**c**), superior cerebellar peduncle at chromosome 4 (**d**), and medulla oblongata at chromosome 4 (**e**). The plots show mapped genes of significant genetic loci at $P < 5e\text{-}8$ from the genome-wide association studies of brainstem volumes (blue regions). The genes were linked to the loci by eQTL mapping (green lines) and chromatin interactions (orange lines). Green color indicates genes implicated by eQTLs, orange color indicates genes mapped by chromatin interactions, and genes implicated by both strategies are in red color. The outer layers show the Manhattan plots of single nucleotide polymorphisms from the genome-wide association studies. The statistical analyses performed for the gene mapping in Functional Mapping and Annotation of GWAS (FUMA) are described in detail on https://fuma.ctglab.nl/tutorial. All parameters used for the present study are available at https://fuma.ctglab.nl/browse/; ID 97-105.

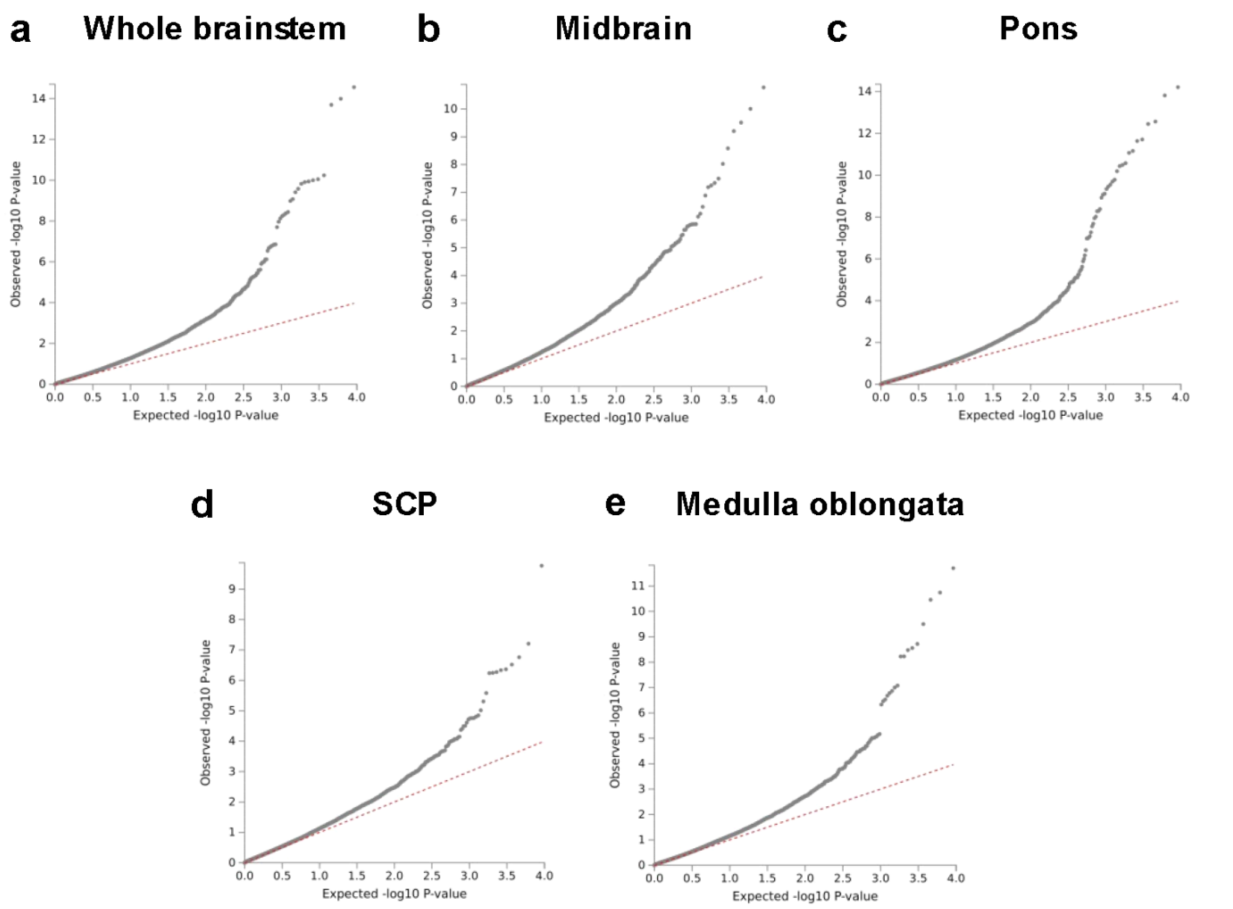

Supplementary Fig. 8 | Q-Q plots for volumes of the whole brainstem (**a**), midbrain (**b**), pons (**c**), superior cerebellar peduncle (**d**), and medulla oblongata (**e**) from the genome-wide gene-based association analyses. SCP; superior cerebellar peduncle.

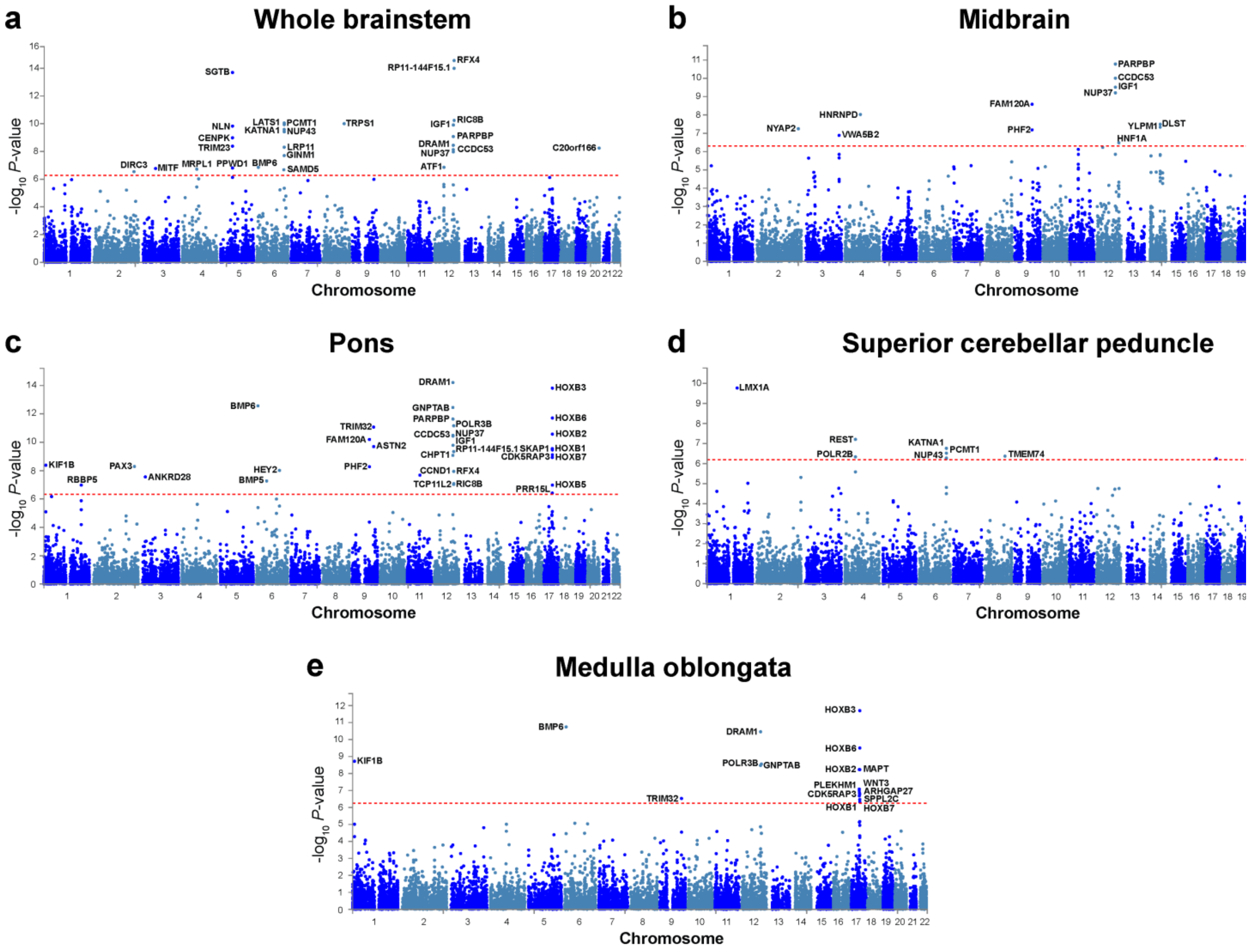

Supplementary Fig. 9 | Manhattan plots from the genome-wide gene-based association analyses for volumes of the whole brainstem (**a**), midbrain (**b**), pons (**c**), superior cerebellar peduncle (**d**), and medulla oblongata (**e**) in the discovery sample when adjusting for analyses of five volumes. Twenty-seven genes were associated with whole brainstem volume and 12, 33, 7, and 17 genes were associated with volumes of the midbrain, pons, superior cerebellar peduncle, and the medulla oblongata, respectively. Fifteen of the genes were only associated with whole brainstem volume, whereas 6, 12, 4, and 5 genes were only significant for midbrain, pons, SCP, and medulla oblongata volumes, respectively. The red horizontal lines indicate significance threshold of two-sided *P* < 5.4e-7 (i.e., 2.7e-6/5 volumes).

a

Genetic overlap between brainstem volumes and ADHD

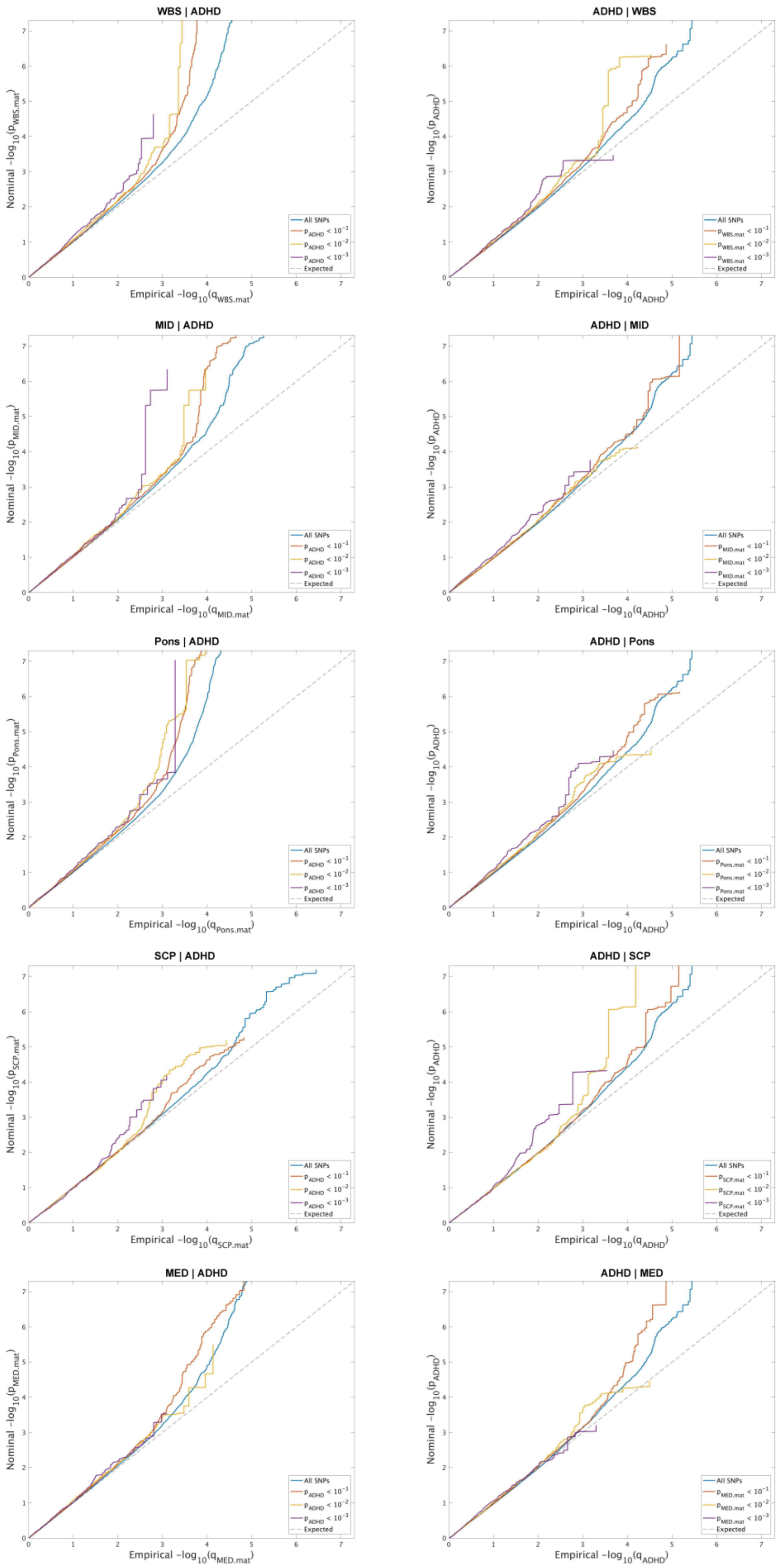

b Genetic overlap between brainstem volumes and ASD

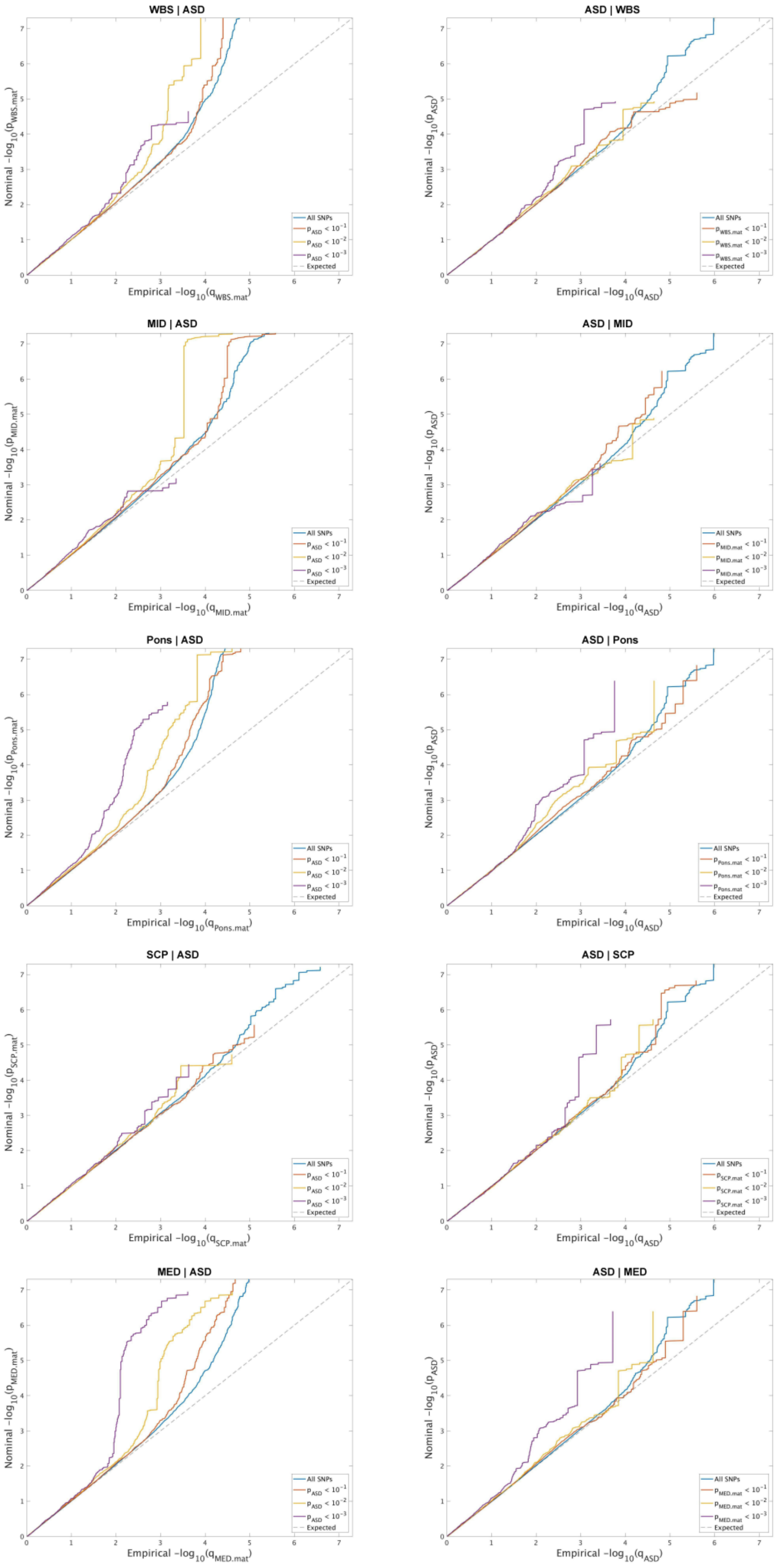

c Genetic overlap between brainstem volumes and BD

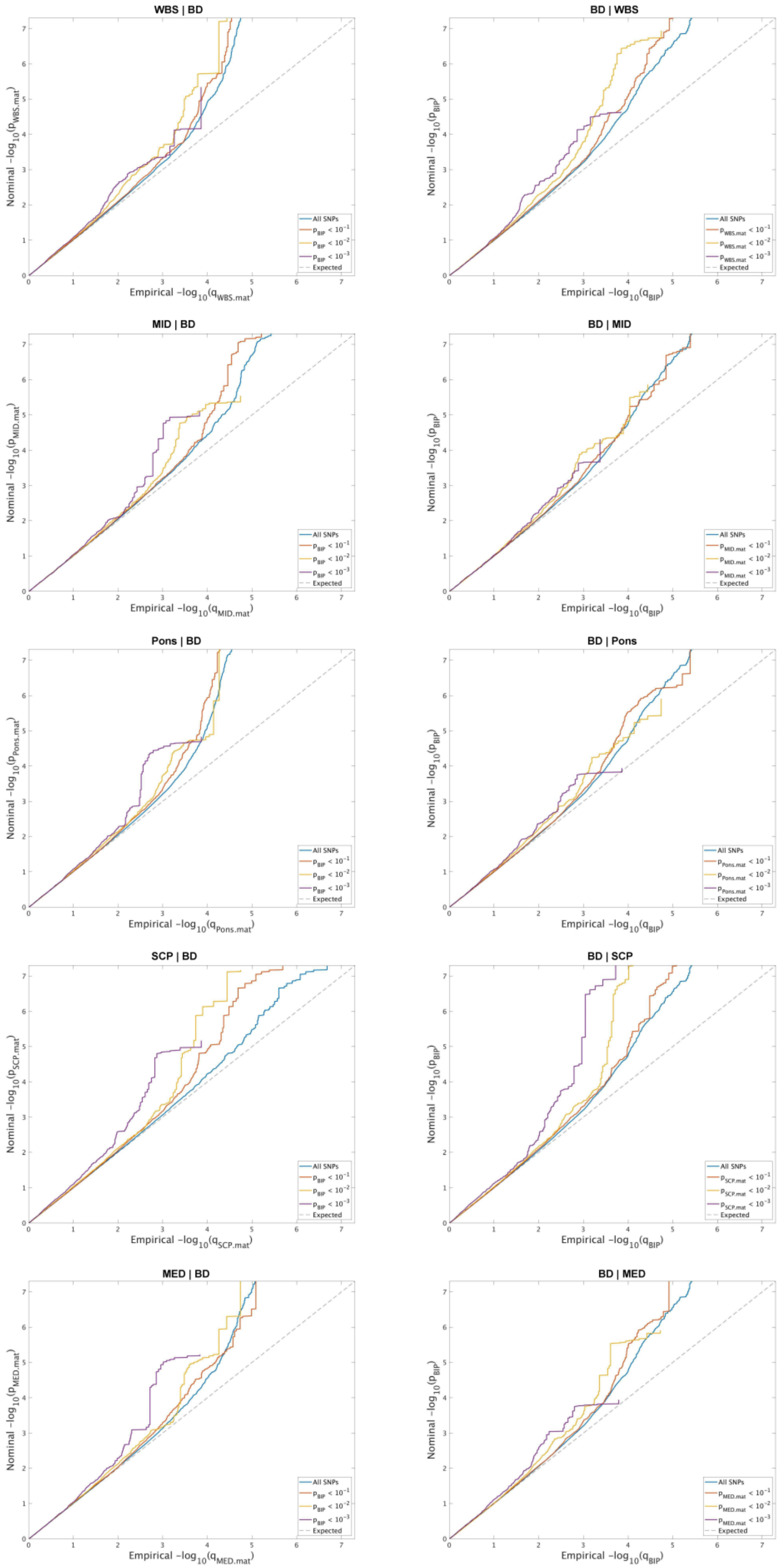

d

Genetic overlap between brainstem volumes and MD

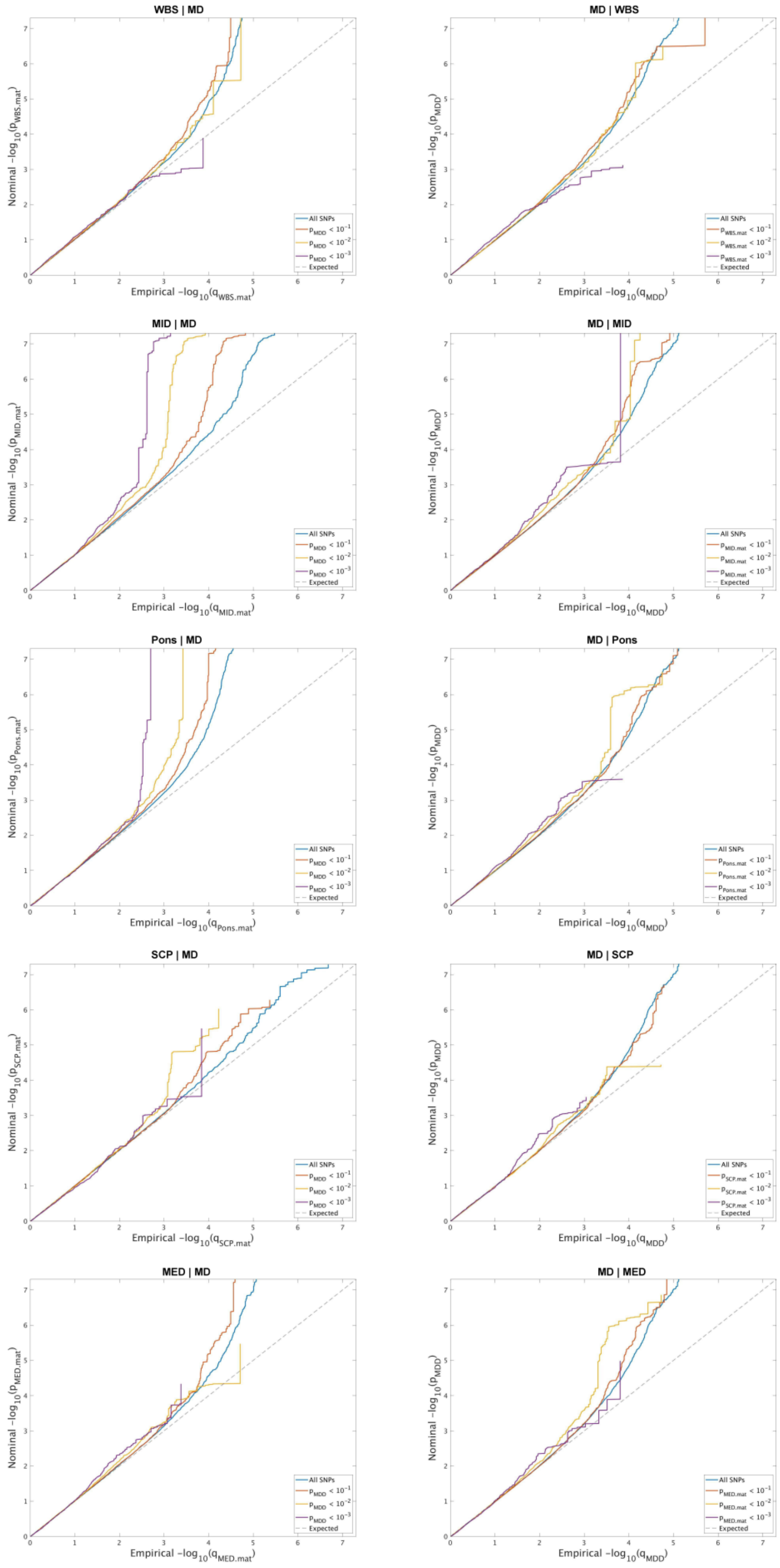

e **Genetic overlap between brainstem volumes and SCZ**

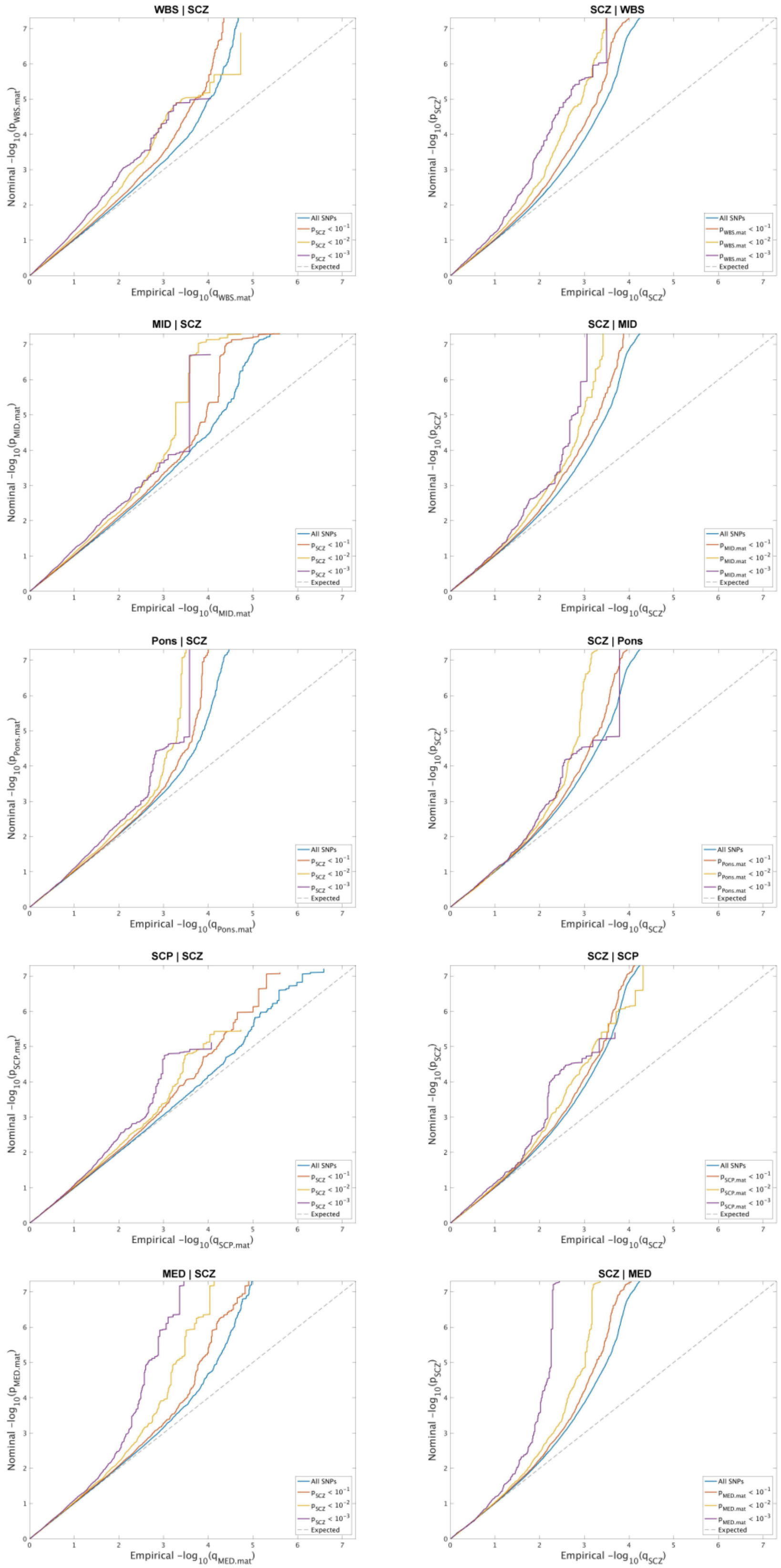

f

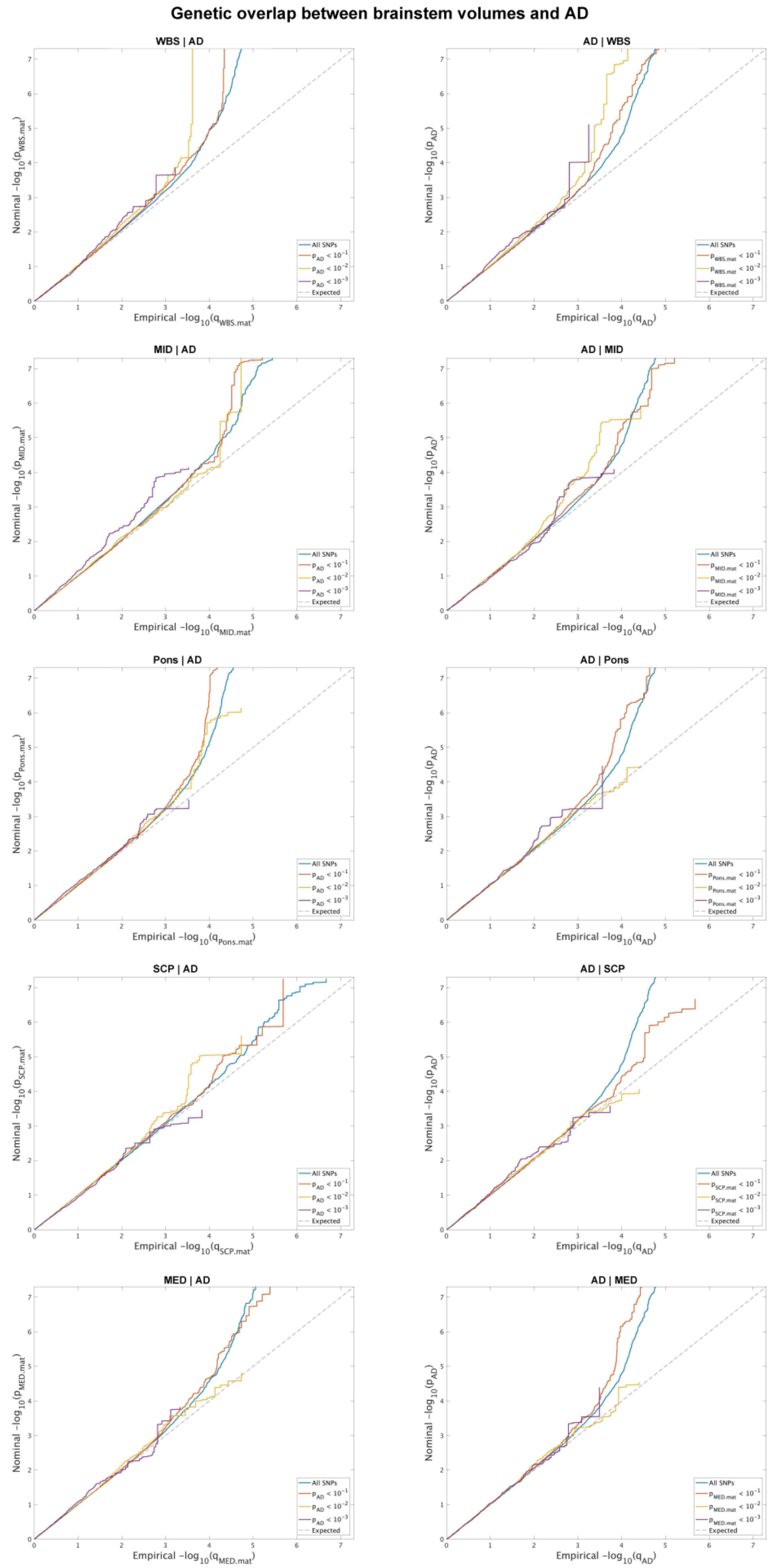
Genetic overlap between brainstem volumes and AD
WBS | AD
AD | WBS
MID | AD
AD | MID
Pons | AD
AD | Pons
SCP | AD
AD | SCP
MED | AD
AD | MED
All SNPs
Expected

g **Genetic overlap between brainstem volumes and MS**

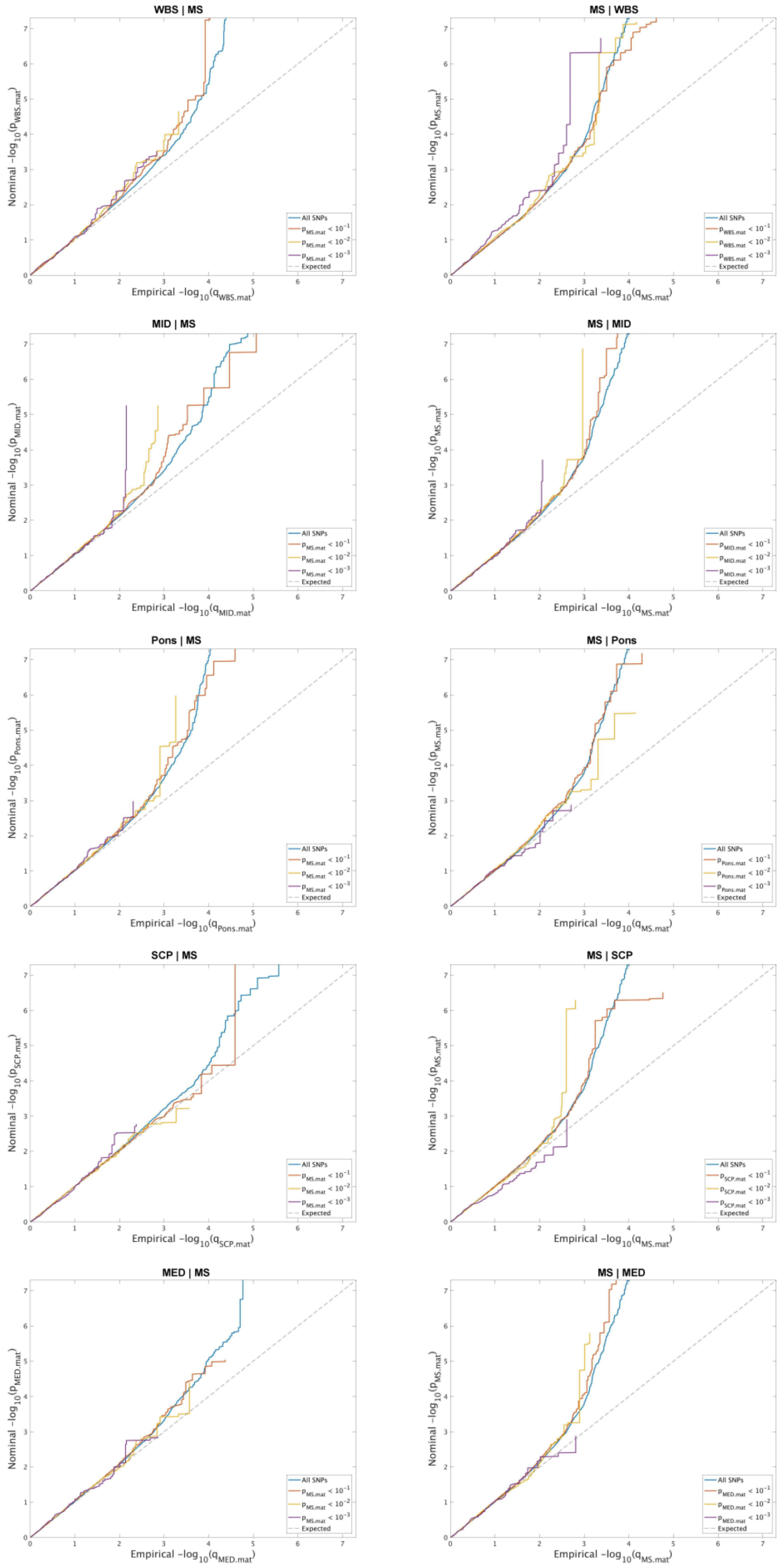

h Genetic overlap between brainstem volumes and PD

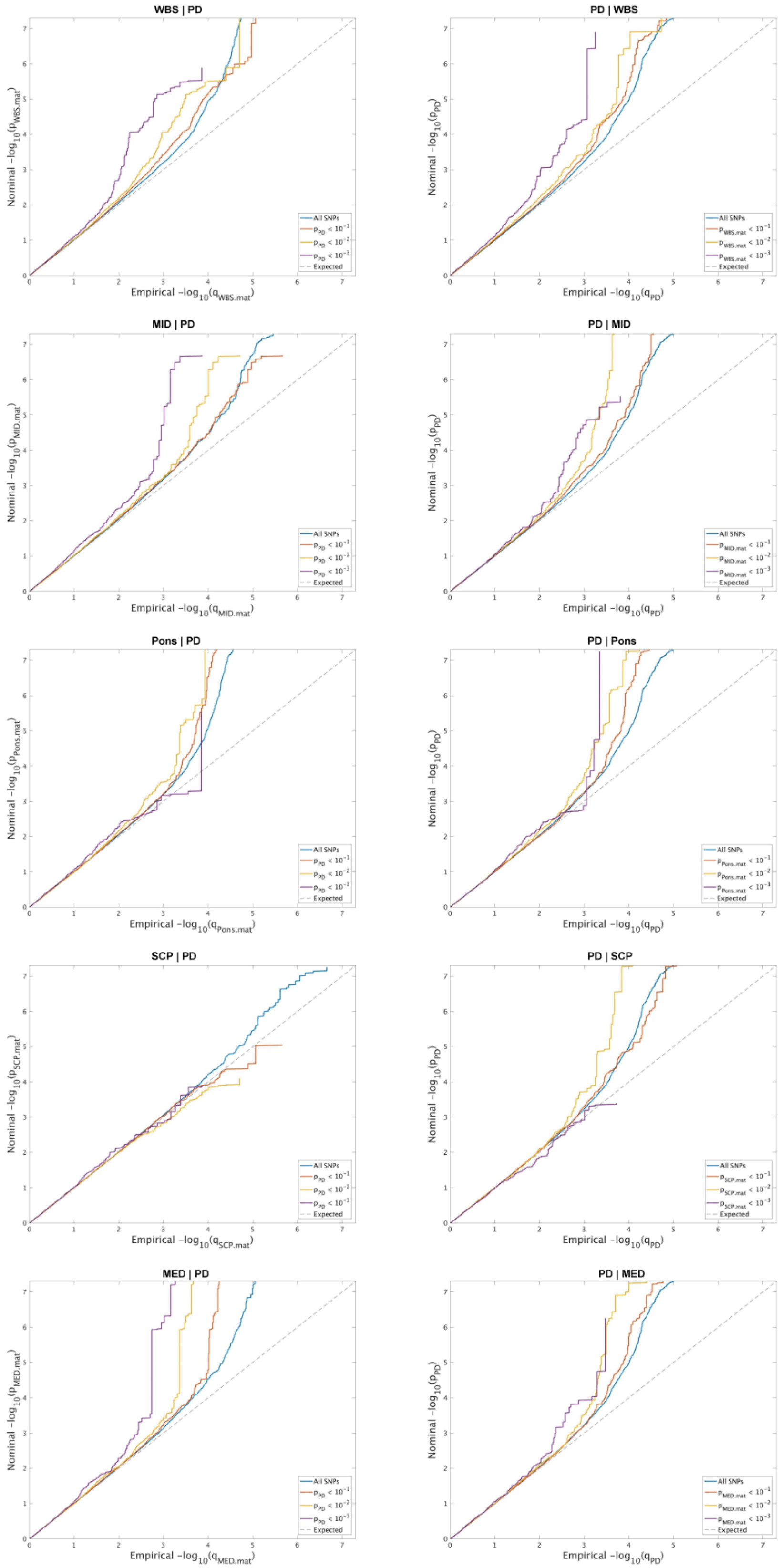

Supplementary Fig. 10 | Conditional Q-Q plots for brainstem volumes given associations with the disorder (left figures) and vice versa (right figures), for attention deficit hyperactivity disorder (**a**), autism spectrum disorder (**b**), bipolar disorder (**c**), major depression (**d**), schizophrenia (**e**), Alzheimer's disease (**f**), multiple sclerosis (**g**), and Parkinson's disease (**h**). ADHD; attention deficit hyperactivity disorder. ASD; autism spectrum disorders. BD; bipolar disorder. MD; major depression. SCZ; schizophrenia. AD; Alzheimer's disease. MS; multiple sclerosis. PD; Parkinson's disease. WBS; whole brainstem. MID; midbrain. SCP; superior cerebellar peduncle. MED; medulla oblongata.

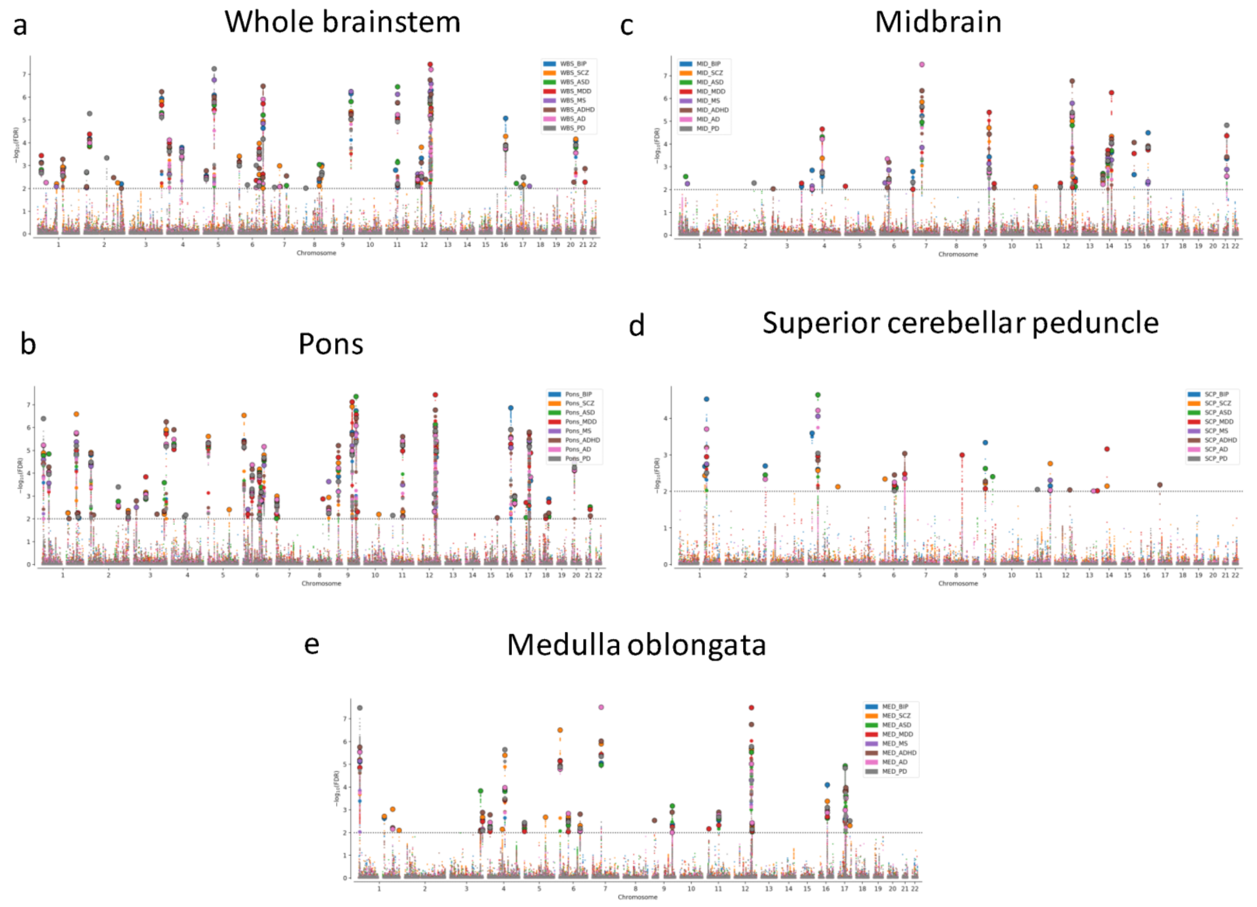

Supplementary Fig. 11 | Manhattan plots of genetic loci for each brainstem region identified by the condition false discovery rate analyses when conditioned on the eight brain disorders. These analyses revealed a total of 208 independent significant single-nucleotide polymorphisms (SNPs) for whole brainstem volume (**a**), 111 SNPs for midbrain volume (**b**), 270 SNPs for pons volume (**c**), 55 SNPs for superior cerebellar peduncle volume (**d**), and 125 SNPs for medulla oblongata volume (**e**). ASD; autism spectrum disorders. BD; bipolar disorder. MD; major depression. SCZ; schizophrenia. AD; Alzheimer's disease. MS; multiple sclerosis. PD; Parkinson's disease.

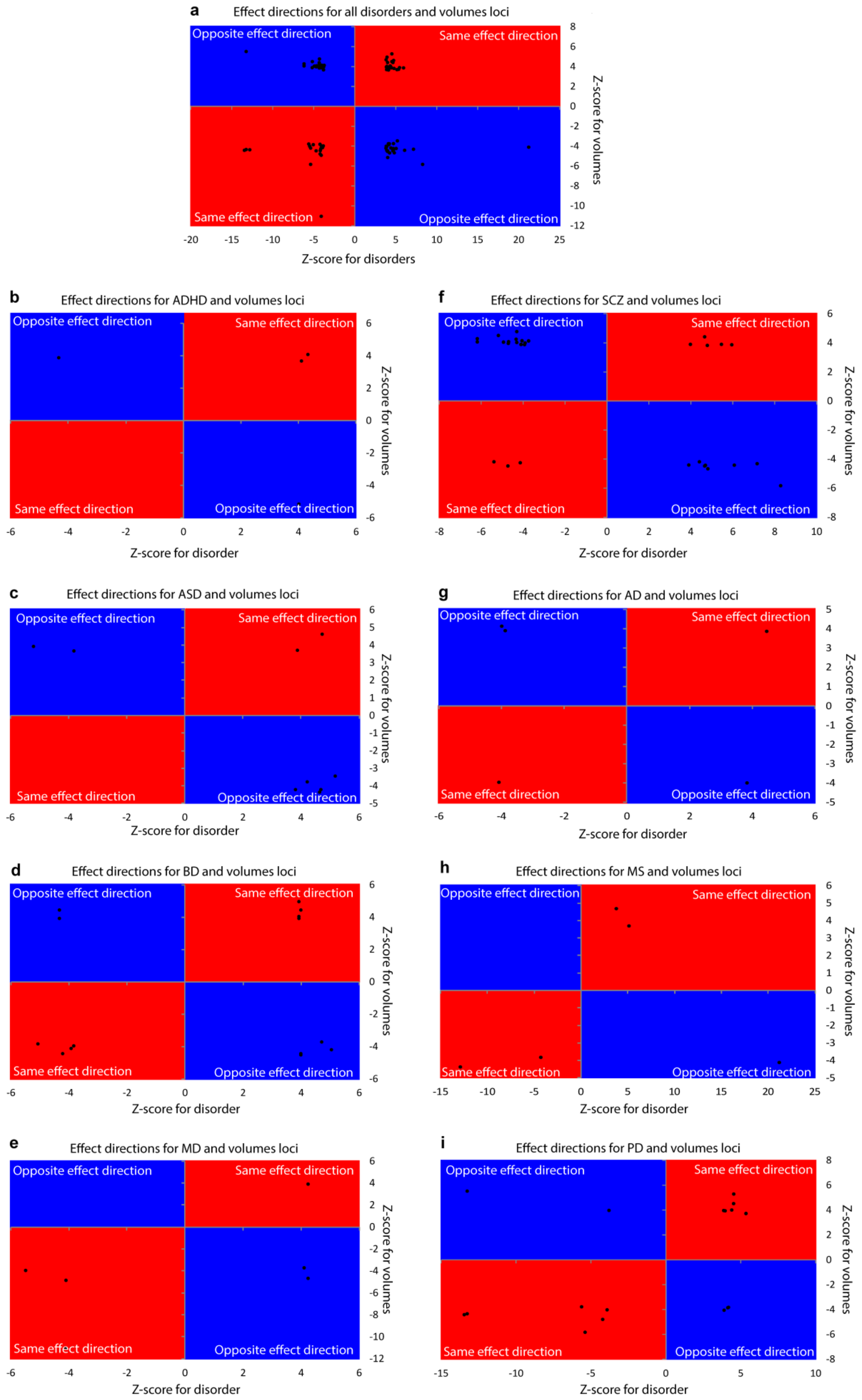
a
Effect directions for all disorders and volumes loci
Opposite effect direction
Same effect direction
Same effect direction
Opposite effect direction
Z-score for volumes
Z-score for disorders
b
Effect directions for ADHD and volumes loci
f
Effect directions for SCZ and volumes loci
c
Effect directions for ASD and volumes loci
g
Effect directions for AD and volumes loci
d
Effect directions for BD and volumes loci
h
Effect directions for MS and volumes loci
e
Effect directions for MD and volumes loci
i
Effect directions for PD and volumes loci
Z-score for disorder

Supplementary Fig. 12 | Effect directions for the lead single-nucleotide polymorphisms (SNPs) shared between the brainsteim volumes and the eight brain disorders. The plots indicate Z-scores for the lead SNPs across all volumes and (**a**) all disorders, (**b**) all volumes and ADHD, (**c**) all volumes and ASD, (**d**) all volumes and BD, (**e**) all volumes and MD, (**f**) all volumes and SCZ, (**g**) all volumes and AD, (**h**) all volumes and MS, and (**i**) all volumes and PD. Red color indicates same effect directions (i.e., disorder-linked variants were associated with increased volumes), whereas blue color indicates opposite effect directions (i.e., disorder-linked variants were associated with reduced volumes). ADHD; attention deficit hyperactivity disorder. ASD; autism spectrum disorders. BD; bipolar disorder. MD; major depression. SCZ; schizophrenia. AD; Alzheimer's disease. MS; multiple sclerosis. PD; Parkinson's disease.

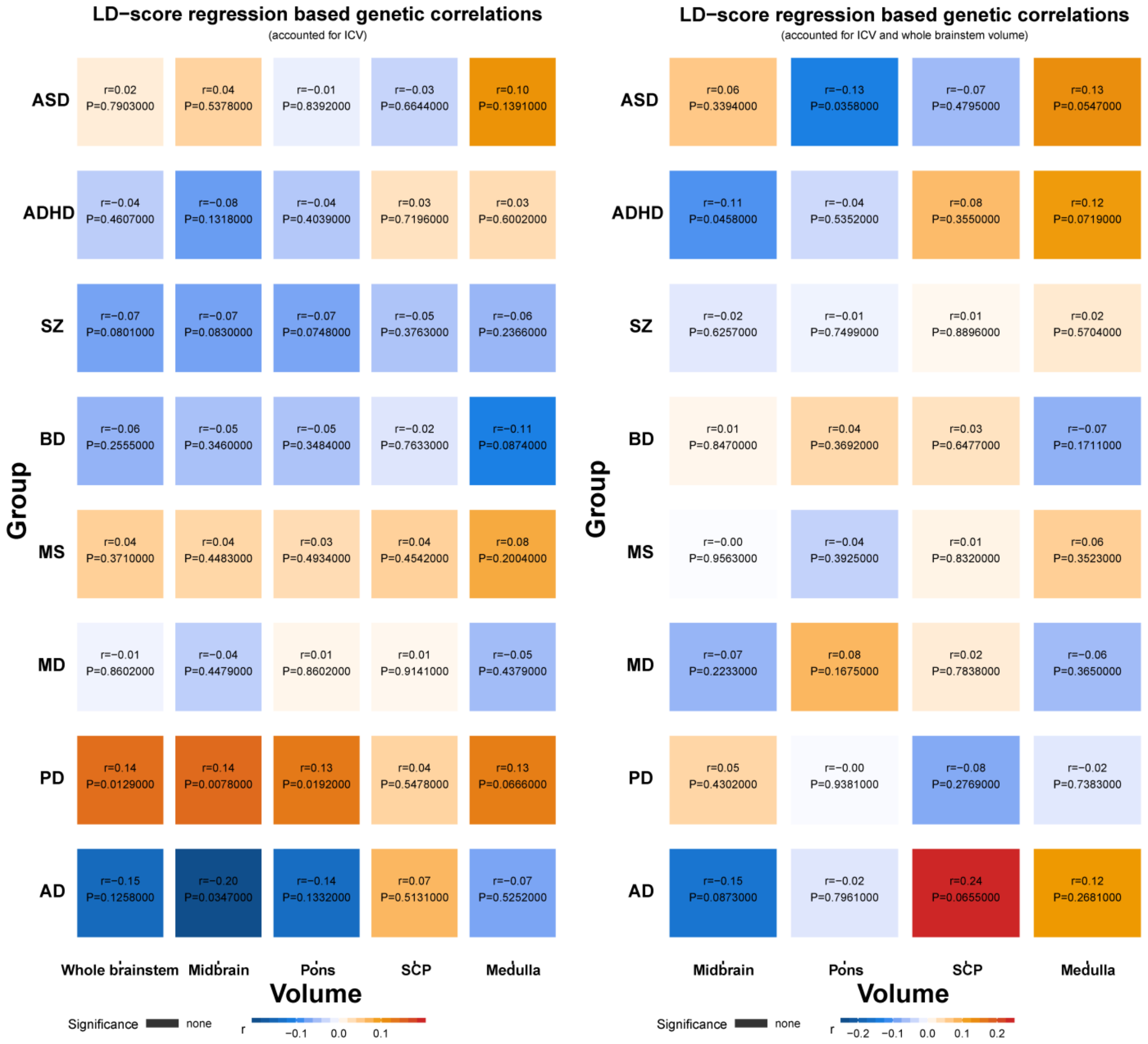

Supplementary Fig. 13 | LD-score regression based genetic correlations between brainstem volumes and eight brain disorders. There were correlations between brainstem volumes and ASD, ADHD, and PD with uncorrected $P < 0.05$, yet these were not significant after multiple testing corrections. ADHD; attention deficit hyperactivity disorder. ASD; autism spectrum disorder. BD; bipolar disorder. MD; major depression. MS; multiple sclerosis. PD; Parkinson's disease. SCZ; schizophrenia. ICV; intracranial volume. SCP; superior cerebellar peduncle. Medulla; medulla oblongata.

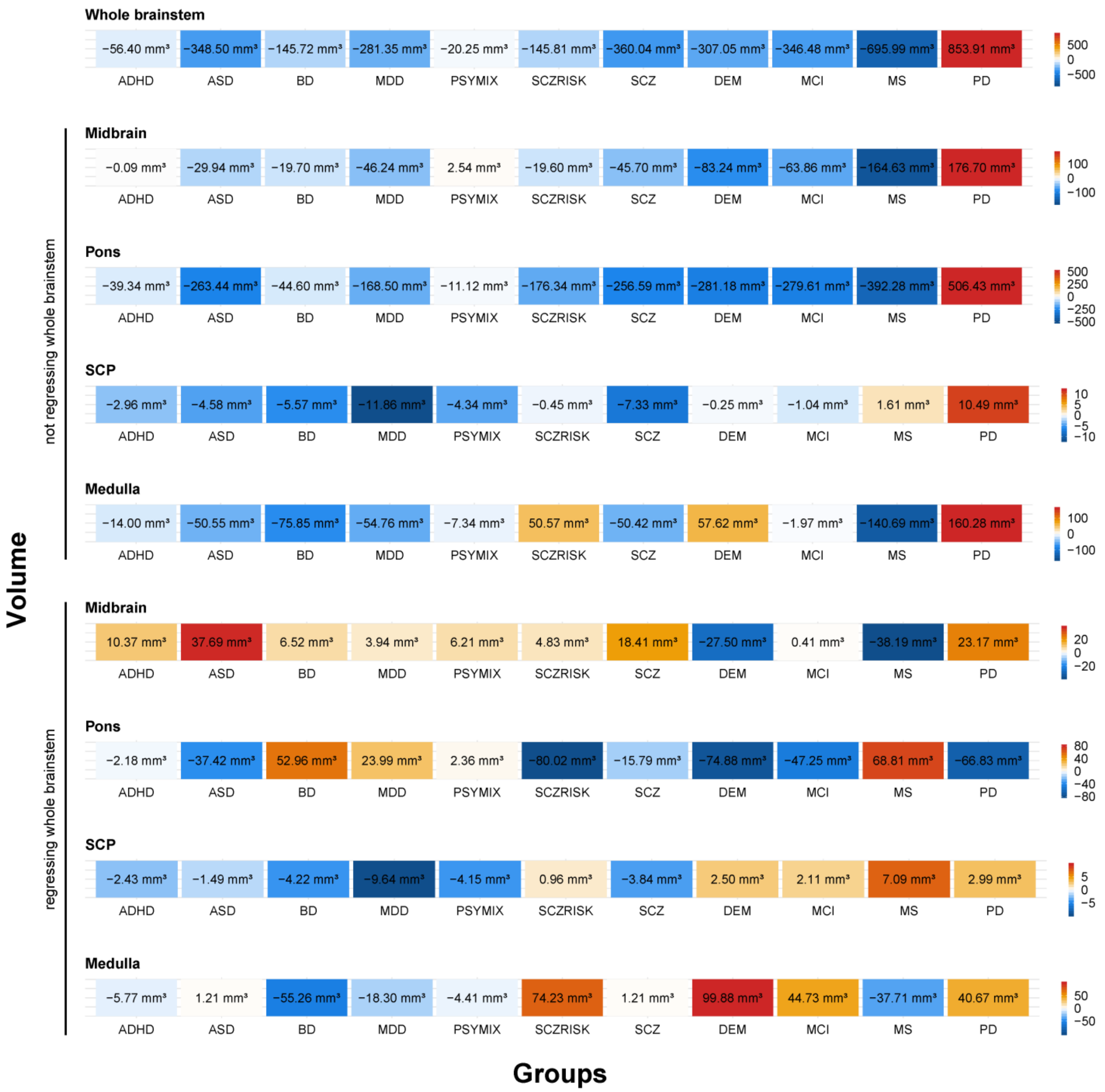

Supplementary Fig. 14 | Volumes of brainstem structures in individuals with common brain disorders compared to healthy controls. Linear models were run covarying for sex, age, age², intracranial volume, and scanner site. The analyses for volumes of midbrain, pons, SCP, and medulla oblongata were run both with and without covarying for whole brainstem volume. The figure depicts the resulting case-control differences in mm^3, whereas group differences in Cohen's d are shown in Fig. 5. ADHD; attention-deficit/hyperactivity disorder. ASD; autism spectrum disorders. BD; bipolar disorder. MDD; major depressive disorder. PSYMIX; non-SCZ psychosis spectrum diagnoses. SCZRISK; prodromal SCZ or at risk mental state. SCZ; schizophrenia. DEM; dementia. MCI; mild cognitive impairment. MS; multiple sclerosis. PD; Parkinson's disease. WBS; whole brainstem. SCP; superior cerebellar peduncle. Medulla; medulla oblongata. The color scale indicates volume group difference in mm^3.

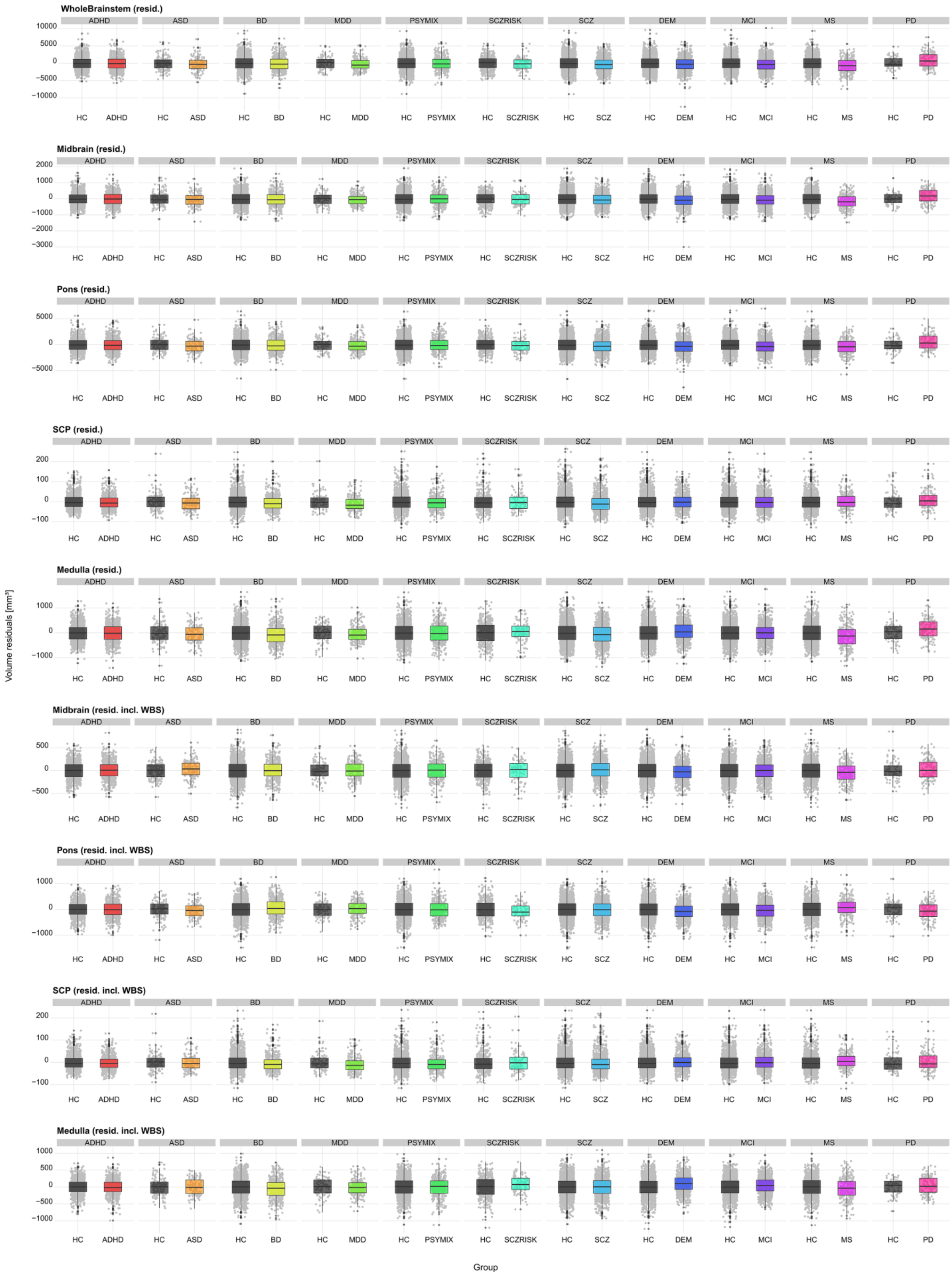
WholeBrainstem (resid.)
Midbrain (resid.)
Pons (resid.)
SCP (resid.)
Medulla (resid.)
Midbrain (resid. incl. WBS)
Pons (resid. incl. WBS)
SCP (resid. incl. WBS)
Medulla (resid. incl. WBS)
ADHD ASD BD MDD PSYMIX SCZRISK SCZ DEM MCI MS PD
HC
Volume residuals [mm³]
Group

Supplementary Fig. 15 | Scatter plots for volumes of brainstem structures in individuals with common brain disorders and healthy controls. Linear models were run covarying for sex, age, age^2, intracranial volume, and scanner site and residualized volumes are shown in the figure. The analyses for volumes of midbrain, pons, SCP, and medulla oblongata were run both with (as indicated by "incl. WBS" in the figure) and without covarying for whole brainstem volume. The clinical groups include the following numbers of participants with the diagnosis and controls: ADHD (*n* = 681 patients/*n* = 992 HC), ASD (*n* = 125/*n* = 140), BD (*n* = 464/*n* = 1,513), MDD (*n* = 211/*n* = 93), SCZ (*n* = 1,044/*n* = 2,079), prodromal SCZ or at risk mental state (SCZRISK; *n* = 91/*n* = 402), non-SCZ psychosis spectrum diagnoses (PSYMIX; *n* = 308/*n* = 1,430), dementia (*n* = 756/*n* = 1,921), MCI (*n* = 987/*n* = 1,655), MS (*n* = 257/*n* = 1,053), and PD (*n* = 138/*n* = 67). HC; healthy control. ADHD; attention-deficit/hyperactivity disorder. ASD; autism spectrum disorders. BD; bipolar disorder. MDD; major depressive disorder. PSYMIX; non-SCZ psychosis spectrum diagnoses. SCZRISK; prodromal SCZ or at risk mental state. SCZ; schizophrenia. DEM; dementia. MCI; mild cognitive impairment. MS; multiple sclerosis. PD; Parkinson's disease. WBS; whole brainstem. SCP; superior cerebellar peduncle. Medulla; medulla oblongata. Center line, median. Box limits, upper and lower quartiles. Whiskers, 1.5 interquartile range. Points, outliers.

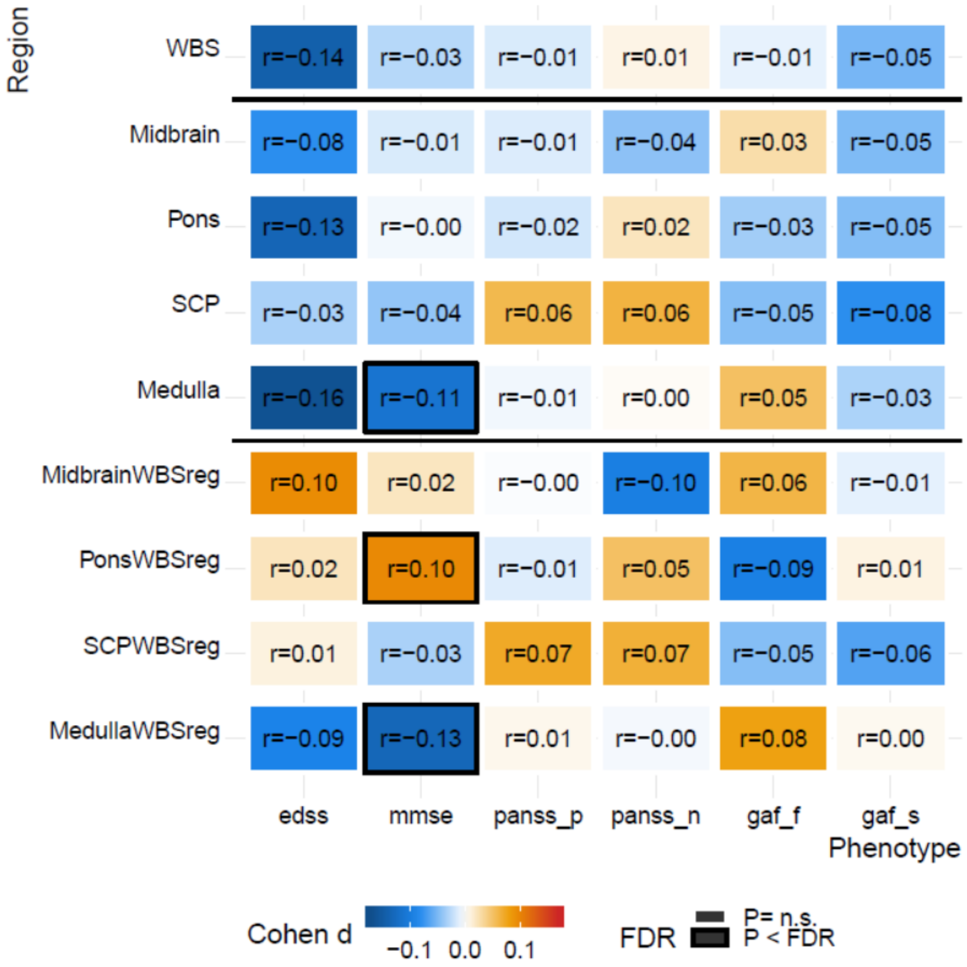

Supplementary Fig. 16 | Associations between brainstem volumes and clinical variables in MCI, DEM, MS, and SCZ. Across individuals with MCI and DEM (n = 1610), there were negative associations between MMSE scores and medulla oblongata volume before (r = -0.11, degrees of freedom (d.f.) = 1489, P = 1.8e-05) and after (r = -0.13, d.f. = 1488, P = 3.5e-07) accounting for whole brainstem volume. In addition, there was a significant positive association between MMSE and pons volume when adjusted for the whole brainstem (r = 0.10, d.f. = 1488, P = 1.7e-04). There were no significant associations between brainstem volumes and EDSS in the individuals with MS (all P > 0.05). In SCZ, there was no significant association between brainstem volumes and symptom or function scores of the GAF or positive and negative scores of the PANSS (all P > 0.05). EDSS; Expanded Disability Status Scale. GAF; Global Assessment of Functioning scale. MMSE; Mini-Mental State Examination. PANSS; Positive and Negative Syndrome Scale. MCI; mild cognitive impairment. DEM; dementia. MS; multiple sclerosis. SCZ; schizophrenia. PD; Parkinson's disease. P; positive. N; negative. F; functioning. S; symptoms. FDR; false discovery rate.

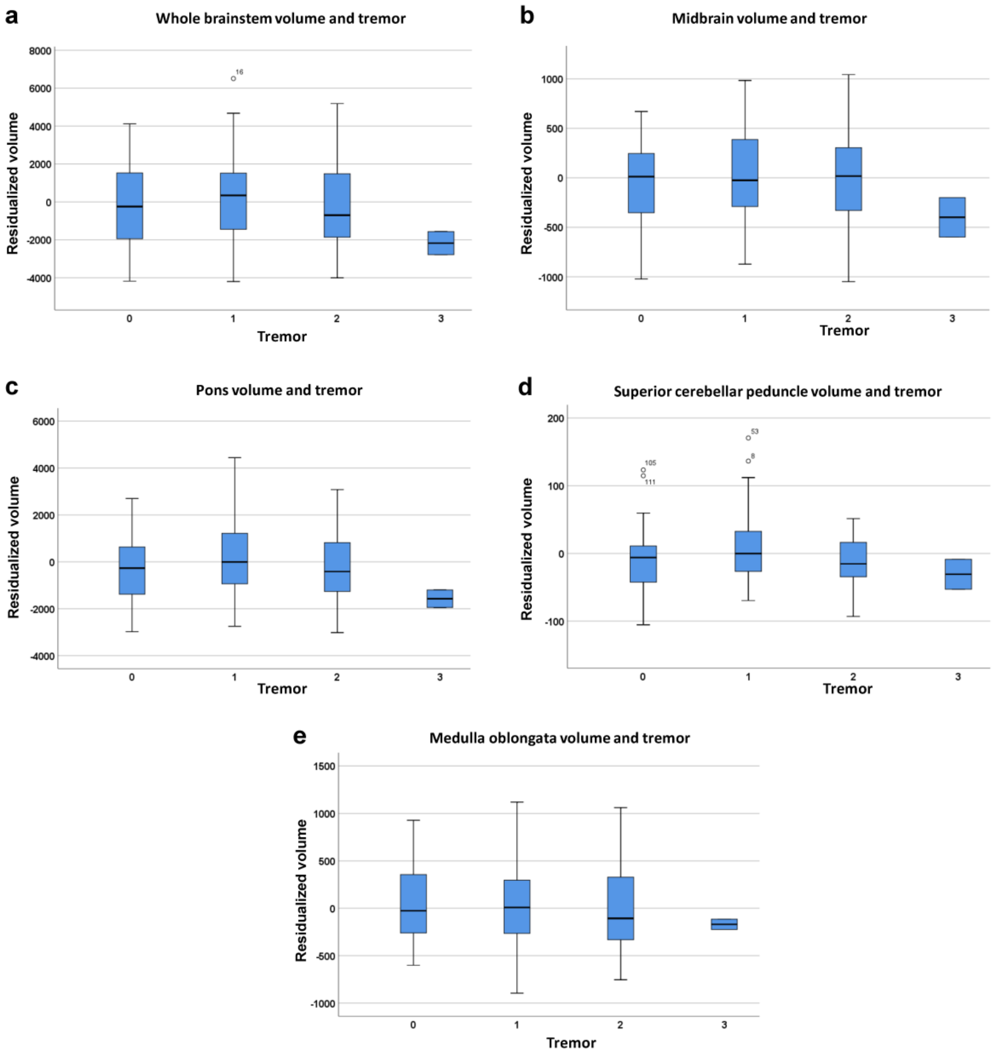

Supplementary Fig. 17 | Tremor and brainstem volumes in individuals with Parkinson's disease (PD; n = 128).We examined whether the volume increases in the individuals with PD were related to tremor, which could cause increased within-scanner motion and confound the brainstem segmentation. Here, we used item 2.10 of the Unified Parkinson's Disease Rating Scale III: "Over the past week, have you usually had shaking or tremor? 0: Normal: Not at all. I have no shaking or tremor; 1: Slight: Shaking or tremor occurs but does not cause problems with any activities.; 2: Mild: Shaking or tremor causes problems with only a few activities; 3: Moderate: Shaking or tremor causes problems with many of my daily activities; and 4: Severe: Shaking or tremor causes problems with most or all activities." n = 30 individuals had a tremor score of 0, n = 74 individuals had a score of 1, n = 22 individuals had a score of 2, n = 2 individuals had a score of 3, and n = 0 had a score of 4. We then grouped the individuals according to the tremor level and compared brainstem volumes between these groups using a two-sided linear model, covarying for gender, intracranial volume, scanner, age, and age^2. These analyses were not adjusted for multiple comparisons. There were no significant effects of tremor group on brainstem volumes (whole brainstem: $F(3,118)$ = 0.98, P = 0.41; midbrain: $F(3,118)$ = 0.73, P = 0.54; pons: $F(3,118)$ = 1.23, P = 0.30; superior cerebellar peduncle: $F(3,118)$ = 1.91, P = 0.13; and medulla oblongata:

$F(3,118) = 0.24$, $P = 0.87$). The graphs depict box-and-whisker plots. Center line, median. Box limits, upper and lower quartiles. Whiskers, 1.5 interquartile range. Points, outliers.

Supplementary Note 1

Supplementary clinical analyses

Additional clinical analyses were run for individuals with multiple sclerosis (MS) or Parkinson's disease (PD) using linear models. All brain magnetic resonance imaging data from the individuals with MS were examined by two neuroradiologists and then divided into two groups according to presence of infratentorial lesions. There was no significant difference in brainstem volumes between patients with (n = 153) and without (n = 91) infratentorial lesions (all $P > 0.05$). Patients without lesions had reduced volumes relative to the controls of the whole brainstem (Cohen's d = -0.23, degrees of freedom (d.f.) = 1149, P = 0.03), midbrain (Cohen's d = - 0.26, d.f. = 1149, P = 0.01), and medulla oblongata (Cohen's d = -0.22, d.f. = 1149, P = 0.03). There were significant reductions in volumes of patients with infratentorial lesions relative to controls for the whole brainstem (Cohen's d = - 0.30, d.f. = 1211, P = 3.4e-04), the midbrain (Cohen's d = -0.36, d.f. = 1211, P = 1.9e-05), the pons (Cohen's d = -0.24, d.f. = 1211, P = 3.9e-03), and medulla oblongata (Cohen's d = - 0.29, d.f. = 1211, P = 4.9e-04). Across individuals with MS, there were no significant associations between brainstem volumes and Expanded Disability Status Scale (EDSS; all $P > 0.05$). However, in the individuals with infratentorial lesions, there were negative associations between EDSS and volumes of the whole brainstem (r = - 0.21, d.f. = 101, P = 0.03), pons (r = - 0.20, d.f. = 101, P = 0.045), and medulla oblongata (r = - 0.24, d.f. = 101, P = 0.01). There were no significant association between EDSS and volumes of the brainstem in the individuals without infratentorial lesions (all $P > 0.41$). There were no significant relationships between brainstem volumes and the Movement Disorder Society-sponsored revision of the Unified Parkinson's Disease Rating Scale III sum score or the Hoehn and Yahr Stage score (all $P > 0.05$) in the individuals with PD.

Supplementary Tables

Supplementary Table 1. Summary of included samples.

Sample	Source	Comment	Reference
ABIDE1	http://fcon_1000.projects.nitrc.org/	Primary support for the work by Adriana Di Martino was provided by the NIMH (K23MH087770) and the Leon Levy Foundation. Primary support for the work by Michael P. Milham and the INDI team was provided by gifts from Joseph P. Healy and the Stavros Niarchos Foundation to the Child Mind Institute, as well as by an NIMH award to MPM (R03MH096321).	1
ABIDE2	http://fcon_1000.projects.nitrc.org/	Primary support for the work by Adriana Di Martino and her team was provided by the National Institute of Mental Health (NIMH 5R21MH107045). Primary support for the work by Michael P. Milham and his team provided by the National Institute of Mental Health (NIMH 5R21MH107045); Nathan S. Kline Institute of Psychiatric Research). Additional Support was provided by gifts from Joseph P. Healey, Phyllis Green and Randolph Cowen to the Child Mind Institute.	2
ABM	Authors	ABM was supported by the Research Council of Norway (grant number 229135) and Health South East Research Funding Agency (grant number 2015052).	3
ADDNEUROMED	Authors	AddNeuroMed consortium was led by Simon Lovestone, Bruno Vellas, Patrizia Mecocci, Magda Tsolaki, Iwona Kłoszewska, Hilkka Soininen. Their work was supported by InnoMed (Innovative Medicines in Europe), an integrated project funded by the European Union of the Sixth Framework program priority (FP6-2004- LIFESCIHEALTH-5).	4,5
ADHD200	http://fcon_1000.projects.nitrc.org/	F. Xavier Castellanos, David Kennedy, Michael Milham, and Stewart Mostofsky are responsible for the initial conception of the ADHD-200 Consortium. Consortium steering committee includes Jan Buitelaar, F. Xavier Castellanos, Dan Dickstein, Damien Fair, David Kennedy, Beatriz Luna, Michael Milham (Project Coordinator), Stewart Mostofsky, and Julie Schweitzer. Data aggregation and organization was coordinated by the INDI team, which included Saroja Bangaru, David Gutman, Maarten Mennes, and Michael Milham. Web infrastructure and data storage were coordinated by Robert Buccigrossi, Albert Crowley, Christian Hasselgrove, David Kennedy, Kimberly Pohland, and Nina Preuss. The ADHD-200 Global Competition Coordinators were Damien Fair (Chair of Selection Committee, Editor in Chief for Global Competition Special issue) and Michael Milham	6,7
ADHDWUE	Authors	Primary support for the study was provided by the German Research Foundation, grant number DFG KFO 125/2.	8,9
ADNI1<br>ADNI2	http://adni.loni.usc.edu/<br>http://adni.loni.usc.edu/	Data collection and sharing for this project was funded by the Alzheimer's Disease Neuroimaging Initiative (ADNI) (National Institutes of Health Grant U01 AG024904) and DOD ADNI (Department of Defense award number W81XWH-12-2-0012). ADNI is funded by the National Institute on Aging, the National Institute of Biomedical Imaging and Bioengineering, and through generous contributions from the following: AbbVie, Alzheimer’s Association; Alzheimer’s Drug Discovery Foundation; Araclon Biotech; BioClinica, Inc.; Biogen; Bristol-Myers Squibb Company; CereSpir, Inc.; Cogstate; Eisai Inc.; Elan Pharmaceuticals, Inc.; Eli Lilly and Company; EuroImmun; F. Hoffmann-La Roche Ltd and its affiliated company Genentech, Inc.; Fujirebio; GE Healthcare; IXICO Ltd.; Janssen	10,11

		Alzheimer Immunotherapy Research & Development, LLC.; Johnson & Johnson Pharmaceutical Research & Development LLC.; Lumosity; Lundbeck; Merck & Co., Inc.; Meso Scale Diagnostics, LLC.; NeuroRx Research; Neurotrack Technologies; Novartis Pharmaceuticals Corporation; Pfizer Inc.; Piramal Imaging; Servier; Takeda Pharmaceutical Company; and Transition Therapeutics. The Canadian Institutes of Health Research is providing funds to support ADNI clinical sites in Canada. Private sector contributions are facilitated by the Foundation for the National Institutes of Health (www.fnih.org). The grantee organization is the Northern California Institute for Research and Education, and the study is coordinated by the Alzheimer's Therapeutic Research Institute at the University of Southern California. ADNI data are disseminated by the Laboratory for Neuro Imaging at the University of Southern California.	
CIMH	Authors	CIMH was supported by the Deutsche Forschungsgesellschaft (DFG, projects ZI1253/3-1, ZI1253/3-2, KI 576/14-2, ME 1591/6-2) and the European Community's Seventh Framework Programme (FP7/2007–2013) grant agreement #602450 (IMAGEMEND).	12,13
DS000030 (CNP)	https://openfmri.org/	DS* data sets were obtained from the OpenfMRI database. *DS000030* work was supported by the Consortium for Neuropsychiatric Phenomics (NIH Roadmap for Medical Research grants UL1-DE019580, RL1MH083268, RL1MH083269, RL1DA024853, RL1MH083270, RL1LM009833, PL1MH083271, and PL1NS062410). *DS000171*: Trisha Patrician and Natalie Stroupe assisted with screening of participants. Allan Schmitt and Franklin Hunsinger collected the MR data.	14,15
DS000171	https://openfmri.org/		16
HUBIN	Authors	This study was supported by the Swedish Research Council (2006-2992, 2006-986, K2007-62X-15077-04-1, 2008-2167, K2008-62P-20597-01-3. K2010-62X-15078-07-2, K2012-61X-15078-09-3, 2017-00949), the regional agreement on medical training and clinical research between Stockholm County Council and the Karolinska Institutet, the Knut and Alice Wallenberg Foundation, and the HUBIN project.	17
KASP	Authors	KaSP was supported by grants from the Swedish Medical Research Council (SE: 2009-7053; 2013-2838; SC: 523-2014-3467), the Swedish Brain Foundation, Åhlén-siftelsen, Svenska Läkaresällskapet, Petrus och Augusta Hedlunds Stiftelse, Torsten Söderbergs Stiftelse, the AstraZeneca-Karolinska Institutet Joint Research Program in Translational Science, Söderbergs Königska Stiftelse, Professor Bror Gadelius Minne, Knut och Alice Wallenbergs stiftelse, Stockholm County Council (ALF and PPG), Centre for Psychiatry Research, KID-funding from the Karolinska Institutet.	18,19
MALTOSLO	Authors	The study was funded by the South-Eastern Norway Regional Health Authority (2015-2015078), Oslo University Hospital, a research grant from Mrs. Throne-Holst, and the Ebbe Frøland foundation.	20,21
NIMAGE	Authors	This project was supported by grants from National Institutes of Health (grant R01MH62873 to SV Faraone) for initial sample recruitment, and from NWO Large Investment (grant 1750102007010 to JK Buitelaar), NWO Brain & Cognition (grant 433-09-242 to JK Buitelaar), ZonMW Grant 60-60600-97-193, and grants from Radboud University Medical Center, University Medical Center Groningen, Accare, and VU University Amsterdam for subsequent assessment waves. NeuroIMAGE also receives funding from the European Community's Seventh Framework Programme (FP7/2007 – 2013) under grant agreements n° 602805 (Aggressotype), n°	22

		278948 (TACTICS), and n° 602450 (IMAGEMEND), and from the European Community's Horizon 2020 Programme (H2020/2014 – 2020) under grant agreements n° 643051 (MiND) and n° 667302 (CoCA).	
NORCOG	Authors	The Norwegian register of persons assessed for cognitive symptoms (NorCog) includes clinical and biological data from memory clinics in Norway (https://www.aldringoghelse.no-/norkog/). The register is owned by Oslo University Hospital and administered by Norwegian National Advisory Unit on Ageing and Health. The NORCOG sample includes individuals with mild cognitive impairment and dementia.	23
OASIS	http://www.oasis-brains.org/	The study was supported by grants P50 AG05681, P01 AG03991, R01 AG021910, P50 MH071616, U24 RR021382, R01 MH56584.	24,25
PPMI	http://www.ppmi-info.org/	Parkinson's disease progression markers initiative (PPMI) is an observational clinical study to verify progression markers in PD. The study includes a comprehensive set of clinical, imaging (including structural MRI) and biosample data. The study is sponsored by the Michael J. Fox foundation for Parkinson's Research and is made possible by restricted donations to the Foundation from a consortium of Parkinson's drug development stakeholders. PPMI is led by Principal Investigator Ken Marek, MD, President and Senior Scientist of the Institute for Neurodegenerative Disease in New Haven, Connecticut. Funding partners include abbvie, Allergan, Avid, Biogen, BioLegend, Bristol-Myers Squibb, Celgene, Denali, GE Healthcare, Genentech, GlaxoSmithKline, Lilly, Lundbeck, Merck, Meso Scale Discovery, Pfizer, Piramal, Prevail Therapeutics, Roche, Sanofi Genzyme, Servier, Takeda, Teva, ucb, verily, Voyager Therapeutics, and Golub Capital.	26
RSI-MS	Authors	Data collection in this MS cohort was supported by the South-Eastern Norway Regional Health Authority project 39569, Research Council of Norway grant 240102 and 240102, Oslo MS Society, Odd Fellow's Society for MS research. Healthy controls were sampled from the TOP study (same scanner).	27
SCHIZCONNECT1<br>SCHIZCONNECT2	http://schizconnect.org/<br>http://schizconnect.org/	Data used in preparation of this article were obtained from the SchizConnect (http://schizconnect.org) database. As such, the investigators within SchizConnect contributed to the design and implementation of SchizConnect and/or provided data but did not participate in analysis or writing of this report. Data collection and sharing for this project was funded by NIMH cooperative agreement 1U01 MH097435<br>*SCHIZCONNECT1* comprised BrainGluSchi, COBRE and MCIC samples (COINS). *SCHIZCONNECT2* comprised NUSDAST and NUNDA samples.<br>The respective samples were supported by the following grants: *BrainGluSchi*: NIMH R01MH084898-01A1. *COBRE*: 5P20RR021938 /P20GM103472 from the NIH to Dr. Vince Calhoun. *MCIC*: Department of Energy under Award Number DE-FG02-08ER64581. *NUSDAST*: NIMH Grant 1R01 MH084803. *NUNDA*: MH056584.	28,29,30,31,32,33
SCORE	Authors	This work was supported by the Swiss National Science Foundation (grant No. 119382).	34,35
STROKEMRI/ MOT	Authors	Supported by the Research Council of Norway (249795, 248238), the South-Eastern Norway Regional Health Authority (2014097, 2015044, 2015073, 2016083), and the Norwegian ExtraFoundation for Health and Rehabilitation (2015/FO5146).	36

TOP	Authors	The work was funded by the Research Council of Norway (213837, 223273, 204966/F20, 213694, 229129, 249795/F20, 248778), the South-Eastern Norway Regional Health Authority (2013-123, 2014-097, 2015-073, #2017-112) and Stiftelsen Kristian Gerhard Jebsen.	[37,38,39,40]
UKBB	https://www.ukbiobank.ac.uk/	All subjects with a primary or secondary ICD-10 diagnosis with a mental or neurological disorder were excluded prior to analysis and the remaining subjects included as healthy controls.	[41]
UNIBA	Authors	This work was supported by a “Capitale Umano ad Alta Qualificazione” grant by Fondazione Con Il Sud awarded to Alessandro Bertolino and by a Hoffmann-La Roche Collaboration Grant awarded to Giulio Pergola. This project has received funding from the European Union Seventh Framework Programme for research, technological development and demonstration under grant agreement no. 602450 (IMAGEMEND). This paper reflects only the author's views and the European Union is not liable for any use that may be made of the information contained therein.	[42]

Supplementary Table 2. Summary of magnetic resonance imaging characteristics of included samples.

**Sample**	**Number of scanners/ protocols included**	**Parameters**	**References**
ABIDE1	20	http://fcon_1000.projects.nitrc.org/indi/abide/scan_params/	[1]
ABIDE2	16		[2]
ABM	2	Philips 3T Ingenia: TR=3000ms, TE=3.61ms, FA=8° (2x same scanner and protocol, except for sagittal phase-encoding vs. axial phase encoding)	[3]
ADDNEUROMED	6	GE 1.5T: TR=8.59, TE=3.8, FA=8°<br>GE 1.5T: TR=10.4, TE=4.09, FA=9°<br>GE 1.5T: TR=10.2, TE=4.1, FA=8°<br>GE 1.5T: TR=10.2, TE=4.1, FA=8°<br>Siemens 1.5T: TR=2400, TE=3.5, FA=8°<br>Picker 1.5T: TR=13, TE=3, FA=20°	[4,5]
ADHD200	6	Philips 1.5 T Gyroscan: TR=8ms, TE=3.76ms, FA=8°;<br>Siemens 3T Allegra: TR=2530ms, TE=3.25ms, FA=8°;<br>Siemens 3T Trio: TR=2300ms, TE=3.58ms, 10°;<br>Siemens 3T Trio: TR=1700ms, TE=3.92ms, FA=12°<br>Siemens 3T Trio: TR=2100ms, TE=3.43ms, FA=8°<br>Siemens 3T Trio: TR=2400ms, TE=3.08ms, FA=8°	[6,7]
ADHDWUE	1	Siemens 1.5T Avanto: TR=2250ms, TE=3.93ms, FA=8°	[8,9]
ADNI1	54	http://adni.loni.usc.edu/methods/mri-tool/mri-analysis/	[10,11]
ADNI2	53		
CIMH	1	Siemens 3T Trio: TR=1570ms, TE=2.75ms, FA=15°	[12,13]
DS000030 (CNP)	2	Siemens 3T Trio: TR=1900ms, TE=2.26ms, FA=12°	[14,15]
DS000171	1	Siemens 3T Skyra: TR=2300ms, TE=2.01ms, FA=9°	[16]
HUBIN	1	GE 1.5 T signa Echo-speed: TR=24ms, TE=6.0ms, FA=35°	[17]
KASP	1	GE 3T Discovery MR750: TR=7.91ms, TE=3.06ms, FA=12°	[18,19]
MALTOSLO	1	Philips 3T Achieva: TR=8.4ms, TE=2.3ms, FA=7°	[20,21]
NIMAGE	2	Siemens 1.5T Sonata: TR= 2730ms, TE=2.95ms, FA=7°<br>Siemens 1.5T Avanto: TR= 2730ms, TE=2.95ms, FA=7°	[22]
NORCOG	3	GE 3T Signa HDxT: TR=7.8ms, TE=2.956ms, FA=12° (one subset with HNS coil, one subset with 8HRBRAIN coil)<br>GE 3T Discovery GE750: TR=8.16ms, TE=3.18ms, FA=12°	[23]
OASIS	1	Siemens 1.5T Vision: TR=9.7ms, TE=4ms, FA=10°	[24,25]
PPMI	3	http://www.ppmi-info.org/wp-content/uploads/2017/06/PPMI-MRI-Operations-Manual-V7.pdf	[26]
SCHIZCONNECT1 (BrainGluSchi, COBRE, MCIC)	5	Siemens 3T Trio: 2530ms, TE=TE = 1.64, 3.5, 5.36, 7.22, 9.08ms, FA=7°<br>Siemens 1.5T Sonata: TR=12ms, TE=4.76, FA=20°<br>Siemens 3T SMS Trio: TR=2530ms, TE=3.81ms, FA=7°<br>Siemens 1.5T Avanto: TR=12ms, TE=4.76ms, FA=20°	[28,29,30,31,32,33]
SCHIZCONNECT2 (NUNDA,	2	Siemens 3T Trio: TR=2400ms, TE=3.16ms, FA=8°<br>Siemens 1.5T Vision: TR=9.7ms, TE=4ms, FA=10°	

NUSDAST)			
SCORE	1	Siemens 1.5T Vision: TR=9.7ms, TE=4ms, FA=12°	[34,35]
STROKEMRI/ MOT	2	GE 3T Signa HDxT: TR=7.8ms, TE=2.956ms, FA=12°<br>GE 3T Discovery GE750: TR=8.16ms, TE=3.18ms, FA=12°	[36]
TOP/ RSI-MS	4	Siemens 1.5T Sonata: TR=2730ms, TE=3.93ms, FA=7°<br>GE 3T Signa HDxT: TR=7.8ms, TE=2.956ms, FA=12° (one subset with HNS coil, one subset with 8HRBRAIN coil)<br>GE 3T Discovery GE750: TR=8.16ms, TE=3.18ms, FA=12°	[27,37,38,39,40]
UKBB	3	Siemens 3T Skyra: TR=2000ms, TE=2.01ms, FA=8° (3 identical scanning sites)	[41]
UNIBA	1	GE 3T Signa: TR=25ms, TE=3ms, FA=6°	[42]

Supplementary Table 3. Size and demographic information of final study samples after quality control procedures.

Sample	Number of subjects	Age: mean ± sd in years (group)	Sex: f/m
GWAS discovery sample*			
UK Biobank participants	27,034	64.0±7.5	13,931/13,103
GWAS replication sample*			
UK Biobank participants	7,432	66.2±7.4	3,893/3,539
Clinical sample**			
ADHD	681 patients; 992 HC	17.5±9.9 (ADHD); 17.4±9.7 (HC)	702/971
ASD	125 patients; 140 HC	18.7±9.7 (ASD); 17.1±9.8 (HC)	34/231
BD	464 patients; 1,531 HC	33.7±10.7 (BD); 39.1±15.9 (HC)	1,031/946
MDD	211 patients; 93 HC	38.8±13.5 (MDD); 39.5±13.9 (HC)	201/103
PSYMIX	308 patients; 1,430 HC	28.9±9.3 (PSYMIX); 39.3±16.2 (HC)	852/886
SCZ	1,044 patients; 2,079 HC	33.6±11.0 (SCZ); 37.7±15.2 (HC)	1,323/1,800
SCZRISK	91 patients; 402 HC	23.7±5.0 (SCZRISK); 31.2±11.7 (HC)	223/270
DEM	756 patients; 1,921 HC	75.4±7.7 (DEM); 51.4±22.0 (HC)	1,434/1,243
MCI	987 patients; 1,655 HC	72.2±8.4 (MCI); 53.3±21.3 (HC)	1,264/1,378
MS	257 patients; 1,053 HC	40.9±10.0 (MS); 41.4±17.6 (HC)	745/565
PD	138 patients; 67 HC	61.2±9.1 (PD); 60.2±11.3 (HC)	73/132

GWAS; genome-wide association study. ADHD; attention-deficit/hyperactivity disorder. ASD; autism spectrum disorders. BD; bipolar disorder. HC; healthy controls. MDD; major depression. PSYMIX; non-SCZ psychosis spectrum diagnoses. SCZ; schizophrenia. SCZRISK; prodromal SCZ or at risk mental state. DEM; dementia. MCI; mild cognitive impairment. MS; multiple sclerosis. PD; Parkinson's disease. Please note that in some occasions where multiple disorders were studied on the same scanner, some of the same controls were used for different disorders. *All genetic analyses of the present study were run for the GWAS discovery and the GWAS replication sample.**Only a small minority in the clinical sample had genotype data and no genetic analyses were run for these individuals.

Supplementary Table 4. SNP-based heritability estimates for brainstem volumes
We estimated SNP-based heritability for the brainstem volumes, with and without accounting for whole brainstem volume using genome-wide complex trait analysis (GCTA) v1.92. For completeness, we also report heritabilities estimated with LD score regression v1.0.0.

	**Estimates from GCTA**		**Estimates from LD-score regression**				
	**Narrow-sense SNP-based heritability (SE)**	***P*-value**	**Narrow-sense SNP-based heritability (SE)**	**Lambda GC**	**Mean Chi^2**	**Intercept**	**Ratio**
**Whole brainstem**	**0.4781 (0.0212)**	<1e-16	**0.3196 (0.0312)**	11523	12003	1.015 (0.0092)	0.075 (0.0458)
**Midbrain incl. WBS**	**0.4673 (0.0215)**	<1e-16	**0.2881 (0.0289)**	11459	11917	1.0256 (0.0092)	0.1334 (0.0479)
**Pons incl. WBS**	**0.4719 (0.0215)**	<1e-16	**0.3057 (0.0335)**	11333	11903	1.0138 (0.0093)	0.0726 (0.049)
**SCP incl. WBS**	**0.2706 (0.0207)**	<1e-16	**0.1512 (0.0215)**	10988	11128	1.0267 (0.0078)	0.2366 (0.0696)
**Medulla incl. WBS**	**0.3467 (0.0212)**	<1e-16	**0.2262 (0.0277)**	11082	11399	1.0097 (0.0086)	0.0695 (0.0613)
**Midbrain**	**0.5016 (0.0212)**	<1e-16	**0.3292 (0.0299)**	11843	12114	1.0209 (0.0088)	0.0988 (0.0418)
**Pons**	**0.5499 (0.0212)**	<1e-16	**0.3462 (0.0331)**	11555	12242	1.0241 (0.0092)	0.1077 (0.0409)
**SCP**	**0.2993 (0.0208)**	<1e-16	**0.1856 (0.0233)**	1105	11213	1.0149 (0.0074)	0.1228 (0.0614)
**Medulla**	**0.2357 (0.0205)**	<1e-16	**0.2038 (0.0252)**	10926	11097	0.9905 (0.0089)	<0

GCTA; genome-wide complex trait analysis

SCP; superior cerebellar peduncle.

WBS; Whole brainstem

Supplementary Table 5. GWAS results in the replication sample.

We conducted genome-wide associaton studies (GWAS) of the brainstem volumes in an additional 7,432 participants from the UK Biobank. We found that all of the lead single nucleotide polymorphisms (SNPs) of the discovery sample had the same directions of effect for most of the volumes (all but midbrain and medulla oblongata volumes). We also found that the majority of the lead SNPs had uncorrected $P < 0.05$ in the GWAS replication sample. Two of the lead SNPs had $P <$ 5e-8 in the GWAS replication sample. The discovery and replication GWAS for all volumes were significantly correlated.

	**Replication of lead SNPs significant in discovery sample**							**Genetic correlation between discovery and replication GWAS**			
**Volume**	**Number of discovery SNPs (lead SNPs)**	**Number of SNPs that have the same effect direction**	**Percent of SNPs that have the same effect direction**	**Number of SNPs that replicate at *P*< 0.05**	**Percent of SNPs that replicate at *P* <.05**	**Number of SNPs that replicate at *P*< 5e-8**	**Percent of SNPs that replicate at *P* < 5e-8**	***P*-value**	R_g	***Z*-score**	***S.E.***
**Whole brainstem**	16	16	100	11	68,75	0	0	3,2E-17	0,75	8,4	0,09
**Midbrain incl. WBS**	10	7	70	4	40	0	0	1,6E-09	1,13	6,0	0,19
**Pons incl. WBS**	23	23	100	20	86,96	1	5	2,4E-14	0,9	7,6	0,12
**SCP incl. WBS**	3	3	100	2	66,67	0	0	5,7E-05	1,14	4,0	0,28
**Medulla incl. WBS**	9	8	88,89	7	77,78	0	0	1,9E-08	0,74	5,6	0,13
**Midbrain**	9	9	100	6	66,67	0	0	1,5E-17	0,75	8,5	0,09
**Pons**	18	18	100	14	77,78	1	7,1	4,8E-18	0,79	8,7	0,09
**SCP**	1	1	100	0	0	0	0	3,3E-05	1,01	4,2	0,24
**Medulla**	3	3	100	2	66,67	0	0	9,5E-11	0,75	6,5	0,11

S.E.; standard error.

Supplementary Table 6. Seventeen genes identified by both the three FUMA strategies and the genome-wide gene-based association studies.

Positional, expression quantitative trait loci (eQTL), and chromatin interaction mapping of the 125 independent significant single nucleotide polymorphisms identified 280 unique genes. The genome-wide gene-based association studies identified an additional 25 genes, thus resulting in a total of 305 brainstem-linked genes. Seventeen of these genes were identified by all four approaches and are shown below.

**Symbol**
CDK5RAP3
DLST
DRAM1
GINM1
GNPTAB
KATNA1
KIF1B
LATS1
MAPT
NLN
NUP43
PARPBP
POLR2B
RBBP5
RPS6KL1
SGTB
TRAPPC13

Supplementary Table 7. Gene sets implicated by the significant genes.

We conducted a gene-set analysis for curated gene sets and GO terms obtained from MsigDB (based on independent SNPs significant at the *P* < 5e-8 threshold) using hypergeometric tests in Functional Mapping and Annotation of GWAS (FUMA). The tests are described in detail on https://fuma.ctglab.nl/tutorial and all parameters used for the present study are available at https://fuma.ctglab.nl/browse/; ID 97-105. This identified 7 Gene Ontology sets significantly associated with whole brainstem volume, and 2, 8, 1, and 15 gene sets associated with volumes of the midbrain, pons, SCP, and medulla oblongata, respectively, after Bonferroni correction.

Volume	Category	GeneSet	No. of genes	No. of overlap	*P*-value	Adjusted *P*-value	Genes
Whole Brainstem	**GO_bp**	GO_SUSCEPTIBILITY_TO_NATURAL_KILLER_CELL_MEDIATED_CYTOTOXICITY	9	5	1.7923434137142382e-10	1.317372409079965e-06	RAET1E:RAET1G:ULBP2:ULBP1:ULBP3
		GO_POSITIVE_REGULATION_OF_NATURAL_KILLER_CELL_MEDIATED_IMMUNITY	28	5	1.309485502095423e-07	0.0009624718440401359	RAET1E:RAET1G:ULBP2:ULBP1:ULBP3
		GO_T_CELL_MEDIATED_CYTOTOXICITY	39	5	7.386482145853035e-07	0.0054290643772019805	RAET1E:RAET1G:ULBP2:ULBP1:ULBP3
		GO_REGULATION_OF_NATURAL_KILLER_CELL_MEDIATED_IMMUNITY	41	5	9.548243575273086e-07	0.007017959027825718	RAET1E:RAET1G:ULBP2:ULBP1:ULBP3
		GO_POSITIVE_REGULATION_OF_CELL_KILLING	56	5	4.622302565728259e-06	0.0339739238581027	RAET1E:RAET1G:ULBP2:ULBP1:ULBP3
		GO_NATURAL_KILLER_CELL_MEDIATED_IMMUNITY	58	5	5.506776847659903e-06	0.04047480983030029	RAET1E:RAET1G:ULBP2:ULBP1:ULBP3
	**GO_mf**	GO_NATURAL_KILLER_CELL_LECTIN_LIKE_RECEPTOR_BINDING	6	5	8.623652156012842e-12	1.4185907796641125e-08	RAET1E:RAET1G:ULBP2:ULBP1:ULBP3
Midbrain	**GO_bp**	GO_AMYLOID_BETA_FORMATION	27	4	1.766151373816949e-06	0.012981212597554574	IGF1:PSEN1:TMED10:DYRK1A
		GO_AMYLOID_PRECURSOR_PROTEIN_CATABOLIC_PROCESS	35	4	5.1663618661688405e-06	0.037972759716340974	IGF1:PSEN1:TMED10:DYRK1A
Pons	**GO_bp**	GO_SKELETAL_SYSTEM_MORPHOGENESIS	237	12	6.217489724737688e-09	4.569854947682201e-05	HOXB1:HOXB2:HOXB3:HOXB4:HOXB5:HOXB6:HOXB7:HOXB8:PHOSPHO1:BMP6:COL21A1:RAB23
		GO_ANTERIOR_POSTERIOR_PATTERN_SPECIFICATION	210	11	1.9495509858472783e-08	0.00014329199745977494	CTNNBIP1:HOXB1:HOXB2:HOXB3:HOXB4:HOXB5:HOXB6:HOXB7:HOXB8:HOXB9:HEY2
		GO_EMBRYONIC_SKELETAL_SYSTEM_DEVELOPMENT	126	9	2.8225578687274965e-08	0.000207458003351471	HOXB1:HOXB2:HOXB3:HOXB4:HOXB5:HOXB6:HOXB7:HOXB8:HOXB9
		GO_EMBRYONIC_SKELETAL_SYSTEM_MORPHOGENESIS	94	8	4.373688135016136e-08	0.00032146607792368597	HOXB1:HOXB2:HOXB3:HOXB4:HOXB5:HOXB6:HOXB7:HOXB8
		GO_SKELETAL_SYSTEM_DEVELOPMENT	513	15	1.1494116758627386e-07	0.0008448175817591129	IGF1:HOXB1:HOXB2:HOXB3:HOXB4:HOXB5:HOXB6:HOXB7:HOXB8:HOXB9:PHOSPHO1:BMP6:BMP5:COL21A1:RAB23
		GO_REGIONALIZATION	340	11	2.41786706409124e-06	0.017771322921070615	CTNNBIP1:HOXB1:HOXB2:HOXB3:HOXB4:HOXB5:HOXB6:HOXB7:HOXB8:HOXB9:HEY2
		GO_PATTERN_SPECIFICATION_PROCESS	434	12	4.167326542107681e-06	0.030629850084491452	CTNNBIP1:HOXB1:HOXB2:HOXB3:HOXB4:HOXB5:HOXB6:HOXB7:HOXB8:HOXB9:BMP5:HEY2
		GO_RHOMBOMERE_DEVELOPMENT	7	3	5.367326662849256e-06	0.039449850971942035	HOXB1:HOXB2:HOXB3
SCP	**GO_bp**	GO_DE_NOVO_IMP_BIOSYNTHETIC_PROCESS	6	2	4.49766757990721e-06	0.03305785671231799	PPAT:PAICS

	**GO_bp**	GO_EMBRYONIC_SKELETAL_SYSTEM_DEVELOPMENT	126	10	1.4013810214210626e-11	1.030015050744481e-07	WNT9B:HOXB1:HOXB2:HOXB3:HOXB4:HOXB5:HOXB6:HOXB7:HOXB8:HOXB9
		GO_EMBRYONIC_SKELETAL_SYSTEM_MORPHOGENESIS	94	9	2.8625237560971518e-11	2.1039549607314066e-07	WNT9B:HOXB1:HOXB2:HOXB3:HOXB4:HOXB5:HOXB6:HOXB7:HOXB8
		GO_ANTERIOR_POSTERIOR_PATTERN_SPECIFICATION	210	11	1.1360640962686847e-10	8.350071107574833e-07	CTNNBIP1:WNT3:HOXB1:HOXB2:HOXB3:HOXB4:HOXB5:HOXB6:HOXB7:HOXB8:HOXB9
		GO_EMBRYONIC_ORGAN_MORPHOGENESIS	283	11	2.680153256512953e-09	1.9699126435370202e-05	FZD2:CCDC103:WNT9B:HOXB1:HOXB2:HOXB3:HOXB4:HOXB5:HOXB6:HOXB7:HOXB8
		GO_SKELETAL_SYSTEM_MORPHOGENESIS	237	10	6.9057617794487664e-09	5.0757349078948434e-05	WNT9B:HOXB1:HOXB2:HOXB3:HOXB4:HOXB5:HOXB6:HOXB7:HOXB8:BMP6
		GO_EMBRYONIC_ORGAN_DEVELOPMENT	420	12	1.4999745250620965e-08	0.00011024812759206409	FZD2:CCDC103:WNT9B:HOXB1:HOXB2:HOXB3:HOXB4:HOXB5:HOXB6:HOXB7:HOXB8:EGFR
		GO_REGIONALIZATION	340	11	1.797058083055973e-08	0.000132083769104614	CTNNBIP1:WNT3:HOXB1:HOXB2:HOXB3:HOXB4:HOXB5:HOXB6:HOXB7:HOXB8:HOXB9
		GO_PATTERN_SPECIFICATION_PROCESS	434	12	2.1549308582384096e-08	0.0001583874180805231	CTNNBIP1:CCDC103:WNT3:HOXB1:HOXB2:HOXB3:HOXB4:HOXB5:HOXB6:HOXB7:HOXB8:HOXB9
		GO_EMBRYO_DEVELOPMENT_ENDING_IN_BIRTH_OR_EGG_HATCHING	619	13	1.3060603427305432e-07	0.0009599543519069493	FZD2:NMT1:WNT9B:HOXB1:HOXB2:HOXB3:HOXB4:HOXB5:HOXB6:HOXB7:HOXB8:HOXB9:EGFR
		GO_ANIMAL_ORGAN_MORPHOGENESIS	1016	16	1.955765735843936e-07	0.001437487815845293	CTNNBIP1:UBE4B:MTOR:FZD2:CCDC103:WNT9B:HOXB1:HOXB2:HOXB3:HOXB4:HOXB5:HOXB6:HOXB7:HOXB8:BMP6:EGFR
		GO_EMBRYONIC_MORPHOGENESIS	566	12	3.8362789478427483e-07	0.00281966502666442	FZD2:CCDC103:WNT3:WNT9B:HOXB1:HOXB2:HOXB3:HOXB4:HOXB5:HOXB6:HOXB7:HOXB8
		GO_EMBRYO_DEVELOPMENT	971	15	6.494261463964652e-07	0.004773282176014019	FZD2:CCDC103:NMT1:WNT3:WNT9B:HOXB1:HOXB2:HOXB3:HOXB4:HOXB5:HOXB6:HOXB7:HOXB8:HOXB9:EGFR
		GO_SKELETAL_SYSTEM_DEVELOPMENT	513	11	1.1135927809045891e-06	0.00818490693964873	WNT9B:HOXB1:HOXB2:HOXB3:HOXB4:HOXB5:HOXB6:HOXB7:HOXB8:HOXB9:BMP6
		GO_RHOMBOMERE_DEVELOPMENT	7	3	1.2953378296375518e-06	0.009520733047836006	HOXB1:HOXB2:HOXB3
	**GO_mf**	GO_DOUBLE_STRANDED_DNA_BINDING	939	13	1.339672214929791e-05	0.022037607935595064	APITD1:MTOR:MAPT:HOXB1:HOXB2:HOXB3:HOXB4:HOXB5:HOXB6:HOXB7:HOXB9:NFKB1:EGFR

SCP; superior cerebellar peduncle.

GO_bp; Gene Ontology biological process.

GO_mf; Gene Ontology molecular function.

Supplementary Table 8. Pathway analyses for mapped genes (based on independent SNPs significant at the *P* < 5e-8 threshold).

We used the ConsensusPathDB to identify over-represented pathways for the mapped genes (based on independent SNPs significant at the *P* < 5e-8 threshold). ConsensusPathDB is a database system that integrates functional interactions, including binary and complex protein-protein, genetic, metabolic, signaling, gene regulatory and drug-target interactions, as well as biochemical pathways. The details of the statistical analyses employed by the platform can be found on http://cpdb.molgen.mpg.de/. There were 13 significant pathways for whole brainstem volume, and 1, 25, and 58 signficant pathways for pons, SCP, and medulla oblongata volume after FDR-correction. There was no significant pathway for genes associated with midbrain volume.

Volume	Pathway Name	*P*-value	Adjusted *P*-value	Pathway source
Whole	Natural killer cell mediated cytotoxicity - Homo sapiens (human)	0.000301	0.0494	KEGG
	Processing of Capped Intron-Containing Pre-mRNA	0.000742	0.0494	Reactome
	Oxidative Damage	0.00078	0.0494	Wikipathways
	Trafficking and processing of endosomal TLR	0.00153	0.0494	Reactome
	classical complement pathway	0.00178	0.0494	BioCarta
	RHO GTPases Activate Formins	0.00233	0.0494	Reactome
	Osteoclast Signaling	0.00234	0.0494	Wikipathways
	Regulation of Insulin-like Growth Factor (IGF) transport and uptake by Insulin-like Growth Factor Binding Proteins (IGFBPs)	0.00254	0.0494	Reactome
	Endochondral Ossification	0.00291	0.0494	Wikipathways
	Integrins in angiogenesis	0.00291	0.0494	PID
	Polycystic Kidney Disease Pathway	0.00296	0.0494	Wikipathways
	Lamivudine Metabolism Pathway	0.00296	0.0494	SMPDB
	Lamivudine Pathway, Pharmacokinetics/Pharmacodynamics	0.00296	0.0494	PharmGKB
Pons	Activation of anterior HOX genes in hindbrain development during early embryogenesis	1.98e-07	2.69e-05	Wikipathways
SCP	Purine ribonucleoside monophosphate biosynthesis	1.64e-05	0.000261	Reactome
	Nucleobase biosynthesis	2.6e-05	0.000261	Reactome
	Purine metabolism - Homo sapiens (human)	7.81e-05	0.000261	KEGG
	Purine Nucleoside Phosphorylase Deficiency	0.000164	0.000261	SMPDB
	Xanthine Dehydrogenase Deficiency (Xanthinuria)	0.000164	0.000261	SMPDB
	Adenylosuccinate Lyase Deficiency	0.000164	0.000261	SMPDB
	AICA-Ribosiduria	0.000164	0.000261	SMPDB
	Adenine phosphoribosyltransferase deficiency (APRT)	0.000164	0.000261	SMPDB
	Mitochondrial DNA depletion syndrome	0.000164	0.000261	SMPDB

	Myoadenylate deaminase deficiency	0.000164	0.000261	SMPDB
	Purine Metabolism	0.000164	0.000261	SMPDB
	Molybdenum Cofactor Deficiency	0.000164	0.000261	SMPDB
	Adenosine Deaminase Deficiency	0.000164	0.000261	SMPDB
	Gout or Kelley-Seegmiller Syndrome	0.000164	0.000261	SMPDB
	Lesch-Nyhan Syndrome (LNS)	0.000164	0.000261	SMPDB
	Xanthinuria type I	0.000164	0.000261	SMPDB
	Xanthinuria type II	0.000164	0.000261	SMPDB
	Metabolic reprogramming in colon cancer	0.000212	0.000318	Wikipathways
	Mercaptopurine Action Pathway	0.000266	0.000342	SMPDB
	Azathioprine Action Pathway	0.000266	0.000342	SMPDB
	Thioguanine Action Pathway	0.000266	0.000342	SMPDB
	purine nucleotides *de novo* biosynthesis	0.00042	0.000515	HumanCyc
	Metabolism of nucleotides	0.00132	0.00155	Reactome
	Purine nucleotides nucleosides metabolism	0.00137	0.00155	INOH
	Huntington disease - Homo sapiens (human)	0.00439	0.00474	KEGG
Medulla	Activation of anterior HOX genes in hindbrain development during early embryogenesis	3.37e-08	5.39e-06	Wikipathways
	ESC Pluripotency Pathways	2.35e-05	0.00188	Wikipathways
	Breast cancer - Homo sapiens (human)	7.31e-05	0.00292	KEGG
	Gastric cancer - Homo sapiens (human)	7.8e-05	0.00292	KEGG
	Cushing syndrome - Homo sapiens (human)	9.12e-05	0.00292	KEGG
	Hepatocellular carcinoma - Homo sapiens (human)	0.000137	0.00363	KEGG
	Wnt Signaling in Kidney Disease	0.000181	0.00363	Wikipathways
	Class B/2 (Secretin family receptors)	0.000181	0.00363	Reactome
	EGFR-dependent Endothelin signaling events	0.000312	0.00506	PID
	Proteoglycans in cancer - Homo sapiens (human)	0.000316	0.00506	KEGG
	Wnt Canonical	0.00043	0.00573	INOH
	Wnt Mammals	0.00043	0.00573	INOH
	Human papillomavirus infection - Homo sapiens (human)	0.000476	0.00586	KEGG
	Signaling pathways regulating pluripotency of stem cells - Homo sapiens (human)	0.000772	0.00822	KEGG

LPA receptor mediated events	0.000877	0.00822	PID
Basal cell carcinoma - Homo sapiens (human)	0.000877	0.00822	KEGG
Brain-Derived Neurotrophic Factor (BDNF) signaling pathway	0.000881	0.00822	Wikipathways
Wnt signaling pathway - Homo sapiens (human)	0.000975	0.00822	KEGG
mTOR signaling pathway - Homo sapiens (human)	0.00103	0.00822	KEGG
Regulation of Telomerase	0.00109	0.00822	PID
Hippo signaling pathway - Homo sapiens (human)	0.0011	0.00822	KEGG
Breast cancer pathway	0.00113	0.00822	Wikipathways
Pancreatic cancer - Homo sapiens (human)	0.00145	0.0101	KEGG
ncRNAs involved in Wnt signaling in hepatocellular carcinoma	0.00215	0.0144	Wikipathways
Pancreatic adenocarcinoma pathway	0.00238	0.0152	Wikipathways
WNT ligand biogenesis and trafficking	0.00252	0.0155	Reactome
Prostate cancer - Homo sapiens (human)	0.00303	0.0161	KEGG
LncRNA involvement in canonical Wnt signaling and colorectal cancer	0.00303	0.0161	Wikipathways
Wnt signaling network	0.00316	0.0161	PID
Choline metabolism in cancer - Homo sapiens (human)	0.00321	0.0161	KEGG
HIF-1 signaling pathway - Homo sapiens (human)	0.00331	0.0161	KEGG
Melanogenesis - Homo sapiens (human)	0.0034	0.0161	KEGG
Wnt Signaling Pathway and Pluripotency	0.0034	0.0161	Wikipathways
MicroRNAs in cardiomyocyte hypertrophy	0.0035	0.0161	Wikipathways
Extracellular vesicle-mediated signaling in recipient cells	0.00362	0.0161	Wikipathways
Factors and pathways affecting insulin-like growth factor (IGF1)-Akt signaling	0.00362	0.0161	Wikipathways
JAK-STAT	0.0039	0.0169	Wikipathways
Pathways in cancer - Homo sapiens (human)	0.00447	0.0188	KEGG
Wnt Signaling	0.00478	0.0196	Wikipathways
CRH	0.00519	0.0207	NetPath
IL2 signaling events mediated by PI3K	0.00547	0.0214	PID
Oncostatin_M	0.00576	0.022	NetPath
Gastrin	0.00669	0.0249	NetPath
Hair Follicle Development- Induction (Part 1 of 3)	0.00701	0.0254	Wikipathways

	LKB1 signaling events	0.00734	0.0254	PID
	GPCR signaling-G alpha i	0.00764	0.0254	INOH
	GPCR signaling-pertussis toxin	0.00764	0.0254	INOH
	miRNA regulation of prostate cancer signaling pathways	0.00801	0.0254	Wikipathways
	RAGE	0.00801	0.0254	NetPath
	Aryl Hydrocarbon Receptor	0.00836	0.0254	Wikipathways
	ATM Signaling Network in Development and Disease	0.00836	0.0254	Wikipathways
	BDNF	0.00836	0.0254	NetPath
	GPCR signaling-cholera toxin	0.00847	0.0254	INOH
	GPCR signaling-G alpha s Epac and ERK	0.00869	0.0254	INOH
	Thymic Stromal LymphoPoietin (TSLP) Signaling Pathway	0.00872	0.0254	Wikipathways
	GPCR signaling-G alpha q	0.00891	0.0255	INOH
	Signaling by WNT	0.00914	0.0256	Reactome
	Retrograde transport at the Trans-Golgi-Network	0.00945	0.0261	Reactome

SCP; superior cerebellar peduncle.

Supplementary Table 9. Gene sets implicated by the significant genes (based on independent SNPs significant at the *P* < 1e-8 threshold).

We conducted a MAGMA gene-set analysis for curated gene sets and GO terms obtained from MsigDB (based on independent SNPs significant at the *P* < 1e-8 threshold) using hypergeometric tests in Functional Mapping and Annotation of GWAS (FUMA). The tests are described in detail on https://fuma.ctglab.nl/tutorial and all parameters used for the present study are available at https://fuma.ctglab.nl/browse/; ID 97-105. This identified 10 Gene Ontology sets significantly associated with whole brainstem volume, and 1, 6, and 16 gene sets associated with volumes of the midbrain, pons, and medulla oblongata, respectively.

**Volume**	**Category**	**GeneSet**	**No. of genes**	**No. of overlap**	***P*-value**	**Adjusted *P*-value**	**Genes**
Whole Brainstem	GO_bp	GO_SUSCEPTIBILITY_TO_NATURAL_KILLER_CELL_MEDIATED_CYTOTOXICITY	9	5	8.01119679395381e-12	5.88822964355605e-08	RAET1E:RAET1G:ULBP2:ULBP1:ULBP3
		GO_POSITIVE_REGULATION_OF_NATURAL_KILLER_CELL_MEDIATED_IMMUNITY	28	5	6.040152694634038e-09	4.4395122305560175e-05	RAET1E:RAET1G:ULBP2:ULBP1:ULBP3
		GO_T_CELL_MEDIATED_CYTOTOXICITY	39	5	3.4696592332186865e-08	0.00025501995364157347	RAET1E:RAET1G:ULBP2:ULBP1:ULBP3
		GO_REGULATION_OF_NATURAL_KILLER_CELL_MEDIATED_IMMUNITY	41	5	4.4999568411185646e-08	0.0003307468278222145	RAET1E:RAET1G:ULBP2:ULBP1:ULBP3
		GO_POSITIVE_REGULATION_OF_CELL_KILLING	56	5	2.2330613688891814e-07	0.0016413001061335484	RAET1E:RAET1G:ULBP2:ULBP1:ULBP3
		GO_NATURAL_KILLER_CELL_MEDIATED_IMMUNITY	58	5	2.6691483574181257e-07	0.001961824042702322	RAET1E:RAET1G:ULBP2:ULBP1:ULBP3
		GO_NATURAL_KILLER_CELL_ACTIVATION	80	5	1.346404683520293e-06	0.009896074423874154	RAET1E:RAET1G:ULBP2:ULBP1:ULBP3
		GO_REGULATION_OF_CELL_KILLING	80	5	1.346404683520293e-06	0.009896074423874154	RAET1E:RAET1G:ULBP2:ULBP1:ULBP3
		GO_T_CELL_MEDIATED_IMMUNITY	92	5	2.6958561941020196e-06	0.019814543026649846	RAET1E:RAET1G:ULBP2:ULBP1:ULBP3
	GO_mf	GO_NATURAL_KILLER_CELL_LECTIN_LIKE_RECEPTOR_BINDING	6	5	3.835361344074081e-13	6.309169411001863e-10	RAET1E:RAET1G:ULBP2:ULBP1:ULBP3
Midbrain	GO_cc	GO_GAMMA_SECRETASE_COMPLEX	6	2	3.7919874414266974e-05	0.03795779428868124	PSEN1:TMED10
Pons	GO_bp	GO_SKELETAL_SYSTEM_MORPHOGENESIS	237	12	5.5603361368448275e-09	4.0868470605809485e-05	HOXB1:HOXB2:HOXB3:HOXB4:HOXB5:HOXB6:HOXB7:HOXB8:PHOSPHO1:BMP6:COL21A1:RAB23
		GO_ANTERIOR_POSTERIOR_PATTERN_SPECIFICATION	210	11	1.7600944654491233e-08	0.00012936694321051057	CTNNBIP1:HOXB1:HOXB2:HOXB3:HOXB4:HOXB5:HOXB6:HOXB7:HOXB8:HOXB9:HEY2
		GO_EMBRYONIC_SKELETAL_SYSTEM_DEVELOPMENT	126	9	2.5925514634564835e-08	0.00019055253256405153	HOXB1:HOXB2:HOXB3:HOXB4:HOXB5:HOXB6:HOXB7:HOXB8:HOXB9
		GO_EMBRYONIC_SKELETAL_SYSTEM_MORPHOGENESIS	94	8	4.053437116652298e-08	0.0002979276280739439	HOXB1:HOXB2:HOXB3:HOXB4:HOXB5:HOXB6:HOXB7:HOXB8
		GO_SKELETAL_SYSTEM_DEVELOPMENT	513	14	6.399637147608658e-07	0.004703733303492363	IGF1:HOXB1:HOXB2:HOXB3:HOXB4:HOXB5:HOXB6:HOXB7:HOXB8:HOXB9:PHOSPHO1:BMP6:COL21A1:RAB23
		GO_REGIONALIZATION	340	11	2.1965131337139024e-06	0.016144371532797182	CTNNBIP1:HOXB1:HOXB2:HOXB3:HOXB4:HOXB5:HOXB6:HOXB7:HOXB8:HOXB9:HEY2
Medulla	GO_bp	GO_EMBRYONIC_SKELETAL_SYSTEM_DEVELOPMENT	126	9	7.927508608132452e-13	5.826718826977352e-09	HOXB1:HOXB2:HOXB3:HOXB4:HOXB5:HOXB6:HOXB7:HOXB8:HOXB9
		GO_ANTERIOR_POSTERIOR_PATTERN_SPECIFICATION	210	10	2.1234793826571546e-12	1.5607573462530086e-08	CTNNBIP1:HOXB1:HOXB2:HOXB3:HOXB4:HOXB5:HOXB6:HOXB7:HOXB8:HOXB9
		GO_EMBRYONIC_SKELETAL_SYSTEM_MORPHOGENESIS	94	8	4.079767075881422e-12	2.9986288007728455e-08	HOXB1:HOXB2:HOXB3:HOXB4:HOXB5:HOXB6:HOXB7:HOXB8

		GO_SKELETAL_SYSTEM_MORPHOGENESIS	237	9	2.3532428612677264e-10	1.729633503031779e-06	HOXB1:HOXB2:HOXB3:HOXB4:HOXB5:HOXB6:HOXB7:HOXB8:BMP6
		GO_REGIONALIZATION	340	10	2.460608309699795e-10	1.8085471076293493e-06	CTNNBIP1:HOXB1:HOXB2:HOXB3:HOXB4:HOXB5:HOXB6:HOXB7:HOXB8:HOXB9
		GO_PATTERN_SPECIFICATION_PROCESS	434	10	2.609478319454871e-09	1.9179665647993303e-05	CTNNBIP1:HOXB1:HOXB2:HOXB3:HOXB4:HOXB5:HOXB6:HOXB7:HOXB8:HOXB9
		GO_ANIMAL_ORGAN_MORPHOGENESIS	1016	13	8.04344834485031e-09	5.911934533464978e-05	CTNNBIP1:UBE4B:MTOR:HOXB1:HOXB2:HOXB3:HOXB4:HOXB5:HOXB6:HOXB7:HOXB8:BMP6:EGFR
		GO_SKELETAL_SYSTEM_DEVELOPMENT	513	10	1.288286899414934e-08	9.468908710699764e-05	HOXB1:HOXB2:HOXB3:HOXB4:HOXB5:HOXB6:HOXB7:HOXB8:HOXB9:BMP6
		GO_EMBRYONIC_ORGAN_MORPHOGENESIS	283	8	2.6898621940046634e-08	0.00019770487125934276	HOXB1:HOXB2:HOXB3:HOXB4:HOXB5:HOXB6:HOXB7:HOXB8
		GO_EMBRYONIC_ORGAN_DEVELOPMENT	420	9	3.490535126869775e-08	0.00025655433182492847	HOXB1:HOXB2:HOXB3:HOXB4:HOXB5:HOXB6:HOXB7:HOXB8:EGFR
		GO_EMBRYO_DEVELOPMENT_ENDING_IN_BIRTH_OR_EGG_HATCHING	619	10	7.562945259501335e-08	0.0005558764765733481	HOXB1:HOXB2:HOXB3:HOXB4:HOXB5:HOXB6:HOXB7:HOXB8:HOXB9:EGFR
		GO_RHOMBOMERE_DEVELOPMENT	7	3	1.7832224957880893e-07	0.0013106685344042455	HOXB1:HOXB2:HOXB3
	GO_mf	GO_DOUBLE_STRANDED_DNA_BINDING	939	11	3.774439093029059e-07	0.0006208952308032801	APITD1:MTOR:HOXB1:HOXB2:HOXB3:HOXB4:HOXB5:HOXB6:HOXB7:HOXB9:EGFR
		GO_SEQUENCE_SPECIFIC_DOUBLE_STRANDED_DNA_BINDING	850	9	1.2383694296310627e-05	0.020371177117430982	MTOR:HOXB1:HOXB2:HOXB3:HOXB4:HOXB5:HOXB6:HOXB7:HOXB9
		GO_SEQUENCE_SPECIFIC_DNA_BINDING	1102	10	1.4137102669923379e-05	0.023255533892023957	MTOR:HOXB1:HOXB2:HOXB3:HOXB4:HOXB5:HOXB6:HOXB7:HOXB8:HOXB9
		GO_REGULATORY_REGION_NUCLEIC_ACID_BINDING	922	9	2.366885472446812e-05	0.038935266021750056	MTOR:HOXB1:HOXB2:HOXB3:HOXB4:HOXB5:HOXB6:HOXB7:HOXB9

GO_bp; Gene Ontology biological process.

GO_mf; Gene Ontology molecular function.

GO_cc; Gene Ontology cellular component.

Supplementary Table 10. Pathway analyses for mapped genes (based on independent SNPs significant at the *P* < 1e-8 threshold).

We used the ConsensusPathDB to identify over-represented pathways for the mapped genes (based on independent SNPs significant at the *P* < 1e-8 threshold). ConsensusPathDB is a database system that integrates functional interactions, including binary and complex protein-protein, genetic, metabolic, signaling, gene regulatory and drug-target interactions, as well as biochemical pathways. The details of the statistical analyses employed by the platform can be found on http://cpdb.molgen.mpg.de/. There were 53, 19, 1, and 4 significant pathways for volumes of the whole brainstem, midbrain, pons, and medulla oblongata, respectively, after FDR-correction.

**Volume**	**Pathway Name**	***P*-value**	**Adjusted *P*-value**	**Pathway source**
Whole	Natural killer cell mediated cytotoxicity - Homo sapiens (human)	1.28e-05	0.00173	KEGG
	Processing of Capped Intron-Containing Pre-mRNA	0.000237	0.0154	Reactome
	Polycystic Kidney Disease Pathway	0.000823	0.0154	Wikipathways
	Lamivudine Metabolism Pathway	0.000823	0.0154	SMPDB
	Lamivudine Pathway, Pharmacokinetics/Pharmacodynamics	0.000823	0.0154	PharmGKB
	Amplification of signal from unattached kinetochores via a MAD2 inhibitory signal	0.00151	0.0154	Reactome
	Amplification of signal from the kinetochores	0.00151	0.0154	Reactome
	Vitamin D3 (cholecalciferol) metabolism	0.00186	0.0154	EHMN
	Pentose phosphate pathway	0.00215	0.0154	EHMN
	Resolution of Sister Chromatid Cohesion	0.00218	0.0154	Reactome
	NEP/NS2 Interacts with the Cellular Export Machinery	0.0023	0.0154	Reactome
	Transport of Ribonucleoproteins into the Host Nucleus	0.0023	0.0154	Reactome
	Regulation of Glucokinase by Glucokinase Regulatory Protein	0.0023	0.0154	Reactome
	Mitotic Spindle Checkpoint	0.00241	0.0154	Reactome
	Vitamin B5 - CoA biosynthesis from pantothenate	0.00245	0.0154	EHMN
	Prostaglandin formation from dihomo gama-linoleic acid	0.00245	0.0154	EHMN
	Export of Viral Ribonucleoproteins from Nucleus	0.00245	0.0154	Reactome
	Vpr-mediated nuclear import of PICs	0.00261	0.0154	Reactome
	Rev-mediated nuclear export of HIV RNA	0.00278	0.0154	Reactome
	Transport of the SLBP independent Mature mRNA	0.00278	0.0154	Reactome
	Nuclear import of Rev protein	0.00295	0.0154	Reactome
	Transport of the SLBP Dependant Mature mRNA	0.00295	0.0154	Reactome

	Nuclear Pore Complex (NPC) Disassembly	0.00295	0.0154	Reactome
	RHO GTPases Activate Formins	0.00307	0.0154	Reactome
	Interactions of Vpr with host cellular proteins	0.00312	0.0154	Reactome
	Selenoamino acid metabolism	0.0033	0.0154	EHMN
	Squalene and cholesterol biosynthesis	0.00348	0.0154	EHMN
	SUMOylation of DNA replication proteins	0.00348	0.0154	Reactome
	Interactions of Rev with host cellular proteins	0.00348	0.0154	Reactome
	Separation of Sister Chromatids	0.00351	0.0154	Reactome
	Transport of Mature mRNA Derived from an Intronless Transcript	0.00406	0.0154	Reactome
	Glycerophospholipid metabolism	0.00407	0.0154	EHMN
	Transport of Mature mRNAs Derived from Intronless Transcripts	0.00426	0.0154	Reactome
	Mitotic Anaphase	0.00441	0.0154	Reactome
	Putative anti-Inflammatory metabolites formation from EPA	0.00447	0.0154	EHMN
	Viral Messenger RNA Synthesis	0.00447	0.0154	Reactome
	Mitotic Metaphase and Anaphase	0.0045	0.0154	Reactome
	Vitamin E metabolism	0.00468	0.0154	EHMN
	De novo fatty acid biosynthesis	0.00468	0.0154	EHMN
	Deposition of new CENPA-containing nucleosomes at the centromere	0.00468	0.0154	Reactome
	Nucleosome assembly	0.00468	0.0154	Reactome
	Influenza Viral RNA Transcription and Replication	0.0049	0.0157	Reactome
	SUMOylation of RNA binding proteins	0.00534	0.0168	Reactome
	Vitamin B3 (nicotinate and nicotinamide) metabolism	0.00557	0.0171	EHMN
	Nuclear Envelope Breakdown	0.00653	0.0191	Reactome
	Aminosugars metabolism	0.00653	0.0191	EHMN
	snRNP Assembly	0.00678	0.0191	Reactome
	Metabolism of non-coding RNA	0.00678	0.0191	Reactome
	Influenza Life Cycle	0.00704	0.0194	Reactome
	tRNA processing in the nucleus	0.0081	0.0219	Reactome
	mRNA Splicing - Major Pathway	0.00857	0.0227	Reactome
	Lysine metabolism	0.00895	0.0232	EHMN

	mRNA Splicing	0.00967	0.0241	Reactome
Midbrain	Extracellular vesicles in the crosstalk of cardiac cells	0.000619	0.0188	Wikipathways
	NOTCH3 Activation and Transmission of Signal to the Nucleus	0.000802	0.0188	Reactome
	Syndecan-3-mediated signaling events	0.000868	0.0188	PID
	Factors and pathways affecting insulin-like growth factor (IGF1)-Akt signaling	0.00116	0.0188	Wikipathways
	Nuclear Pore Complex (NPC) Disassembly	0.00149	0.0193	Reactome
	Stabilization and expansion of the E-cadherin adherens junction	0.00226	0.0201	PID
	Signaling by ERBB4	0.00248	0.0201	Reactome
	Signaling by NOTCH3	0.00248	0.0201	Reactome
	Nuclear Envelope Breakdown	0.00332	0.022	Reactome
	Focal Adhesion	0.00434	0.022	Wikipathways
	Focal adhesion - Homo sapiens (human)	0.0044	0.022	KEGG
	SHP2 signaling	0.00442	0.022	PID
	Lysine metabolism	0.00456	0.022	EHMN
	Rap1 signaling pathway - Homo sapiens (human)	0.00484	0.022	KEGG
	Signaling by Receptor Tyrosine Kinases	0.00508	0.022	Reactome
	Glioma - Homo sapiens (human)	0.00634	0.0243	KEGG
	Melanoma - Homo sapiens (human)	0.00651	0.0243	KEGG
	Ras signaling pathway - Homo sapiens (human)	0.00673	0.0243	KEGG
	Signaling by NOTCH3	0.00686	0.026	Reactome
Pons	Activation of anterior HOX genes in hindbrain development during early embryogenesis	2.26e-07	2.98e-05	Wikipathways
Medulla	Activation of anterior HOX genes in hindbrain development during early embryogenesis	4.39e-09	2.59e-07	Wikipathways
	EGFR-dependent Endothelin signaling events	0.000117	0.00345	PID
	Choline metabolism in cancer - Homo sapiens (human)	0.000773	0.0152	KEGG
	Extracellular vesicle-mediated signaling in recipient cells	0.00138	0.0203	Wikipathways

Supplementary Table 11. Gene Ontology gene sets analyses for genes nearest to the loci shared between brainstem volumes and disorders.

We ran Gene Ontology gene sets analyses for genes nearest to the shared loci across the brainstem regions for each disorder. There were 33 significant gene sets for schizophrenia, mainly involving central nervous system, neuronal, and cellular developmental processes. There were no significant gene sets for the other disorders.

Category	Gene Set	No. of genes	No. of overlap	*P*-value	Adjusted *P*-value	Genes
**GO_bp**	GO_MOVEMENT_OF_CELL_OR_SUBCELLULAR_COMPONENT	1272	9	1.856846340334883e-07	0.0008236970365725542	RERE:CAPZA1:CNTN2:SDCCAG8:KIF20B:DGKZ:MAPT:CNTN4:MITF
	GO_NEUROGENESIS	1401	8	5.140422139725262e-06	0.0086128854209787	RERE:CNTN2:SDCCAG8:KIF20B:ATF1:MAPT:GIGYF2:CNTN4
	GO_CELL_DEVELOPMENT	1425	8	5.824764712113639e-06	0.0086128854209787	RERE:CNTN2:KIF20B:MAPT:CNTN4:MEGF10:BAK1:TRPS1
	GO_REGULATION_OF_CELL_DIFFERENTIATION	1488	8	8.000593646661305e-06	0.008872658354147387	CNTN2:KIF20B:ATF1:MAPT:CNTN4:MITF:MEGF10:TRPS1
	GO_NEURON_PROJECTION_MORPHOGENESIS	402	5	1.0312643565159117e-05	0.00889471017676517	RERE:CNTN2:KIF20B:MAPT:CNTN4
	GO_LOCOMOTION	1111	7	1.2030717101125119e-05	0.00889471017676517	RERE:CNTN2:SDCCAG8:DGKZ:MAPT:CNTN4:MITF
	GO_REGULATION_OF_MULTICELLULAR_ORGANISMAL_DEVELOPMENT	1667	8	1.8287076126414264e-05	0.011588781385253382	CNTN2:KIF20B:ATF1:MAPT:CNTN4:MITF:MEGF10:TRPS1
	GO_CELL_MOTILITY	832	6	2.6560860506645116e-05	0.01401891280259344	RERE:CNTN2:SDCCAG8:DGKZ:MAPT:MITF
	GO_ADULT_LOCOMOTORY_BEHAVIOR	80	3	2.8442338869103012e-05	0.01401891280259344	CNTN2:MAPT:GIGYF2
	GO_CENTRAL_NERVOUS_SYSTEM_DEVELOPMENT	869	6	3.3885906573347213e-05	0.014110361909678737	RERE:CNTN2:MAPT:GIGYF2:CNTN4:BAK1
	GO_NEURON_DIFFERENTIATION	874	6	3.498962601588505e-05	0.014110361909678737	RERE:CNTN2:KIF20B:MAPT:GIGYF2:CNTN4
	GO_NEURON_PROJECTION_DEVELOPMENT	545	5	4.425842750793661e-05	0.01604487167899327	RERE:CNTN2:KIF20B:MAPT:CNTN4
	GO_TUBE_DEVELOPMENT	552	5	4.702058877973682e-05	0.01604487167899327	SDCCAG8:KIF20B:RASIP1:BAK1:TRPS1
	GO_NEURON_MIGRATION	110	3	7.362153516089638e-05	0.02332750928383831	CNTN2:SDCCAG8:MAPT
	GO_TISSUE_DEVELOPMENT	1508	7	8.468544826040797e-05	0.023417607242330572	KIF20B:RASIP1:MITF:MEGF10:BAK1:TRPS1:ZDHHC21
	GO_TUBE_MORPHOGENESIS	323	4	8.927580432599122e-05	0.023417607242330572	SDCCAG8:KIF20B:RASIP1:BAK1
	GO_CELL_PART_MORPHOGENESIS	633	5	8.974285913426955e-05	0.023417607242330572	RERE:CNTN2:KIF20B:MAPT:CNTN4
	GO_AXONAL_FASCICULATION	20	2	9.919547611212955e-05	0.024446174001855926	CNTN2:CNTN4
	GO_NLS_BEARING_PROTEIN_IMPORT_INTO_NUCLEUS	22	2	0.00012049108574255533	0.02813149770284081	RERE:TRPS1
	GO_NEURON_DEVELOPMENT	687	5	0.00013168946531012883	0.028542994142964177	RERE:CNTN2:KIF20B:MAPT:CNTN4
	GO_ADULT_BEHAVIOR	135	3	0.00013512237984721545	0.028542994142964177	CNTN2:MAPT:GIGYF2
	GO_CELL_MORPHOGENESIS_INVOLVED_IN_NEURON_DIFFERENTIATION	368	4	0.00014740399663681295	0.02884614859264598	RERE:CNTN2:MAPT:CNTN4
	GO_HEAD_DEVELOPMENT	706	5	0.00014956298864536913	0.02884614859264598	RERE:CNTN2:MAPT:CNTN4:BAK1
	GO_CELL_RECOGNITION	147	3	0.00017374801495156567	0.032015496000209184	CNTN2:CNTN4:MEGF10

	GO_REGULATION_OF_ORGANELLE_ORGANIZATION	1177	6	0.0001804299819669138	0.032015496000209184	CAPZA1:KCNN3:KIF20B:MAPT:BAK1:TRPS1
	GO_REGULATION_OF_NERVOUS_SYSTEM_DEVELOPMENT	749	5	0.00019687830432854868	0.03359046761544007	CNTN2:KIF20B:ATF1:MAPT:CNTN4
	GO_MITOTIC_CELL_CYCLE	766	5	0.00021845291825760572	0.03589100538484218	SDCCAG8:KIF20B:DGKZ:GIGYF2:NEK4
	GO_WALKING_BEHAVIOR	31	2	0.00024155568806227224	0.038269322580151414	CNTN2:MAPT
	GO_MORPHOGENESIS_OF_A_BRANCHING_STRUCTURE	167	3	0.00025289012687281295	0.03868346906233787	RERE:RASIP1:BAK1
	GO_NEURON_RECOGNITION	33	2	0.0002740331272130638	0.040520365077238364	CNTN2:CNTN4
	GO_ION_TRANSMEMBRANE_TRANSPORT	820	5	0.0002991500146597133	0.042807402097757685	KCNN3:SFXN2:SLC39A8:BAK1:SLC16A10
	GO_LOCOMOTORY_BEHAVIOR	181	3	0.000320219104392674	0.0434675097939459	CNTN2:MAPT:GIGYF2
	GO_REGULATION_OF_CELL_DEVELOPMENT	834	5	0.00032336064544639644	0.0434675097939459	CNTN2:KIF20B:ATF1:MAPT:CNTN4

GO_bp; Gene Ontology biological process.

Supplementary References

1. Di Martino, A., et al. The autism brain imaging data exchange: towards a large-scale evaluation of the intrinsic brain architecture in autism. *Mol Psychiatry* **19**, 659-667 (2014).

2. Di Martino, A., et al. Enhancing studies of the connectome in autism using the autism brain imaging data exchange II. *Sci. Data* **4**, 170010 (2017).

3. Maglanoc, L. A., et al. Data-Driven Clustering Reveals a Link Between Symptoms and Functional Brain Connectivity in Depression. *Biol. Psychiatry Cogn. Neurosci. Neuroimaging* **4**, 16-26 (2019).

4. Liu, Y., et al. Combination analysis of neuropsychological tests and structural MRI measures in differentiating AD, MCI and control groups--the AddNeuroMed study. *Neurobiol. Aging* **32**, 1198-1206 (2011).

5. Lovestone, S., Francis, P. & Strandgaard, K. Biomarkers for disease modification trials--the innovative medicines initiative and AddNeuroMed. *J. Nutr. Health Aging* **11**, 359-361 (2007).

6. Brown, M. R., et al. ADHD-200 Global Competition: diagnosing ADHD using personal characteristic data can outperform resting state fMRI measurements. *Front. Syst. Neurosci* **6**, 69 (2012).

7. Consortium, H. D. The ADHD-200 Consortium: A Model to Advance the Translational Potential of Neuroimaging in Clinical Neuroscience. *Front. Syst. Neurosci* **6**, 62 (2012).

8. Guadalupe, T., et al. Human subcortical brain asymmetries in 15,847 people worldwide reveal effects of age and sex. *Brain Imaging Behav.* **11**, 1497-1514 (2017).

9. Conzelmann, A., et al. Abnormal affective responsiveness in attention-deficit/hyperactivity disorder: subtype differences. *Biol. Psychiatry* **65**, 578-585 (2009).

10. Weiner, M. W., et al. The Alzheimer's disease neuroimaging initiative: progress report and future plans. *Alzheimers Dement.* **6**, 202-211 e207 (2010).

11. Wyman, B. T., et al. Standardization of analysis sets for reporting results from ADNI MRI data. *Alzheimers Dement.* **9**, 332-337 (2013).

12. Eisenacher, S., et al. Investigation of metamemory functioning in the at-risk mental state for psychosis. *Psychol. Med.* **45**, 3329-3340 (2015).

13. Rausch, F., et al. Reduced activation in ventral striatum and ventral tegmental area during probabilistic decision-making in schizophrenia. *Schizophr. Res.* **156**, 143-149 (2014).

14. Gorgolewski, K. J., Durnez, J. & Poldrack, R. A. Preprocessed Consortium for Neuropsychiatric Phenomics dataset. *F1000Res.* **6**, 1262 (2017).

15. Poldrack, R. A., et al. A phenome-wide examination of neural and cognitive function. *Sci. Data* **3**, 160110 (2016).

16. Lepping, R. J., Ruth, A. A. & Cary, R. Development of a validated emotionally provocative musical stimulus set for researc. *Psychology of music* **44**, (2016).

17. Haukvik, U. K., et al. Cortical folding in Broca's area relates to obstetric complications in schizophrenia patients and healthy controls. *Psychol Med.* **42**, 1329-1337 (2012).

18. Collste, K., et al. Lower levels of the glial cell marker TSPO in drug-naive first-episode psychosis patients as measured using PET and [(11)C]PBR28. *Mol. Psychiatry* **22**, 850-856 (2017).

19. Orhan, F., et al. CSF GABA is reduced in first-episode psychosis and associates to symptom severity. *Mol. Psychiatry* **23**, 1244-1250 (2018).

20. Elvsashagen, T., et al. Bipolar II disorder is associated with thinning of prefrontal and temporal cortices involved in affect regulation. *Bipolar Disord.* **15**, 855-864 (2013).

21. Elvsashagen, T., et al. Evidence for reduced dentate gyrus and fimbria volume in bipolar II disorder. *Bipolar Disord.* **15**, 167-176 (2013).

22. von Rhein, D., et al. The NeuroIMAGE study: a prospective phenotypic, cognitive, genetic and MRI study in children with attention-deficit/hyperactivity disorder. Design and descriptives. *Eur. Child. Adolesc. Psychiatry* **24**, 265-281 (2015).

23. Doan, N. T., et al. Distinguishing early and late brain aging from the Alzheimer's disease spectrum: consistent morphological patterns across independent samples. *Neuroimage* **158**, 282-295 (2017).

24. Buckner, R. L., et al. A unified approach for morphometric and functional data analysis in young, old, and demented adults using automated atlas-based head size normalization: reliability and validation against manual measurement of total intracranial volume. *Neuroimage* **23**, 724-738 (2004).

25. Fotenos, A. F., Snyder, A. Z., Girton, L. E., Morris, J. C. & Buckner, R. L. Normative estimates of cross-sectional and longitudinal brain volume decline in aging and AD. *Neurology* **64**, 1032-1039 (2005).

26. The Parkinson Progression Marker Initiative (PPMI). *Prog. Neurobiol.* **95**, 629-635 (2011).

27. Sowa, P., et al. Restriction spectrum imaging of white matter and its relation to neurological disability in multiple sclerosis. *Mult. Scler.* 1352458518765671 (2018).

28. Bustillo, J. R., et al. Glutamatergic and Neuronal Dysfunction in Gray and White Matter: A Spectroscopic Imaging Study in a Large Schizophrenia Sample. *Schizophr. Bull.* **43**, 611-619 (2017).

29. Cetin, M. S., et al. Thalamus and posterior temporal lobe show greater inter-network connectivity at rest and across sensory paradigms in schizophrenia. *Neuroimage* **97**, 117-126 (2014).

30. Gollub, R. L., et al. The MCIC collection: a shared repository of multi-modal, multi-site brain image data from a clinical investigation of schizophrenia. *Neuroinformatics* **11**, 367-388 (2013).

31. Kogan, A., Alpert, K., Ambite, J. L., Marcus, D. S. & Wang, L. Northwestern University schizophrenia data sharing for SchizConnect: A longitudinal dataset for large-scale integration. *Neuroimage* **124**, 1196-1201 (2016).

32. Wang, L., et al. SchizConnect: Mediating neuroimaging databases on schizophrenia and related disorders for large-scale integration. *Neuroimage* **124**, 1155-1167 (2016).

33. Ambite, J. L., et al. SchizConnect: Virtual Data Integration in Neuroimaging. *Data Integr. Life. Sci.* **9162**, 37-51 (2015).

34. Borgwardt, S., et al. Distinguishing prodromal from first-episode psychosis using neuroanatomical single-subject pattern recognition. *Schizophr. Bull.* **39**, 1105-1114 (2013).

35. Dukart, J., et al. Age-related brain structural alterations as an intermediate phenotype of psychosis. *J. Psychiatry Neurosci.* **42**, 307-319 (2017).

36. Dorum, E. S., et al. Age-related differences in brain network activation and co-activation during multiple object tracking. *Brain. Behav.* **6**, e00533 (2016).

37. Kaufmann, T., et al. Task modulations and clinical manifestations in the brain functional connectome in 1615 fMRI datasets. *Neuroimage* **147**, 243-252 (2016).

38. Kaufmann, T., et al. Disintegration of Sensorimotor Brain Networks in Schizophrenia. *Schizophr. Bull.* **41**, 1326-1335 (2015).

39. Skåtun, K. C., et al. Global brain connectivity alterations in patients with schizophrenia and bipolar spectrum disorders. *J. Psychiatry Neurosci.* **41**, 150159 (2016).

40. Brandt, C. L., et al. Cognitive Effort and Schizophrenia Modulate Large-Scale Functional Brain Connectivity. *Schizophr. Bull.* **41**, 1360-1369 (2015).

41. Alfaro-Almagro, F., et al. Image processing and Quality Control for the first 10,000 brain imaging datasets from UK Biobank. *Neuroimage* **166**, 400-424 (2018).

42. Pergola, G., et al. Grey matter volume patterns in thalamic nuclei are associated with familial risk for schizophrenia. *Schizophr. Res.* **180**, 13-20 (2017).
