# Supplementary material for: The genetic architecture of human brainstem structures and their involvement in common brain disorders
Source: Nat Commun. 2020 Aug 11;11:4016. doi: 10.1038/s41467-020-17376-1 (PMC7421944; doi:10.1038/s41467-020-17376-1)
Supplement: Supplementary file 3 — Description of Additional Supplementary Files [file 41467_2020_17376_MOESM3_ESM.docx]

Description of additional supplementary information

Title: Supplementary Data 1

Description: Genetic loci associated with volumes of the whole brainstem, midbrain, pons, superior cerebellar peduncle, and medulla oblongata. Genome-wide associaton studies (GWAS) of 27,034 genotyped participants aged 45-82 years from the UK Biobank identified sixteen genetic loci associated with whole brainstem volume and 10, 23, 3, and 9 loci associated with volumes of the midbrain, pons, SCP, and medulla oblongata, respectively (two-sided P < 5e-8). Thirty of the loci associated with volumes of the individual brainstem regions were not associated with whole brainstem volume. Ten of the loci were only associated with whole brainstem volume, whereas 6, 15, 3, and 3 loci were only significant for midbrain, pons, SCP, and medulla oblongata volumes, respectively. These loci are highlighted in bold below (columns B and C). All GWAS accounted for age, age², sex, scanning site, intracranial volume, genotyping batch, and the first ten genetic principal components to control for population stratification.

Title: Supplementary Data 2

Description: Genetic loci associated with volumes of the midbrain, pons, superior cerebellar peduncle, and medulla oblongata without covarying for whole brainstem volume. Genome-wide associaton studies (GWAS) of 27,034 genotyped healthy individuals aged 40-70 years from the UK Biobank identified 9, 18, 1, and 3 genetic loci associated with volumes of the midbrain, pons, SCP, and medulla oblongata, respectively (two-sided P < 5e-8), when not covarying for whole brainstem volume. All GWAS accounted for age, age², sex, scanning site, intracranial volume, genotyping batch, and the first ten genetic principal components to control for population stratification.

Title: Supplementary Data 3

Description: GWAS results after excluding related individuals. To account for relatedness in the GWAS discovery sample, we excluded all individuals in the sample related up to 4th degree (k = 0.0625 in GCTA; n = 705), leaving us with 26,329 individuals (n = 27,034 before accounting for relatedness). Then, we reran the GWAS for all brainstem volumes in the discovery sample without related individuals. These results show that 1) the large majority of lead SNPs in the original sample are also significant at the genome-wide threshold (P < 5e-8) in the sample with 26,329 individuals and 2) the genetic correlations of the summary statistics before and after excluding related individuals are all near 1. Green color indicates that the lead SNPs identified in the whole sample had a P-value < 5e-8 also in the sample without related individuals, whereas yellow color indicates that the P-values were > 5e-8 in the sample without related individuals. The table shows the results for midbrain, pons, SCP, and medulla oblongata both with (denoted "incl. WBS) and without accounting for whole brainstem volume.

Title: Supplementary Data 4

Description: Genetic loci associated with volumes of the whole brainstem, midbrain, pons, and medulla oblongata (based on independent SNPs significant at the P < 1e-8 threshold). Genome-wide associaton studies (GWAS) of 27,034 genotyped participants aged 45-82 years from the UK Biobank identified thirteen genetic loci associated with whole brainstem volume and 6, 21, and 6 loci associated with volumes of the midbrain, pons, and medulla oblongata, respectively (two-sided P < 1e-8). No locus was significant for SCP. The 46 genomic loci included 34 unique brainstem-associated regions of which 21 were only associated with volumes of the individual brainstem regions and not with whole brainstem volume. All GWAS accounted for age, age², sex, scanning site, intracranial volume, genotyping batch, and the first ten genetic principal components to control for population stratification.

Title: Supplementary Data 5

Description: Functional annotation of single nucleotide polymorphisms (SNPs) in linkage disequilibrium (r2 ≥ 0.6) with one of the independent significant SNPs for whole brainstem volume. We functionally annotated all candidate single-nucleotide polymorphisms (SNPs) that were in linkage disequilibrium (r2 ≥ 0.6) with one of the independent significant SNPs using Functional Mapping and Annotation of GWAS (FUMA). FUMA is based on information from 18 biological repositories and tools and functionally annotates GWAS results. The platform prioritizes the most likely causal SNPs and genes by combining positional, eQTL, and chromatin interaction mapping. FUMA annotates significantly associated SNPs with functional categories, combined CADD scores, RegulomeDB scores, and chromatin states.

Title: Supplementary Data 6

Description: Functional annotation of single nucleotide polymorphisms (SNPs) in linkage disequilibrium (r2 ≥ 0.6) with one of the independent significant SNPs for midbrain volume. We functionally annotated all candidate single-nucleotide polymorphisms (SNPs) that were in linkage disequilibrium (r2 ≥ 0.6) with one of the independent significant SNPs using Functional Mapping and Annotation of GWAS (FUMA). FUMA is based on information from 18 biological repositories and tools and functionally annotates GWAS results. The platform prioritizes the most likely causal SNPs and genes by combining positional, eQTL, and chromatin interaction mapping. FUMA annotates significantly associated SNPs with functional categories, combined CADD scores, RegulomeDB scores, and chromatin states.

Title: Supplementary Data 7

Description: Functional annotation of single nucleotide polymorphisms (SNPs) in linkage disequilibrium (r2 ≥ 0.6) with one of the independent significant SNPs for pons volume. We functionally annotated all candidate single-nucleotide polymorphisms (SNPs) that were in linkage disequilibrium (r2 ≥ 0.6) with one of the independent significant SNPs using Functional Mapping and Annotation of GWAS (FUMA). FUMA is based on information from 18 biological repositories and tools and functionally annotates GWAS results. The platform prioritizes the most likely causal SNPs and genes by combining positional, eQTL, and chromatin interaction mapping. FUMA annotates significantly associated SNPs with functional categories, combined CADD scores, RegulomeDB scores, and chromatin states.

Title: Supplementary Data 8

Description: Functional annotation of single nucleotide polymorphisms (SNPs) in linkage disequilibrium (r2 ≥ 0.6) with one of the independent significant SNPs for superior cerebellar peduncle volume. We functionally annotated all candidate single-nucleotide polymorphisms (SNPs) that were in linkage disequilibrium (r2 ≥ 0.6) with one of the independent significant SNPs using Functional Mapping and Annotation of GWAS (FUMA). FUMA is based on information from 18 biological repositories and tools and functionally annotates GWAS results. The platform prioritizes the most likely causal SNPs and genes by combining positional, eQTL, and chromatin interaction mapping. FUMA annotates significantly associated SNPs with functional categories, combined CADD scores, RegulomeDB scores, and chromatin states.

Title: Supplementary Data 9

Description: Functional annotation of single nucleotide polymorphisms (SNPs) in linkage disequilibrium (r2 ≥ 0.6) with one of the independent significant SNPs for medulla oblongata volume. We functionally annotated all candidate single-nucleotide polymorphisms (SNPs) that were in linkage disequilibrium (r2 ≥ 0.6) with one of the independent significant SNPs using Functional Mapping and Annotation of GWAS (FUMA). FUMA is based on information from 18 biological repositories and tools and functionally annotates GWAS results. The platform prioritizes the most likely causal SNPs and genes by combining positional, eQTL, and chromatin interaction mapping. FUMA annotates significantly associated SNPs with functional categories, combined CADD scores, RegulomeDB scores, and chromatin states.

Title: Supplementary Data 10

Description: Mapping of significant loci from the genome-wide association studies of brainstem volumes to genes. We used positional, expression quantitative trait loci (eQTL), and chromatin interaction mapping in the Functional Mapping and Annotation of GWAS (FUMA) platform to map the 125 independent significant single-nucleotide polymorphisms (with P < 5e-8) in the GWAS discovery sample to genes. These three strategies identified 280 unique genes, where 130, 89, and 181 genes were mapped by positional, eQTL, and chromatin interaction mapping, respectively. 168 of these were implicated by one mapping strategy, 68 genes by two strategies, and 25 of the genes were implicated by three strategies.

Title: Supplementary Data 11

Description: Genome-wide gene-based association analyses of volumes of the whole brainstem, midbrain, pons, superior cerebellar peduncle, and medulla oblongata. Genome-wide gene-based associaton studies (GWGAS; two-sided P < 2.7e-6, i.e., 0.05/18,447 genes) of 27,034 genotyped healthy individuals aged 40-70 years from the UK Biobank identified 87 unique genes across the brainstem regions. Thirty-six genes were associated with whole brainstem volume and 22, 37, 10, and 17 genes were associated with volumes of the midbrain, pons, SCP, and the medulla oblongata, respectively. Twenty-two of the genes were only associated with whole brainstem volume, whereas 13, 14, 6, 5 genes were only significant for midbrain, pons, SCP, and medulla oblongata volumes. These genes are highlighted in bold below. All GWGAS accounted for age, age², sex, scanning site, intracranial volume, genotyping batch, and the first ten genetic principal components to control for population stratification. In addition, the GWGAS for the midbrain, pons, SCP, and medulla oblongata accounted for whole brainstem volume.

Title: Supplementary Data 12

Description: Mapping of significant loci from the genome-wide association studies of brainstem volumes to genes. We used positional, expression quantitative trait loci (eQTL), and chromatin interaction mapping in the Functional Mapping and Annotation of GWAS (FUMA) platform to map the independent significant single-nucleotide polymorphisms with P < 1e-8 in the GWAS discovery sample to genes. These three strategies identified 165 unique genes, where 89, 70, and 93 genes were mapped by positional, eQTL, and chromatin interaction mapping, respectively.

Title: Supplementary Data 13

Description: Genome-wide gene-based association analyses of volumes of the whole brainstem, midbrain, pons, superior cerebellar peduncle, and medulla oblongata. Genome-wide gene-based associaton studies (GWGAS; two-sided P < 5.4e-7, i.e., 0.05/(18,447 genes x 5 volumes)) of 27,034 genotyped healthy individuals aged 40-70 years from the UK Biobank identified 66 unique genes across the brainstem regions. Twenty-seven genes were associated with whole brainstem volume and 12, 33, 7, and 17 genes were associated with volumes of the midbrain, pons, SCP, and the medulla oblongata, respectively. Fifteen of the genes were only associated with whole brainstem volume, whereas 6, 12, 4, and 5 genes were only significant for midbrain, pons, SCP, and medulla oblongata volumes. These genes are highlighted in bold below. All GWGAS accounted for age, age², sex, scanning site, intracranial volume, genotyping batch, and the first ten genetic principal components to control for population stratification. In addition, the GWGAS for the midbrain, pons, SCP, and medulla oblongata accounted for whole brainstem volume.

Title: Supplementary Data 14

Description: Conditional false discovery rate analyses for whole brainstem volume conditioned on eight psychiatric and neurological disorders. We employed conditional false discovery rate (FDR) analyses to uncover more of the genetic underpinnings of whole brainstem volume. These statistical tests were run with an FDR-threshold of 0.01 and are described in more detail in the Methods section. The conditional FDR analyses for whole brainstem volume were run conditioned on each of the eight brain disorders and identified across the disorders 208 significant loci. These were located in 52 unique genetic loci, which are highlighted in bold (at first appearance in the table).

Title: Supplementary Data 15

Description: Conditional false discovery rate analyses for midbrain volume conditioned on eight psychiatric and neurological disorders. We employed conditional false discovery rate (FDR) analyses to uncover more of the genetic underpinnings of midbrain volume. The conditional FDR analyses for midbrain volume were run conditioned on each of the eight brain disorders and identified across the disorders 111 significant loci. These were located in 29 unique genetic loci, which are highlighted in bold (at first appearance in the table).

Title: Supplementary Data 16

Description: Conditional false discovery rate analyses for pons volume conditioned on eight psychiatric and neurological disorders. We employed conditional false discovery rate (FDR) analyses to uncover more of the genetic underpinnings of pons volume. These statistical tests were run with an FDR-threshold of 0.01 and are described in more detail in the Methods section. The conditional FDR analyses for pons volume were run conditioned on each of the eight brain disorders and identified across the disorders 270 significant loci. These were located in 63 unique genetic loci, which are highlighted in bold (at first appearance in the table).

Title: Supplementary Data 17

Description: Conditional false discovery rate analyses for superior cerebellar peduncle volume conditioned on eight psychiatric and neurological disorders. We employed conditional false discovery rate (FDR) analyses to uncover more of the genetic underpinnings of superior cerebellar peduncle (SCP) volume. These statistical tests were run with an FDR-threshold of 0.01 and are described in more detail in the Methods section.The conditional FDR analyses for SCP volume were run conditioned on each of the eight brain disorders and identified across the disorders 55 significant loci. These were located in 21 unique genetic loci, which are highlighted in bold (at first appearance in the table).

Title: Supplementary Data 18

Description: Conditional false discovery rate analyses for medulla oblongata volume conditioned on eight psychiatric and neurological disorders. We employed conditional false discovery rate (FDR) analyses to uncover more of the genetic underpinnings of medulla oblongata volume. These statistical tests were run with an FDR-threshold of 0.01 and are described in more detail in the Methods section. The conditional FDR analyses for medulla oblongata volume were run conditioned on each of the eight brain disorders and identified across the disorders 125 significant loci. These were located in 25 unique genetic loci, which are highlighted in bold (at first appearance in the table).

Title: Supplementary Data 19

Description: Genetic loci shared between brainstem volumes and eight psychiatric and neurological disorders. We performed conjunctional false discovery rate (FDR) analyses to detect genetic loci jointly associated with brainstem volumes and the common brain disorders. These statistical analyses are described in more detail in the Methods section. These analyses revealed shared loci across the brainstem structures and the clinical conditions when applying a conjunctional FDR threshold of 0.05. We found the largest number of loci shared between brainstem volumes and schizophrenia (31), bipolar disorder (14), and Parkinson's disease (17). For autism spectrum disorders, attention deficit hyperactivity disorder, major depression, Alzheimer's disease, and multiple sclerosis there were 9, 4, 6, 5, and 5 genetic loci jointly associated with the brainstem volumes and the disorders, respectively. When using a conjunctional FDR threshold of 0.01 (corrected for 5 volumes), there were genetic loci jointly associated with the brainstem volumes and BD (2), SCZ (10), ASD (3), MS (2), ADHD (1), and PD (6) and no shared loci for MD and AD (column J).
