# Supplementary material for: The genetic architecture of human brainstem structures and their involvement in common brain disorders
Source: Nat Commun. 2020 Aug 11;11:4016. doi: 10.1038/s41467-020-17376-1 (PMC7421944; doi:10.1038/s41467-020-17376-1)
Supplement: Supplementary file 4 — Reporting Summary [file 41467_2020_17376_MOESM4_ESM.pdf]

nature research

Corresponding author(s): Torbjørn Elvsåshagen, Shahram Bahrami, Tobias Kaufmann

Last updated by author(s): Jun 8, 2020

Reporting Summary

Nature Research wishes to improve the reproducibility of the work that we publish. This form provides structure for consistency and transparency in reporting. For further information on Nature Research policies, see Authors & Referees and the Editorial Policy Checklist.

Statistics

For all statistical analyses, confirm that the following items are present in the figure legend, table legend, main text, or Methods section.

n/a	Confirmed	
☐	☒	The exact sample size (*n*) for each experimental group/condition, given as a discrete number and unit of measurement
☐	☒	A statement on whether measurements were taken from distinct samples or whether the same sample was measured repeatedly
☐	☒	The statistical test(s) used AND whether they are one- or two-sided<br>*Only common tests should be described solely by name; describe more complex techniques in the Methods section.*
☐	☒	A description of all covariates tested
☐	☒	A description of any assumptions or corrections, such as tests of normality and adjustment for multiple comparisons
☐	☒	A full description of the statistical parameters including central tendency (e.g. means) or other basic estimates (e.g. regression coefficient) AND variation (e.g. standard deviation) or associated estimates of uncertainty (e.g. confidence intervals)
☐	☒	For null hypothesis testing, the test statistic (e.g. *F*, *t*, *r*) with confidence intervals, effect sizes, degrees of freedom and *P* value noted<br>*Give P values as exact values whenever suitable.*
☒	☐	For Bayesian analysis, information on the choice of priors and Markov chain Monte Carlo settings
☒	☐	For hierarchical and complex designs, identification of the appropriate level for tests and full reporting of outcomes
☐	☒	Estimates of effect sizes (e.g. Cohen's *d*, Pearson's *r*), indicating how they were calculated

Our web collection on statistics for biologists contains articles on many of the points above.

Software and code

Policy information about availability of computer code

Data collection	We collected MRI and genetics data from cohorts of participants with common brain disorders and healthy individuals through collaborations, data sharing platforms, and from in-house samples. Suppl. Tables 1-3 include details for each sample.
Data analysis	- The MRI data was analyzed using Freesurfer 6.0 and Bayesian brainstem segmentation, as described by Iglesias et al (Neuroimage 2015).<br>- Genome-wide association studies were run using PLINK v2.0 and the Functional Mapping and Annotation of GWAS (FUMA) platform v.1.3.5.<br>- Genome-wide gene-based association analyses (GWGAS) were run using MAGMA v1.07 in FUMA v.1.3.5.<br>- conditional and conjunctional FDR analyses were run using in-house custom software, available for download at https://github.com/precimed/pleiofdr, and MATLAB 2017a and Python 3.7.4.<br>- Statistical analyses and figures were generated using custom scripts in R 3.5, which are available from the authors upon request.<br>- LD Score regression v1.0.0 (https://github.com/bulik/ldsc)<br>- Genome-wide complex trait analysis (GCTA) v1.92

For manuscripts utilizing custom algorithms or software that are central to the research but not yet described in published literature, software must be made available to editors/reviewers. We strongly encourage code deposition in a community repository (e.g. GitHub). See the Nature Research guidelines for submitting code & software for further information.

Data

Policy information about availability of data

All manuscripts must include a data availability statement. This statement should provide the following information, where applicable:

- Accession codes, unique identifiers, or web links for publicly available datasets
- A list of figures that have associated raw data
- A description of any restrictions on data availability

Data used in preparation of this article were obtained from the Psychiatric Genomics Consortium (https://www.med.unc.edu/pgc/), 23andMe (https://

October 2018

www.23andme.com/), the International Genomics of Alzheimer's Project (http://web.pasteur-lille.fr/en/recherche/u744/igap/igap_download.php), the International Multiple Sclerosis Genetics Consortium (http://imsgc.net/), and the International Parkinson Disease Genomics Consortium (https://pdgenetics.org/). Data used in preparation of this article were also obtained from the UK Biobank (https://www.ukbiobank.ac.uk/), the Alzheimer's Disease Neuroimaging Initiative (ADNI) database (adni.loni.usc.edu), from ABIDE (http://fcon_1000.projects.nitrc.org/), from ADHD200 (http://fcon_1000.projects.nitrc.org/), OASIS (http://www.oasis-brains.org/), PPMI (http://www.ppmi-info.org/), and SCHIZCONNECT (http://schizconnect.org/). A detailed overview of the included cohorts and acknowledgement of their respective funding sources and cohort-specific details are also provided in Supplementary Table 1. The summary statistics for brainstem volumes of the GWAS discovery sample are available in the Zenodo repository (https://zenodo.org/record/3752700#.Xpb-suSm2bh/). GWAS results are also available on the FUMA website (https://fuma.ctglab.nl/browse/; ID 97-105).

Field-specific reporting

Please select the one below that is the best fit for your research. If you are not sure, read the appropriate sections before making your selection.

☒ Life sciences ☐ Behavioural & social sciences ☐ Ecological, evolutionary & environmental sciences

For a reference copy of the document with all sections, see nature.com/documents/nr-reporting-summary-flat.pdf

Life sciences study design

All studies must disclose on these points even when the disclosure is negative.

Sample size

The present study includes raw T1 MRI data from n = 57,298 individuals. This sample includes 34,466 genotyped participants from the UK Biobank (discovery sample n = 27,034; replication sample n = 7,432). No prior GWAS of individual brainstem regions has been conducted and no formal power analyses or sample size estimations were conducted. We included all patient and healthy control MRI data that was available to us.

Data exclusions

We manually assessed the brain stem segmentations in all MRI data sets by visually inspecting twelve sagittal view figures of the delineations for each participant. This visual quality control (QC) procedure for each data set was conducted blind to case-control status. Data sets were excluded from the study if one of the following requirements was not met: 1. the field of view included the whole brainstem, 2. the superior boundary of the midbrain approximated an axial plane through the mammillary body and the superior edge of the quadrigeminal plate, 3. the boundary between mibrain and pons approximated an axial plane through the superior pontine notch and the inferior edge of the quadrigeminal plate, 4. the boundary between between pons and medulla oblongata approximated an axial plane at the level of the inferior potine notch, 5. the inferior boundary of the medulla oblongata approximated an axial plane at the level of the posterior rim of the foramen magnum, 6. the superior boundary of the SCP approximated the inferior boundary of the midbrain tectum, the inferior boundary of the SCP was defined by the merging with the cerebellum, and the anterior boundary of the SCP was defined by the posterior boundary of the pons, and 7. there were no substantial segmentation errors for the anterior and posterior boundaries of midbrain, pons, and medulla oblongata. This QC procedure excluded 11.4% (n = 6,513) of the data sets, mainly due to insufficient field of view (e.g., not fully covering the inferior part of the medulla oblongata), insufficient data quality, and segmentation errors in the clinical samples, resulting in a final sample size of n = 50,785. These criteria for exclusion were pre-established.

Replication

The brainstem-associated lead SNPs of the discovery sample with P < 5e-8 were also evaluated in a GWAS replication sample. Here, we found that all SNPs had the same effect direction for most of the volumes in the replication sample and that the majority of the lead SNPs had uncorrected P < 0.05 in the GWAS replication sample. Moreover, as expected due to the modest sample size, only two of the lead SNPs reached the P < 5e-8 threshold in the replication sample. Finally, we found that the discovery and replication GWAS for all volumes were significantly genetically correlated (all Rg > 0.73). Due to the modest size of the replication sample and the limited statistical power, there were no attempts at replicating the post-GWAS findings of the present study. These include gene mapping and gene sets analyses, and analyses of genetic overlap between brainstem volumes and common brain disorders. Moreover, for the comparisons of brainstem volumes between individuals with common brain disorders healthy controls, we used all data available to us and no additional data was available for a replication attempt.

Randomization

No randomization was conducted since the study did not include a design e.g., a placebo-controlled treatment trial, where randomization was relevant. The analyses were run on all available data.

Blinding

The visual QC of brainstem segmentations was conducted blind to case-control status.

Reporting for specific materials, systems and methods

We require information from authors about some types of materials, experimental systems and methods used in many studies. Here, indicate whether each material, system or method listed is relevant to your study. If you are not sure if a list item applies to your research, read the appropriate section before selecting a response.

Materials & experimental systems

n/a	Involved in the study
☒	☐ Antibodies
☒	☐ Eukaryotic cell lines
☒	☐ Palaeontology
☒	☐ Animals and other organisms
☐	☒ Human research participants
☒	☐ Clinical data

Methods

n/a	Involved in the study
☒	☐ ChIP-seq
☒	☐ Flow cytometry
☐	☒ MRI-based neuroimaging

Human research participants

Policy information about studies involving human research participants

Population characteristics	The present study includes - after QC and exclusion of data sets - three main samples: 1) 27,034 genotyped individuals from the UK Biobank (GWAS discovery sample; age range 45-82 years); 2) 7,432 additional genotyped individuals from the UK Biobank (GWAS replication sample; age range 50-82 years), and 3) 16,319 individuals with psychiatric or neurological disorders and healthy controls (clinical sample; age range 3-96 years; 5,062 patients and 11,257 healthy controls). The clinical sample includes individuals with the following diagnoses: attention-deficit/hyperactivity disorder (n = 681 patients/n = 992 HC), autism spectrum disorder (n = 125/n = 140), bipolar disorder (n = 464/n = 1,513), major depressive disorder (n = 211/n = 93), schizophrenia (SCZ; n = 1,044/n = 2,079), prodromal SCZ or individuals with increased risk of developing SCZ (SCZRISK; n = 91/n = 402), non-SCZ psychosis spectrum diagnoses (PSYMIX; n = 308/n = 1,430), dementia (n = 756/n = 1,921), mild cognitive impairment (n = 987/n = 1,655), multiple sclerosis (n = 257/n = 1,053), and Parkinson´s disease (n = 138/n = 67). Supplementary Tables 1-3 provide information on the individual cohorts.
Recruitment	Details concerning recruitment procedures can be found in publications for the individual studies, from which the data of the present study was collected, as referenced in Supplementary Table 1. We are not aware of recruitment biases that are likely to have a major impact on the results obtained in the current study.
Ethics oversight	We have collected MRI and genetic data from previously published and/or publicly available data sets, which have received ethical approvals. Details concerning ethical approvals for each data set can be found in the references of each individual study, as provided in Supplementary Table 1.

Note that full information on the approval of the study protocol must also be provided in the manuscript.

Magnetic resonance imaging

Experimental design

Design type	Structural MRI
Design specifications	Detailed information is provided in Supplementary Table 2 and references therein.
Behavioral performance measures	Only structural MRI was used in the present study and no behavioral performance was measured.

Acquisition

Imaging type(s)	Structural MRI, T1-weighted
Field strength	1.5T or 3T, please see Supplementary Table 2 for sample-specific details.
Sequence & imaging parameters	Details concerning sequence and imaging parameters are provided in Supplementary Table 2.
Area of acquisition	Whole brain

Diffusion MRI ☐ Used ☒ Not used

Preprocessing

Preprocessing software	The MRI data was processed using Freesurfer 6.0 (recon -all) and the whole brainstem, midbrain, pons, superior cerebellar peduncle, and medulla oblongata were then delineated using Bayesian brainstem segmentation, as described by Iglesias et al (Neuroimage 2015).
Normalization	Standard procedures employed in Freesurfer (recon -all) were employed.
Normalization template	fsaverage
Noise and artifact removal	We used standard pipelines for anatomical data (Freesurfer recon -all).

Volume censoring

We did not employ volume censoring.

Statistical modeling & inference

Model type and settings

We compared brainstem volumes between individuals with common brain disorders and healthy controls using linear models covarying for sex, age, age², intracranial volume (ICV), and scanner site. The analyses for volumes of midbrain, pons, superior cerebellar peduncle, and medulla oblongata were run both with and without covarying for whole brainstem volume, and were adjusted for multiple testing using FDR (Benjamini-Hochberg, accounting for all 99 tests). Linear models were also run to examine the relationships between the clinical variables and brainstem volumes covarying for sex, age, age², ICV, and scanner site.

Effect(s) tested

We examined the effects of diagnoses (attention-deficit/hyperactivity disorder, autism spectrum disorder, bipolar disorder, major depressive disorder, schizophrenia (SCZ), dementia, mild cognitive impairment (MCI), multiple sclerosis (MS) and Parkinson´s disease (PD)) on volumes of the whole brainstem, midbrain, pons, superior cerebellar peduncle, and medulla oblongata. In addition, information concerning illness severity was available from individuals with MCI, dementia, MS, SCZ, and PD and we examined relationships between the clinical variables and brainstem volumes using linear models. Given differences in sizes of the patient samples, we used Cohen's d as the main statistical outcome, yet also present two-sided P-values and group differences in mm3.

Specify type of analysis: ☐ Whole brain ☒ ROI-based ☐ Both

Anatomical location(s)

The present study analyzed volumes of the whole brainstem, midbrain, pons, superior cerebellar peduncle, and the medulla oblongata.

Statistic type for inference
(See Eklund et al. 2016)

Linear models.

Correction

The GWAS of brainstem volumes and the post-GWAS analyses, including gene mapping, GWGAS, gene sets, and pathways analyses were run both with and without adjustments for analyses of five volumes. The gene sets analyses were also corrected for multiple testing using Bonferroni-correction, whereas the pathway analyses were adjusted for multiple testing using FDR. Conditional and conjunctional FDR-analyses and the brainstem volume group comparisons were adjusted for multiple testing using FDR.

Models & analysis

n/a	Involved in the study
☒	☐ Functional and/or effective connectivity
☒	☐ Graph analysis
☒	☐ Multivariate modeling or predictive analysis
