# Supplementary material for: The genetic architecture of human brainstem structures and their involvement in common brain disorders
Source: Nat Commun. 2020 Aug 11;11:4016. doi: 10.1038/s41467-020-17376-1 (PMC7421944; doi:10.1038/s41467-020-17376-1)
Supplement: Supplementary file 2 — Peer Review File [file 41467_2020_17376_MOESM2_ESM.docx]

Peer Review Information

**Journal:** Nature Communications

**Manuscript title:** The genetic architecture of human brainstem structures and their involvement in common brain disorders

**Corresponding author name(s):** Dr Torbjorn Elvsashagen

**Editorial notes:**

**Transferred manuscripts**

This manuscript has been previously reviewed at another journal that is not operating a transparent peer review scheme. This document only contains reviewer comments and rebuttal letters for versions considered at *Nature Communications*.

**Reviewer comments & decisions:**

**Reviewer comments, first version:**

Reviewer #1 (Remarks to the Author):

Remarks to the authors: 
In "The genetic architecture of human brainstem structures and their involvement in common brain disorders", the authors perform two analyses:
1) a large-scale genome-wide association analysis of human brainstem structures using data from the UK Biobank (UKB) cohort (n=27,034); and
2) phenotype-wide association analysis between human brainstem structures and common brain disorders using additional clinical data from many small datasets (n=16,319 in total).

The authors use Freesurfer software and a Bayesian segmentation algorithm to estimate the volumes of 4 substructures in brainstem, as well as the whole volume of brainstem, for all of the 43,353 individuals. Then they separate the data into two parts to perform the above two different analyses: the first part is the UKB data (n=27,034), and the second part includes 16,319 individuals from all clinical datasets.

Using the UKB data, the authors carry out standard GWAS procedures to estimate SNP-based heritability, identify variants, genes, loci, gene-sets, and explore genetic overlaps using summary statistics. Using the clinical datasets, they perform phenotype-level association analysis with eight common brain disorders.

Overall, this is potentially an interesting and important topic. It is well known that the brainstem has high heritability and may play an important role during brain disorders onset and progression. However, I think there are a couple of major issues in the study design, results presentation, replication, and statistical analysis.


Major comments: 1) There are several recently published GWAS on the whole brainstem volume using the similar UKB data, including Elliott et al. 2018 (mentioned in this paper, n~8,000, Nature), Satizabal et al. 2019 (mentioned in this paper, n=38,851, using both ENIGMA and UKB data, Nature Genetics), and Zhao et al. 2019 (not mentioned in this paper, n~20,000, Nature Genetic). Particularly, the genetic architecture of brain volumes (including the whole brainstem) and their genetic overlaps with neurological traits have been well studied in Satizabal et al. 2019 and Zhao et al. 2019 (https://www.nature.com/ng/volumes/51/issues/11). Given these previous studies, the unique contributions of this study may be limited to the genetic findings for the 4 substructures of brainstem. Thus, when presenting and discussing the results, the authors may what to emphasize more on the unique value of the 4 brainstem substructures: which findings cannot be discovered by using the whole brainstem measurement, but can be uncovered using these 4 new brainstem subfield features? In my opinion, they are the results that can really “increase our knowledge”.

2) One potential statistical issue in the presented GWAS (and post-GWAS) analysis is that the authors did not adjust for testing multiple (five) phenotypes, and thus the expected number of false positives cannot be well controlled. After adjustment, I am afraid that the number of discovered variants/genes/loci might be reduced. At least, similar to Elliott et al. 2018, the author could provide and discuss two versions of their results: 1) adjusted for multiple testing (e.g., 5e-8/5); and 2) not adjusted for multiple testing (e.g., 5e-8).

3) One potential problem in the study design is that there is no validation analysis to elevate the reported novel associations. This issue occurs in both of the UKB genetic analysis (n=27,034) and phenotype-level association analysis (n=16,319). If I understand well, the authors did not try to replicate their results in this paper. The paper may be greatly strengthened if there were some attempts to replicate their findings. This is particularly important because 1) the authors did not adjust for testing multiple phenotypes in their UKB discovery GWAS; and 2) the clinical datasets used in this study are often small and are from various resources, thus, there might be heterogeneities among these studies. Given that fact that the authors have more than 40,000 individuals in their database, I think it could be possible to build a discovery-validation strategy in their study design.

4) Another potential problem is that the paper seems to be a combination of two “independent” analyses on brainstem: the genetic analysis using healthy UKB data, and the phenotype-level analysis using clinical datasets. Although the two analyses study the same phenotype (brainstem), there are few other obvious connections between them.


Minor comments:

1. Page 10, Lines 186-187
The authors write “SNP-based heritability estimated using LD score regression (26) on the GWAS summary statistics was….”
It is not clear to me why the authors did not directly use individual-level data to estimate the SNP heritability by, such as, the GCTA tool (http://cnsgenomics.com/software/gcta/). It is known that the SNP heritability could be underestimated using summary statistics by LDSC (but the genetic correlation will not). Since the individual-level data are available, reporting the SNP heritability estimates by GCTA could be a better choice.
2. Page 10, lines 189-191:
The authors write “We found genome-wide significant hits (P < 5e-8) for all brainstem volumes and identified a total of 125 independent significant SNPs across structures located in 61 genomic loci, using….”
It might be interesting to know how many significant hits/loci we can have after adjusting for multiple phenotypes, i.e., using the P < 5e-8/5 threshold.

Similarly, in the following MAGMA, FUMA, and gene set analyses, the authors may also want to report the results after correcting for multiple testing.

3. Page 10, lines 192-195:
The authors write “Sixteen genetic loci were associated with whole brainstem volume and 10, 23, 3, and 9 loci were associated with volumes of the midbrain, pons, SCP, and medulla oblongata, respectively. Sixteen loci were associated with more than one brainstem volume, thus resulting in 45 unique brainstem-associated genetic regions.”
As mentioned above, it might be interesting to know how many loci/regions are substructure-specific, i.e., they cannot be discovered using whole brainstem volume.

4. Page 21, lines 349-351:
The authors write “Another recently published large-scale GWAS identified 48 genetic loci associated with seven subcortical brain volumes and 16 of these were linked to the whole brainstem...”
Besides this paper (Ref46), there is another recent large-scale study using the similar UKB dataset (Zhao et al., https://www.ncbi.nlm.nih.gov/pubmed/31676860), which is published in the same issue of Nature Genetics. It might be worth to compare the results of current study to those in Zhao et al.


Reviewer #2 (Remarks to the Author):

Using imaging genetics data of 27,034 individuals the authors identified 45 genetic loci associated with volume of midbrain, pons, and medulla oblongata and identified 305 candidate genes within these loci. Next, they assessed polygenic overlap between brainstem volumes and eight brain disorders: attention deficit hyperactivity disorder (ADHD), Autism spectrum disorder (ASD), Schizophrenia (SZ), and Bipolar disorder (BD) major depression (MD), Alzheimer’s disease (AD), multiple sclerosis (MS), and Parkinson disease (PD) using data of 16,319 individuals and found polygenic overlap between volumes and the different disorders. These findings suggest that brainstem regions play a role in these disorders.

Besides two previous study identifying genetic risk loci for the volume of the whole brainstem (Elliott, L. T. et al. Genome-wide association studies of brain imaging phenotypes in UK Biobank. Nature 2018;562, 210-2016; Satizabal, C. L. et al. Genetic architecture of subcortical brain structures in 38,851 individuals. Nature Genet 2018: 10.1038/s41588-019-0511-y) no study has assessed genetic risk loci for volume of different regions of the brainstem before. It provides further insight into the genetic architecture of the different regions of the brainstem.

This is well conducted study including large sets of MRI and genetic data of both healthy individuals and patients with different types of brain disease.

Given the utility of GWAS in drug discovery and drug repositioning, It would interesting to know whether using these data genetic associations of known drug targets can be generated (for example using Drug Targetor of other tools).


Reviewer #3 (Remarks to the Author):

In their manuscript “The genetic architecture of human brainstem structures and their involvement in common brain disorders” the authors investigate the genetic architecture of brainstem structures based on T1-weighted MRI data using a Bayesian approach with subsequent genome-wide association studies (GWASs) in the UK Biobank. In a second sample, the researchers investigate differences in brain structures between individuals with psychiatric disorders and healthy controls. The paper is in important contribution to the field; however, certain points are unclear and in general I find the discussion difficult to follow. The following issues remain:

1) Currently, it is difficult to understand how the samples are combined. I have inspected table 3. However, it is still not clear to me. It looks like the GWASs analyses were only performed in the UK Biobank sample, while the regression analyses were performed in the other ascertained samples? The abstract states that clinical data of “16,319 individuals” was investigated; however, the number per phenotype is far smaller. Could you please clarify this?

2) It is difficult to judge the quality of the performed GWASs as only the top five region plots are presented in the supplement. Furthermore, neither p values nor SEs of the LDSC-based heritability estimates, nor LDSC intercept, nor attenuation ratio are presented which would allow to judge if it has sufficiently been adjusted for potential population stratification. If the GWAS have been uploaded in FUMA, why are the results not publicly available or made at least available for review to inspect region plots?

3) I can’t see from the methods section which settings and which reference panel has been used in FUMA. Please, specify this.

4) FUMA in general performs two subsequent clumping procedures and FUMA draws a distinction between "independent significant SNPs" and "lead SNPs". If I recall correctly, it performs a second clumping step with r2 = 0.1. Please, could you clarify this in the methods/results section.

5) On page 10, you state that the SNPs had minimum chromatin state of 1-7. Could you please add more information/explanation what is meant by this?

6) Looking at supplementary table 15, I am wondering about the results of this analysis. The identified pathways do not seem to tag underlying biology of the brain stem (which additionally makes me wonder about the GWAS). Given the multiple analysis performed, I would recommend performing a more conservative multiple testing correction, such as Bonferroni (even though the pathways are not independent) for both pathway analysis: MAGMA and ConsensusPathDB. Many results seem to lie around a Q value of 0.05. Alternatively, the authors could present both, FDR-adjusted Q values and a Bonferroni-corrected p value threshold. Then the reader could judge for themselves which results are more plausible.

7) It would be interesting to see how the GWASs of brain stem structures correlate with other GWAS of any brain structure. You discuss that the LDSC-based genetic correlations between brain stem structures and psychiatric/neurological disorders may not be significant due to effect sizes mainly in opposite directions and therefore chose an alternative approach using stratified QQplots. However, that none of the GWASs shows any genetic correlation with any of the psychiatric disorders is a little surprising. It would be good to perform more genetic correlation analysis (LDSC, LDAK, or polygenic score analyses) to see if these GWASs of brain stem structures show any genetic relationship/overlap with any other brain phenotypes, such as brain structure, diseases, or disorders.

8) Furthermore, it would add to the paper if you could–maybe–graphically investigate the discussion point that you make about the “opposite effect sizes” in the brain stem structure and neuropsychiatric disorder GWASs. If this assumption is true, the effect sizes of the brain stem structures plotted against the psychiatric disorder effect sizes should mainly present as a cloud with no obvious (correlation) structure in the data. Please, consider adding this to the supplement?

9) I read the original publications introducing the conditional and conjunctional FDR method to investigate potential pleiotropy. As my Bayesian knowledge is limited, I find them quite difficult to understand. I would appreciate if you could add more explanation of these methods rather than the references and the short explanation on pages 64 and 65. It would be helpful if it included an explanation why this leads to an increase in power to detect more significant associations after the summary statistics “have been combined” (page 15, line 267 and pages 64 and 65).

10) Is the conditional FDR method similar to GenomicSEM? If so, I would recommend that you compare results from both approaches to test for robustness of your findings.

11) Code availability. I highly recommend the authors to make their code available on github or any other sharing platform. Especially, regarding the conditional and conjunctional FDR methods. This would also facilitate the understanding of the method. It would be very important for other researchers to be able to perform these analyses with other traits. Especially, if it holds true, that this method can measure pleiotropy that cannot be picked up by LDSC-based or LDAK-based genetic correlations.

12) The second phenotypic investigation feels currently somehow disconnected from the GWAS part in the UK Biobank. Are these participants not genotyped? Would it be possible to perform polygenic score analysis with the GWAS that were obtained from the UK Biobank in these samples?

13) Figure 5. Why are the effect sizes standardised? If these are brain volumes, they are potentially measured in cm3 or any other volume measure? It would be interesting to see how big the actual difference in volume between cases and controls is adjusting for the mentioned covariates. This would allow the reader to judge actually clinical differences. Relating to this, I think it would add to the manuscript if you could present averages/scatterplots/boxplots of the brain stem volumes for healthy and affected individuals per disorder?

14) Looking at the quality control of the GWASs in the UK Biobank, I cannot find how relatedness in the sample has been handled. Could you please clarify this?

15) Overall, I found the discussion difficult to follow. Especially, as it puts so much emphasis on discussing certain genes that have been associated. They may be interesting candidates; however, this is only based on closest vicinity of a lead SNP, gene-based analyses, or gene-set analysis. I would recommend cutting down on this rather speculative part of the discussion.

**Author rebuttal, first version:**

***Reviewer #1***

***Overall, this is potentially an interesting and important topic. It is well known that the brainstem has high heritability and may play an important role during brain disorders onset and progression. However, I think there are a couple of major issues in the study design, results presentation, replication, and statistical analysis.***

We thank the reviewer for the comments and constructive suggestions, which have clearly helped us improve the manuscript. The issues that were raised by the reviewer have been addressed, as outlined below.

Major comments:

***1) … Given these previous studies, the unique contributions of this study may be limited to the genetic findings for the 4 substructures of brainstem. Thus, when presenting and discussing the results, the authors may what to emphasize more on the unique value of the 4 brainstem substructures: which findings cannot be discovered by using the whole brainstem
measurement, but can be uncovered using these 4 new brainstem subfield features? In my opinion, they are the results that can really “increase our knowledge”.***

We thank the reviewer for pointing this out and have added the following sentence to the Introduction section on p. 7, to further highlight the three recent GWAS of the whole brainstem:

*“Recent imaging-genetics studies detected the first genetic loci linked to whole brainstem volume^20-22^, yet no large-scale neuroimaging study has focused on the differential genetic architecture of the midbrain, pons, and medulla oblongata and their involvement in common brain disorders.”*

We have also in the Results and Discussion sections emphasized the findings for the four brainstem structures, as suggested by the Reviewer. To the Results section on p. 10-11, we added the following sentences:

*“Thirty of these unique loci were associated with volumes of the individual brainstem regions and not with whole brainstem volume. Moreover, 6, 15, 3, and 3 loci were only significant for midbrain, pons, SCP, and medulla oblongata volumes, respectively, whereas 10 of the loci were only associated with whole brainstem volume (Supplementary Table 5)”.*

To the Results section on p. 11 (where we now describe results when adjusting for analyses of five volumes), we added the following sentence:

*“The 46 genomic loci included 34 unique brainstem-associated regions of which 21 were only associated with volumes of the individual brainstem regions and not with whole brainstem volume.”*

When it comes to the descriptions of associated genes and gene sets for each brainstem subregion, these were also included in the original manuscript and can now be found on p. 14 and 16.

In accordance with the reviewer’s recommendation, we have rewritten larger parts of the Discussion and now focus on genetic findings per brainstem subregion (p. 25 and 26).

***2) One potential statistical issue in the presented GWAS (and post-GWAS) analysis is that the authors did not adjust for testing multiple (five) phenotypes, and thus the expected number of false positives cannot be well controlled. After adjustment, I am afraid that the number of discovered variants/genes/loci might be reduced. At least, similar to Elliott et al. 2018, the author could provide and discuss two versions of their results: 1) adjusted for multiple testing (e.g., 5e-8/5); and 2) not adjusted for multiple testing (e.g., 5e-8).***

The reviewer raised an important point. We agree and have now adjusted the results for testing five brainstem volumes. In the revised manuscript, we have specified findings significant at the *P* < 5e-8 and the *P* < 1e-8 thresholds in the figures, Results and Discussion sections, thereby discussing two versions similar to Elliott et al 2018, and in line with the reviewer’s suggestion.

Concerning the GWAS loci, we added the following to the Results section on p. 11:

*“When employing a more stringent statistical threshold of P < 1e-8 (corrected for analyses of 5 volumes), there were 46 significant genetic loci across the brainstem structures (Supplementary Table 8). Thirteen of these loci were associated with whole brainstem volume and 6, 21, and 6 loci were associated with volumes of the midbrain, pons, and medulla oblongata, respectively. No locus was significant for SCP at this statistical threshold. The 46 genomic loci included 34 unique brainstem-associated regions of which 21 were only associated with volumes of the individual brainstem regions and not with whole brainstem volume.*”

Concerning the mapped genes, we added the following to the Results section on p. 14-15:

*“We also mapped independent SNPs significant at the P < 1e-8 threshold (corrected for analyses of 5 volumes) by positional, eQTL, and chromatin interaction mapping and identified 96, 57, and 100 genes, respectively (Supplementary Table 18). GWGAS detected 66 unique genes across the brainstem volumes when applying a significance threshold of P < 5.4e-7 (i.e., 2.7e-6/5 volumes; Supplementary Fig. 9 and Supplementary Table 19). Fifteen were only associated with whole brainstem volume, whereas 6, 12, 4, and 5 genes were only significant for midbrain, pons, SCP, and medulla oblongata volumes, respectively.”*

Concerning the implicated gene sets, we added the following to the Results section on p. 16:

*“The gene sets analyses and the pathway analyses were also run with prioritized genes from the discovery GWAS sample when adjusting for five volumes (based on independent significant SNPs with P < 1e-8) and implicated the same gene sets and pathways as in the main analysis (based on independent significant SNPs with P < 5e-8). Twelve Gene Ontology gene sets were associated with whole brainstem volume, and 1, 7, and 17 gene sets were associated with volumes of the midbrain, pons, and medulla oblongata, respectively (Supplementary Table 22). ConsensusPathDB^32^ identified 53, 1, 25, and 58 significant pathways for volumes of the whole brainstem, pons, superior cerebellar peduncle, and medulla oblongata, respectively (Supplementary Table 23).*”

In the Discussion, we specified numbers of loci that were significant at the *P* < 5e-8 and the *P* < 1e-8 thresholds on p. 24:

*“We identified 61 genetic loci associated with brainstem volumes at a statistical threshold of P < 5e-8. Sixteen of the loci were associated with more than one volume, thus resulting in 45 unique brainstem-associated genetic regions. Sixteen of these were associated with whole brainstem volume and 30 of them were associated with volumes of the individual brainstem regions and not with whole brainstem volume. Thirty-four of the 45 unique loci remained significant at a threshold of P < 1e-8, i.e., adjusted for analyses of five volumes.”*

We also added the following Supplementary Fig. 9 to the revision, which shows the genes detected by GWGAS at a corrected threshold (i.e., 2.7e-6/5 volumes):

***3) … The paper may be greatly strengthened if there were some attempts to replicate their findings. This is particularly important because 1) the authors did not adjust for testing multiple phenotypes in their UKB discovery GWAS; and 2) the clinical datasets used in this study are often small and are from various resources, thus, there might be heterogeneities among these studies. Given that fact that the authors have more than 40,000 individuals in their database, I think it could be possible to build a discovery-validation strategy in their study design.***

We thank the reviewer for the comment and the revised manuscript now includes a replication sample of an additional 7,432 genotyped individuals from the UK Biobank. All of these datasets underwent the same QC procedures as the original GWAS sample (*n* = 27,034, now referred to as the “GWAS discovery sample” in the manuscript). We also acknowledge that it was not entirely clear from the original manuscript version that we – with few exceptions – did not have genetic data from the clinical samples. If the clinical samples had been genotyped, we could have used the clinical controls as a replication sample for the GWAS results. We have clarified this detail in the manuscript.

Due to the modest size of the replication sample (*n* = 7,432), the probability of replicating GWAS findings at the *P* < 5e-8 threshold was low. Therefore, we first examined whether lead SNPs with *P* < 5e-8 in the GWAS discovery sample had the same directions of effect in the replication sample. We found that all SNPs had the same effect direction for most of the volumes (all but a few SNPs for medulla oblongata and midbrain volumes when accounting for whole brainstem volume). We then examined whether the lead SNPs with *P* < 5e-8 in the GWAS discovery sample had uncorrected *P* < 0.05 in the replication sample. For most volumes, the majority of the lead SNPs had uncorrected *P* < 0.05 in the GWAS replication sample. Moreover, we examined whether the lead SNPs with *P* < 5e-8 in the GWAS discovery sample had *P* < 5e-8 in the replication sample and found, in line with expectations given the limited sample size, that only two lead SNPs associated with pons volume reached this threshold in the replication. Finally, we found that the discovery and replication GWAS for all volumes were significantly genetically correlated.

These findings have been added to a new Supplementary Table 9 and to the Results section on p. 11:

***“Further evidence of association in the replication GWAS.*** *The brainstem-associated lead SNPs of the discovery sample with P < 5e-8 were further evaluated in the replication GWAS (n = 7,432). We found that all SNPs had the same effect direction for most of the volumes (all but a few SNPs for medulla oblongata and midbrain volumes when accounting for whole brainstem volume; Supplementary Table 9). Next, we found that for most volumes, the majority of the lead SNPs had uncorrected P < 0.05 in the GWAS replication sample. Moreover, as expected due to the modest sample size, only two of the lead SNPs reached the P < 5e-8 threshold in the replication sample (both associated with pons volume). Finally, we found that the discovery and replication GWAS for all volumes were significantly genetically correlated (all R_g_ > 0.73; Supplementary Table 9).*

We also added the following sentence to the Abstract on p. 6:

*“In a replication sample of 7,432 participants most of the loci showed the same effect direction and were significant at a nominal threshold.”*

***4) Another potential problem is that the paper seems to be a combination of two “independent” analyses on brainstem: the genetic analysis using healthy UKB data, and the phenotype-level analysis using clinical datasets. Although the two analyses study the same phenotype (brainstem), there are few other obvious connections between them.***

We thank the reviewer for pointing this out. Before we submitted the original manuscript, we discussed how we could further connect the genetic analyses of the UK Biobank data with the clinical data. We felt that we had to be very careful when discussing how the genetic and clinical volumetric findings could be related since the numbers of genetic loci shared between brainstem volumes and each disorder were modest. Moreover, we did not – with few exceptions – have genetic data from the clinical samples. However, we acknowledge now that the potential links between the genetic and the phenotype-level volumetric data might have been insufficiently discussed in the first manuscript version. Thus, although we still prefer to be careful when discussing potential links between genetics and volumetric alterations, we have added the text to the revised manuscript, which we hope binds the two analyses better together. We added the following to the Results section on p. 19:

*“No definitive conclusions can be drawn about effect directions for each disorder due to the modest number of shared loci, yet the majority of loci for SCZ showed opposite effect directions (74%; i.e., disorder-linked variants were associated with reduced volumes), whereas the majority of loci for PD showed same effect directions (70%; i.e., disorder-linked variants were associated with increased volumes).”*

To the Discussion on p. 28-29, we added the following:

*“We do however note that a majority of the genetic loci shared between SCZ and brainstem volumes in the current study had opposite effects directions, i.e., disorder-linked variants were associated with reduced volumes. This finding is suggestive of genetic contributions to brainstem volume decreases in SCZ.” […]*

*“Furthermore, the majority of loci jointly associated with PD and brainstem volumes in the present study showed same effect directions, i.e., disorder-linked variants were associated with increased volumes. Although no definitive conclusions can be drawn due to the modest number of shared loci, this may suggest that brainstem volume increases in PD, if present, may be at least partly genetically mediated.”*

Minor comments:

***1. … It is known that the SNP heritability could be underestimated using summary statistics by LDSC (but the genetic correlation will not). Since the individual-level data are available, reporting the SNP heritability estimates by GCTA could be a better choice.***

We are grateful for the comment and have estimated SNP-based heritability by GCTA. As reviewer #1 suggested, we found substantially higher heritability estimates with GCTA than with LDSR across the brainstem volumes. Thus, we present the GCTA-based heritability estimates as the main finding in the revised manuscript, including in Figure 1a and in a new Supplementary Table 4. For completeness, we also show LDSR estimates with more details in Supplementary Table 4. In addition, we changed the text concerning SNP-based heritability estimates in the Results section (on p. 10) to:

"*SNP-based heritability estimated using genome-wide complex trait analysis (GCTA)**^29^* *on the GWAS summary statistics was 48% for the whole brainstem, 47% for the midbrain, 47% for pons, 27% for SCP, and 35% for the medulla oblongata (all s.e. < 5%; all P < 1e-16), illustrating the substantial genetic influence on brainstem volumes (Fig. 1a and Supplementary Table 4).*“

***2. … It might be interesting to know how many significant hits/loci we can have after adjusting for multiple phenotypes, i.e., using the P < 5e-8/5 threshold.
Similarly, in the following MAGMA, FUMA, and gene set analyses, the authors may also want to report the results after correcting for multiple testing.***

Please, see the response to Major comment #2 above, where we describe the addition of results when applying the *P* < 1e-8 threshold.

***3. The authors write “Sixteen genetic loci were associated with whole brainstem volume and 10, 23, 3, and 9 loci were associated with volumes of the midbrain, pons, SCP, and medulla oblongata, respectively. Sixteen loci were associated with more than one brainstem volume, thus resulting in 45 unique brainstem-associated genetic regions.” As mentioned above, it might be interesting to know how many loci/regions are substructure-specific, i.e., they cannot be discovered using whole brainstem volume.***

We thank the reviewer for pointing this out. We have added text describing the number of loci that where substructure-specific. To the Results section on p. 10-11, we added the following sentence:

*“Thirty of these unique loci were associated with volumes of the individual brainstem regions and not with whole brainstem volume.”*

To the Results section on p. 11 (where we now describe results when adjusting for analyses of five volumes), we added the following sentence:

*“The 46 genomic loci included 34 unique brainstem-associated regions of which 21 were only associated with volumes of the individual brainstem regions and not with whole brainstem volume.”*

4. ***The authors write “Another recently published large-scale GWAS identified 48 genetic loci associated with seven subcortical brain volumes and 16 of these were linked to the whole brainstem...” Besides this paper (Ref46), there is another recent large-scale study using the similar UKB dataset (Zhao et al.,*** [***https://www.ncbi.nlm.nih.gov/pubmed/31676860***](https://www.ncbi.nlm.nih.gov/pubmed/31676860)***), which is published in the same issue of Nature Genetics. It might be worth to compare the results of current study to those in Zhao et al.***

We thank the reviewer for pointing this out. The paper from Zhao and colleagues published in Nature Genetics 2019 are now included as one of the citations in the Introduction:

“*Recent imaging-genetics studies detected the first genetic loci linked to whole brainstem volume^20-22^, …*”

In addition, we compare our findings with those of Zhao and colleagues in the revised Discussion on p. 24-25:

*“Another recent GWAS of 101 brain phenotypes detected 6 and 15 genetic loci associated with whole brainstem volume at thresholds of P < 4.9e-10 (adjusted for analyses of 101 phenotypes) and P < 5e-8, respectively**^19^* *and 9 of the latter are within regions associated with whole brainstem volume in the present study.”*

***Reviewer #2***

***This is well conducted study including large sets of MRI and genetic data of both healthy individuals and patients with different types of brain disease.***

We thank the reviewer, and are very grateful for the positive comments.

***Given the utility of GWAS in drug discovery and drug repositioning, It would interesting to know whether using these data genetic associations of known drug targets can be generated (for example using Drug Targetor of other tools).***

We agree with the reviewer that it will be highly interesting to see if the GWAS results of the present study can be used in drug discovery and drug repositioning. Thus, we have now looked at tools such as Drug Targetor that could address these questions. However, we and others at our research centre have no prior experience within this field of research and feel that these questions would be better addressed in a future study focusing on drug discovery and repositioning. We have now made the GWAS summary statistics (<https://zenodo.org/record/3752700#.Xpb-suSm2bh>) and FUMA results (<https://fuma.ctglab.nl/browse>; ID 97-105) publicly available to ensure easy uptake of our results by other researchers, including those focused on drug discovery and repositioning.

***Reviewer #3***

We are very grateful for the reviewers’ evaluation, which clearly helped us improve the manuscript.

***1) Currently, it is difficult to understand how the samples are combined. I have inspected table 3. However, it is still not clear to me. It looks like the GWASs analyses were only performed in the UK Biobank sample, while the regression analyses were performed in the other ascertained samples? The abstract states that clinical data of “16,319 individuals” was investigated; however, the number per phenotype is far smaller. Could you please clarify this?***

We thank the reviewer for pointing out the lack of clarity in our previous descriptions. We have clarified in the revised manuscript that three samples were included: 1) GWAS discovery sample with participants from UK Biobank, 2) GWAS replication sample with additional participants from UK Biobank (added to this revision), and 3) clinical sample with patients with neurological or psychiatric disorders and healthy controls. In addition, we have made it clear that genetic analyses were only conducted in the GWAS discovery and replication samples, since genetic data was – with few exceptions – not available in the clinical sample. Thus, we have added the following to the Results section on p. 9:

*“The resulting 50,785 MRI data sets comprised three main samples (Supplementary Table 3): 1) 27,034 genotyped individuals from the UK Biobank (GWAS discovery sample; age range 45.1-81.5 years); 2) 7,432 additional genotyped individuals from the UK Biobank (GWAS replication sample; age range 49.5-82.1 years), and 3) 16,319 individuals with psychiatric or neurological disorders and healthy controls (clinical sample; age range 3-96 years; 5,062 patients and 11,257 controls). Only a small minority in the clinical sample had genotype data and no genetic analyses were run for these individuals.”*

In addition, we have specified in the Abstract the numbers of patients and controls within the clinical sample:

*“In additional clinical data from 5,062 individuals with common brain disorders and 11,257 healthy controls, we observed differential volume alterations in schizophrenia, bipolar disorder, multiple sclerosis, mild cognitive impairment, dementia, and Parkinson’s disease, supporting the relevance of brainstem regions and their genetic architectures in common brain disorders”.*

Moreover, we have updated Supplementary Table 3 to clarify that we now have three main samples.

Furthermore, for this study, we included all data that were available to us through collaborations and databases. Therefore, there is substantial variation in numbers of individuals with each clinical condition. Moreover, for each clinical condition, we included all healthy individuals that were imaged on the same scanners as the patients they were compared with. We detail this in the Methods section on p. 65:

“*We included all healthy individuals that were imaged on the same scanners as the patients they were compared with, in the respective control groups. For clinical conditions where participants were imaged on multiple scanners, we included scanner site as a covariate in the analyses.*”

We believe that the reporting of the samples and respective analyses is now clearer and thank the reviewer for pointing us to this room for improvement.

***2) It is difficult to judge the quality of the performed GWASs as only the top five region plots are presented in the supplement. Furthermore, neither p values nor SEs of the LDSC-based heritability estimates, nor LDSC intercept, nor attenuation ratio are presented which would allow to judge if it has sufficiently been adjusted for potential population stratification. If the GWAS have been uploaded in FUMA, why are the results not publicly available or made at least available for review to inspect region plots?***

We thank the reviewer for pointing this out. The GWASs are now publicly available at https://zenodo.org/record/3752700#.Xpb-suSm2bh and corresponding FUMA results are available at <https://fuma.ctglab.nl/browse> (FUMA ID 97-105). In addition, we have added the *P*-values, SEs, intercept and attenuation ratio for the LDSC-based heritability estimates to Supplementary Table 4. Furthermore, and in line with the recommendation of reviewer #1 (minor comment 1), we now present GCTA-based heritability estimates in the main text. Please, see also response to Minor comment 1 of Reviewer 1. For completeness and transparency, we provide full details from both GCTA- and LDSC-based analyses side-by-side in Supplementary Table 4.

***3) I can’t see from the methods section which settings and which reference panel has been used in FUMA. Please, specify this.***

We thank the reviewer for the comment. The analyses run in FUMA and the applied settings are now publicly available on the FUMA website. For this work, we used the 1000GPhase3 EUR as the reference panel for computing r^2^ and MAF. We added the following to the Methods section on p. 62:

*“The settings and results of the FUMA analyses can be found at* [*https://fuma.ctglab.nl/browse*](https://fuma.ctglab.nl/browse) *(FUMA ID 97-105). For these analyses, we used the 1000GPhase3 EUR as reference panel […]”*

***4) FUMA in general performs two subsequent clumping procedures and FUMA draws a distinction between "independent significant SNPs" and "lead SNPs". If I recall correctly, it performs a second clumping step with r2 = 0.1. Please, could you clarify this in the methods/results section.***

We thank the Reviewer for pointing to the lack of detail and we have now added the following concerning the two subsequent clumping procedures to the Methods section on p. 62:

*“The minimum r^2^ was used to determine the borders of the genomic risk loci and during the first clumping step, the SNPs that were at r^2^ ≤ 0.6 with each other within a 1 mb window were considered as independent significant SNPs. In the next step, independent significant SNPs with r^2^ < 0.1 within a 1 mb window were defined as lead SNPs.”*

***5) On page 10, you state that the SNPs had minimum chromatin state of 1-7. Could you please add more information/explanation what is meant by this?***

We agree that this statement was lacking sufficient details and have added the following information concerning chromatin state to the Methods section on p. 63:

*“The chromatin state shows a genomic region’s accessibility for every 200bp with 15 categorical*

*states predicted by ChromHMM based on five histone modification marks (H3K4me3,*

*H3K4me1, H3K36me3, H3K27me3, H3K9me3) for all 127 epigenomes^31^.*

*A lower score shows higher accessibility in the chromatin state and refers to a more open state.*

*The 15-core chromatin states as suggested by Roadmap are as follows: 1=Active Transcription*

*Start Site (TSS); 2=Flanking Active TSS; 3=Transcription at gene 5’ and 3’; 4=Strong*

*transcription; 5=Weak Transcription; 6=Genic enhancers; 7=Enhancers; 8=Zinc finger genes &*

*repeats; 9=Heterochromatic; 10=Bivalent/Poised TSS; 11=Flanking Bivalent/Poised TSS/Enh;*

*12=Bivalent Enhancer; 13=Repressed PolyComb; 14=Weak Repressed PolyComb;*

*15=Quiescent/Low^78^.”*

***6) Given the multiple analysis performed, I would recommend performing a more conservative multiple testing correction, such as Bonferroni (even though the pathways are not independent) for both pathway analysis: MAGMA and ConsensusPathDB. Many results seem to lie around a Q value of 0.05. Alternatively, the authors could present both, FDR-adjusted Q values and a Bonferroni-corrected p value threshold. Then the reader could judge for themselves which results are more plausible.***

The reviewer raises an important point and we agree that adding results from a more stringent multiple comparison correction will be highly valuable. This was also brought to our attention by Reviewer #1 (major comment #2), who suggested that we should discuss results with and without correction for five volumes. Accordingly, we now also present all results, including the gene sets and pathway analyses, when adjusting for five volumes in the revised manuscript (i.e., based on independent significant SNPs with *P* < 1e-8). In addition, as suggested by Reviewer #3, we have applied Bonferroni-correction to the gene sets analyses, thus adding another layer of stringency. Only in the case of the pathway analyses we have kept the internal FDR-correction procedure rather than adding a Bonferroni correction as this is the default output of the analysis pipeline. To conclude, the revised pipelines now all correct for five analyses performed (5 volumes) and on top of this implement a more stringent correction for the gene set analysis. Overall, although these analyses show fewer significant findings, they implicate the same gene sets and pathways as in the original manuscript version, e.g., those involving *HOX* genes, supporting the robustness of our report. Accordingly, we have made changes to the Results section on p. 15-16, where we state:

*“We conducted gene sets analyses for the genes prioritized in the GWAS discovery sample (based on independent significant SNPs with P < 5e-8) and identified 7 Gene Ontology sets significantly associated with whole brainstem volume, and 2, 8, 1, and 15 gene sets associated with volumes of the midbrain, pons, SCP, and medulla oblongata, respectively, after Bonferroni correction (Supplementary Table 20)*”.

and

“*The most significant gene set for whole brainstem volume was susceptibility to natural killer cell mediated cytotoxicity (P = 1.32e-6), amyloid beta formation for midbrain (P = 0.01), skeletal system morphogenesis for pons (P = 4.6e-5), de novo imp biosynthetic process for SCP (P = 0.03), and embryonic skeletal system development for medulla oblongata (P = 1.0e-7). Notably, HOX genes, which encode transcription factors with central roles in nervous system development**^31^* *were included in 7 of the significant gene sets for pons and in all gene sets associated with medulla oblongata. We also employed the ConsensusPathDB^32^ to identify over-represented pathways for the mapped genes and found 13 significant pathways for whole brainstem volume, and 1, 25, and 58 signficant for pathways for pons, SCP, and medulla oblongata volume, after FDR-correction.*”

and

*“The gene sets analyses and the pathway analyses were also run with prioritized genes from the discovery GWAS sample when adjusting for five volumes (based on independent significant SNPs with P < 1e-8) and implicated the same gene sets and pathways as in the main analysis (based on independent significant SNPs with P < 5e-8). Twelve Gene Ontology gene sets were associated with whole brainstem volume, and 1, 7, and 17 gene sets were associated with volumes of the midbrain, pons, and medulla oblongata, respectively (Supplementary Table 22). ConsensusPathDB^32^ identified 53, 1, 25, and 58 significant pathways for volumes of the whole brainstem, pons, superior cerebellar peduncle, and medulla oblongata, respectively (Supplementary Table 23).”*

***7) It would be good to perform more genetic correlation analysis (LDSC, LDAK, or polygenic score analyses) to see if these GWASs of brain stem structures show any genetic relationship/overlap with any other brain phenotypes, such as brain structure, diseases, or disorders.***

We agree with the reviewer that there are many additional facets that will be of interest to study. Brainstem regions are also implicated in the regulation of cardiovascular function, energy balance, sleep-wake cycle, cognition, and emotions and we are currently working on a dedicated study that explores the genetic relationship between traits related to these functions and brainstem regions. In this study we are investigating the relationship between brainstem regions and coronary artery disease, systolic and diastolic blood pressure, body mass index (BMI), sleep duration, intelligence, educational attainment, and neuroticism. After correcting for multiple testing, we find significant negative correlation between midbrain volume and coronary artery disease (*R*g = -0.17, *P* = 0.00008) and a positive association between midbrain volume and educational attainment (*R*g = 0.11, *P* = 0.0003). However, the focus of our current manuscript lies on eight neurological and psychiatric disorders, where we also have clinical data from individuals with the disorders available that accompany our genetics analysis. Adding the entire analysis of the study mentioned above and further clinical data for these conditions to back up the genetic findings in line with what we do for the current eight disorders would largely exceed the manuscript volume and decrease its focus. Thus, we prefer to assess other conditions both genetically and clinically in a dedicated manuscript.

***8) Furthermore, it would add to the paper if you could–maybe–graphically investigate the discussion point that you make about the “opposite effect sizes” in the brain stem structure and neuropsychiatric disorder GWASs. If this assumption is true, the effect sizes of the brain stem structures plotted against the psychiatric disorder effect sizes should mainly present as a cloud with no obvious (correlation) structure in the data. Please, consider adding this to the supplement?***

We thank the reviewer for the constructive suggestion to add a graphical illustration of the opposite effect directions. We have added a new Supplementary Fig. 12 (for convenience pasted below) to the revised manuscript that shows effect directions (z-scores) for the lead SNPs shared between the brainstem volumes and the eight brain disorders. We show the plots both for all disorders combined and for each disorder separately and indicate same effect directions in red colors (i.e., disorder-linked variants were associated with increased volumes) and opposite effect directions in blue colors (i.e., disorder-linked variants were associated with reduced volumes).

Furthermore, we added the following to the Results section on p. 19:

*“No definitive conclusions can be drawn about effect directions for each disorder due to the modest number of shared loci, yet the majority of loci for SCZ showed opposite effect directions (74%; i.e., disorder-linked variants were associated with reduced volumes), whereas the majority of loci for PD showed same effect directions (70%; i.e., disorder-linked variants were associated with increased volumes).”*

To the Discussion on p. 28-29, we added the following:

*“We do however note that a majority of the genetic loci shared between SCZ and brainstem volumes in the current study had opposite effects directions, i.e., disorder-linked variants were associated with reduced volumes. This finding is suggestive of genetic contributions to brainstem volume decreases in SCZ.”[…] “Furthermore, the majority of loci jointly associated with PD and brainstem volumes in the present study showed same effect directions, i.e., disorder-linked variants were associated with increased volumes. Although no definitive conclusions can be drawn due to the modest number of shared loci, this may suggest that brainstem volume increases in PD, if present, may be at least partly genetically mediated.”*

We added the following Supplementary Fig. 12, which illustrates effect directions for the lead SNPs shared between the brainstem volumes and the eight brain disorders. The plots indicate Z-scores for the lead SNPs across all volumes and (**a**) all disorders and (**b-i**) per disorder:

***9) I read the original publications introducing the conditional and conjunctional FDR method to investigate potential pleiotropy. As my Bayesian knowledge is limited, I find them quite difficult to understand. I would appreciate if you could add more explanation of these methods rather than the references and the short explanation on pages 64 and 65. It would be helpful if it included an explanation why this leads to an increase in power to detect more significant associations after the summary statistics “have been combined” (page 15, line 267 and pages 64 and 65).***

We thank the reviewer for the comment. The conditional FDR approach and its applications in psychiatric and neurological disorders were recently reviewed by Smeland and colleagues (*Human Genetics 2020: Discovery of shared genomic loci using the conditional false discovery rate approach*). The review of Smeland et al. includes the following formal description of the conditional and conjunctional FDR methods:

*“The ‘enrichment’ seen in conditional Q-Q plots can be directly interpreted in terms of a Bayesian true discovery rate (1 – FDR). More specifically, for a given p-value, under a simple two-group (null and non-null) model, Bayes rule gives the posterior probability of being null as:*

*FDR(p) = π_0_F_0_ (p) / F(p) – hereafter termed Equation (Eq.) [1],*

*where π_0_ is the proportion of null SNPs, F_0_ is the cumulative distribution function (cdf) of the null SNPs, and F is the cdf of all SNPs, both null and non-null. Here, we assume the SNP p-values are a priori independent and identically distributed. Under the null hypothesis, F_0_ is the cdf of the uniform distribution on the unit interval [0,1], so that Eq. [1] reduces to*

*FDR(p) = π_0_ p / F(p) – Eq [2].*

*F can be estimated by the empirical cdf q = N_p_ / Ν, where N_p_ is the number of SNPs with p-values less than or equal to p, and N is the total number of SNPs. Replacing F by q in Eq. [2], we get*

*Estimated FDR(p) = π_0_ p / q – Eq. [3],*

*which is biased upwards as an estimate of the FDR. Replacing π_0_ in Equation [3] with unity gives an estimated FDR that is further biased upward*

*q* = p/q, – Eq. [4].*

*If π_0_ is close to one, as is likely true for most GWASs, the increase in bias from Eq. [3] is minimal. The quantity 1 – p/q, is therefore biased downward, and hence a conservative estimate of the TDR. Referring to the Q-Q plots, we see that q* is equivalent to the nominal p-value divided by the empirical quantile, as defined earlier. We can thus read the FDR estimate directly off the Q-Q plot as*

*-log_10_(q*) = log_10_(q) – log_10_(p) – Eq. [5],*

*i.e. the horizontal shift of the curves in the Q-Q plots from the expected line x = y, with a larger* *shift corresponding to a smaller FDR. To estimate the conditional FDR of a given SNP, we repeat the above procedure for a subset of SNPs with p-values in the secondary GWAS equal to or lower than that observed for the given SNP. Formally, this is given by*

*FDR(p_1_|p_2_) = π_0_ (p_2_)p_1_/ F(p_1_|p_2_) – Eq. [6],*

*where p_1_ is the p-value for the first phenotype, p_2_ is the p-value for the second, F(p_1_ | p_2_) is the conditional cdf and π_0_ (p_2_) the conditional proportion of null SNPs for the first phenotype given that p-values for the second phenotype are p_2_ or smaller. The conditional FDR framework is closely related to the stratified FDR method developed by Sun et al. (2006; PMID: 16800000). Whereas they propose computing FDR separately conditional on membership in pre-defined discrete strata of p-values, here, we condition the estimated FDR on a continuous random variable, the SNP p-values with respect to a second phenotype.*

*To identify SNPs jointly associated with two phenotypes using conjunctional FDR, the conditional FDR procedure is repeated after inverting the roles of the primary and secondary phenotypes. Similar to previous conjunction tests for p-value statistics(PMID: 15808966), the conjunctional FDR estimate is defined as the maximum of both conditional FDR values, which minimizes the effect of a single phenotype driving the common association signal. Formally, the conjunctional FDR is given by*

*FDR_Phenotype1&Phenotype2_ (p_1_, p_2_) = π_0_ F_0_(p_1_, p_2_) / F(p_1_, p_2_) + π_1_ F_1_(p_1_, p_2_) / F(p_1_, p_2_) + π_2_ F_2_(p_1_, p_2_) / F(p_1_, p_2_) – Eq. [7],*

*where π_0_ is the a priori proportion of SNPs null for both phenotypes simultaneously and F_0_(p_1_, p_2_) is the joint null cdf, π_1_ is the a priori proportion of SNPs non-null for the first phenotype and null for the second with F_1_(p_1_, p_2_) the joint cdf of these SNPs, and π_2_ is the a priori proportion of SNPs non-null for the second phenotype and null for the first, with joint cdf F_2_(p_1_, p_2_). F(p_1_, p_2_) is the joint overall mixture cdf for all phenotype 1 and 2 SNPs.*

*Conditional empirical cdfs provide a model-free method to obtain conservative estimates of Eq [7]. This can be seen as follows:*

*Estimated FDR_Phenotype1&Phenotype2_ =*

*max {Estimated FDR_Phenotype1|Phenotype2,_ Estimated FDR_Phenotype2|Phenotype1_}– Eq. [8],*

*where Estimated FDR_Phenotype1|Phenotype2_ and Estimated FDR_Phenotype2|Phenotype1_ are conservative (upwardly biased) estimates of Eq. [6]. Thus, Eq (8) is a conservative estimate of max {p_1_/F(p_1_| p_2_), p_2_/F(p_2_|p_1_)} = max{p_1_F_2_(p_2_)/F(p_1_, p_2_), p_2_F_1_(p_1_)/F(p_1_, p_2_)}, with F_1_(p_1_) and F_2_(p_2_) the marginal non-null cdfs of SNPs for phenotype 1 and 2, respectively. For enriched samples, p-values will tend to be smaller than predicted from the uniform distribution, so that*

*F_1_(p_1_) ≥ p_1_ and F_2_(p_2_) ≥ p_2_. Then*

*max {p_1_F_2_(p_2_) / F(p_1_, p_2_), p_2_F_1_(p_1_) / F(p_1_, p_2_)}*

*≥ [π_0_ + π_1_ + π_2_] max{p_1_F_2_(p_2_) / F(p_1_, p_2_), p_2_F_1_(p_1_) / F(p_1_, p_2_)}*

*≥ [π_0_p_1_p_2_ + π_1_p_2_F_1_(p_1_) + π_2_p_1_F_2_(p_2_)] / F(p_1_, p_2_).*

*Under the assumption that SNPs are independent if one or both are null, reasonable for disjoint samples, this last quantity is precisely the conjunctional FDR given in Eq (7). Thus, Eq (8) is a conservative model-free estimate of the conjunctional FDR.”*

To the revised manuscript, we have added additional details to the description of the conditional FDR approach on p. 64-65 and we now point the reader to the review of Smeland et al for further details:

“A mathematical description and review of the *applications of these methods in neurological and psychiatric disorders can be found in Smeland et al.**^35^**.*”

and

*“The conditional FDR builds on an empirical Bayesian framework and uses auxiliary genetic information to re-adjust GWAS test statistics of a primary phenotype**^35^**. Conditional FDR includes separate GWAS data and leverages overlapping genetic associations to increase discovery of phenotype-associated SNPs. This approach asigns a conditional FDR value to each SNP which is defined as the probability that a SNP has no association with the first phenotype, given that the P-values for the first and second phenotypes are as small or smaller than the observed P-values^33-^**^35^**. The conditional FDR values are estimated for each nominal SNP P-value for the first phenotype after computing the stratified empirical cumulative distribution functions of the nominal P-values**^35^**.”*

***10) Is the conditional FDR method similar to GenomicSEM? If so, I would recommend that you compare results from both approaches to test for robustness of your findings.***

The conditional FDR approach is inspired by an Empirical Bayes statistical framework, as outlined above, and includes conditional QQ-plots and conjunctional FDR. The GenomicSEM approach, on the other hand, is a Structural Equation Modeling method and includes two steps. In the first step, the empirical genetic covariance matrix and its associated sampling covariance matrix is estimated. In the second step, a Structural Equation Modeling is estimated by minimizing the discrepancy between the model-implied genetic covariance matrix and the empirical covariance matrix from the first step. Thus, the conditional FDR and GenomicSEM employ different approaches and we felt that, although it would have been interesting, a comparison of the results for these two methods are outside the scope of this study.

***11) Code availability. I highly recommend the authors to make their code available on github or any other sharing platform.***

We thank the reviewer for pointing out that our previous manuscript lacked a statement stated that the codes for the conditional and conjunctional FDR methods are publicly available on <https://github.com/precimed/pleiofdr/>.

We have added the following sentence to the code availability statement in the Methods section on p. 67:

“*The codes for condFDR and conjFDR analyses are publicly available on* [*https://github.com/precimed/pleiofdr/*](https://github.com/precimed/pleiofdr/)*.*”

***12) The second phenotypic investigation feels currently somehow disconnected from the GWAS part in the UK Biobank. Are these participants not genotyped? Would it be possible to perform polygenic score analysis with the GWAS that were obtained from the UK Biobank in these samples?***

We thank the reviewer for pointing this out. We have clarified in the revised manuscript that we did not – with few exceptions – have genetic data from the clinical samples and therefore a polygenic score analysis was unfortunately not feasible. However, in the revised version of the manuscript we have improved the integration of the clinical investigations and the GWAS analyses, also in response to Reviewer #1, comment 4. We note that caution is warranted when discussing how the genetic and clinical volumetric findings could be related since the numbers of genetic loci shared between brainstem volumes and each disorder were modest. However, we have now added results and discussion that cautiously bind the two elements together. The specific changes made to the manuscript that connect the two parts are already documented in response to comment 8, yet for convenience we paste these changes below:

Results section on p. 19: *“No definitive conclusions can be drawn about effect directions for each disorder due to the modest number of shared loci, yet the majority of loci for SCZ showed opposite effect directions (74%; i.e., disorder-linked variants were associated with reduced volumes), whereas the majority of loci for PD showed same effect directions (70%; i.e., disorder-linked variants were associated with increased volumes).”*

Discussion on p. 28-29: *“We do however note that a majority of the genetic loci shared between SCZ and brainstem volumes in the current study had opposite effects directions, i.e., disorder-linked variants were associated with reduced volumes. This finding is suggestive of genetic contributions to brainstem volume decreases in SCZ.”[…] “Furthermore, the majority of loci jointly associated with PD and brainstem volumes in the present study showed same effect directions, i.e., disorder-linked variants were associated with increased volumes. Although no definitive conclusions can be drawn due to the modest number of shared loci, this may suggest that brainstem volume increases in PD, if present, may be at least partly genetically mediated.”*

***13) Figure 5. Why are the effect sizes standardised? If these are brain volumes, they are potentially measured in cm3 or any other volume measure? It would be interesting to see how big the actual difference in volume between cases and controls is adjusting for the mentioned covariates. This would allow the reader to judge actually clinical differences. Relating to this, I think it would add to the manuscript if you could present averages/scatterplots/boxplots of the brain stem volumes for healthy and affected individuals per disorder?***

We thank the reviewer for the valuable suggestion. We have added Supplementary Figs. 14 and 15 to the revised manuscript (shown on the next pages). Supplementary Fig. 14 shows the absolute volume differences in mm^3^ (residualized after accounting for the covariates) for each disorder when compared to healthy controls, whereas Supplementary Fig. 15 shows scatter plots for the volumes for each patient group and their respective controls. The main manuscript retains the report of Cohen’s d since this is the widely accepted standard in the field, yet the new supplemental figures provide all additional details needed to quantify the group differences in mm³. Thus, we added the following to the results on p. 21: “*Fig. 5 depicts the resulting case-control differences in Cohen’s d, whereas group differences in mm^3^ and scatter plots are presented in Supplementary Figs. 14-15.*”

We have added a new Supplementary Fig. 14, with volumes of brainstem structures in individuals with common brain disorders compared to healthy controls in mm^3^:

Moreover, we have added a new Supplementary Fig. 15 to the revised manuscript, with scatter plots for volumes of brainstem structures in individuals with common brain disorders and healthy controls:

***14) Looking at the quality control of the GWASs in the UK Biobank, I cannot find how relatedness in the sample has been handled. Could you please clarify this?***

We agree that we need to rule out relatedness confounds and have therefore added a dedicated analysis to the manuscript. In this analysis, we excluded all individuals in the GWAS discovery sample related up to 4^th^ degree (k=0.0625 in GCTA), leaving us with 26,329 individuals (*n* = 27,034 before accounting for relatedness). Then, we reran the GWAS for all brainstem volumes in the discovery sample. Supplementary Table 7 provides the full results that show that 1) almost all lead SNPs in the original sample are also significant at the genome-wide significance threshold in the sample with related individuals removed and 2) the genetic correlations of the summary statistics before and after excluding related individuals are all near 1. These new results support that our results cannot be explained by relatedness and we thank the reviewer for the comment. We added the following to the main text on p. 10:

“*A large majority of the lead SNPs identified in the main analyses had P < 5e-8 also in the sample without any up to 4^th^ degree related individuals (n = 26,329), supporting that the GWAS results were not driven by relatedness.*”

***15) Overall, I found the discussion difficult to follow. Especially, as it puts so much emphasis on discussing certain genes that have been associated. They may be interesting candidates; however, this is only based on closest vicinity of a lead SNP, gene-based analyses, or gene-set analysis. I would recommend cutting down on this rather speculative part of the discussion.***

We thank the reviewer for pointing this out. We have rewritten the Discussion that concerns the genetic findings and now discuss the results per brainstem subregion. We have also reduced the text relating to certain genes and hope that the discussion is now easier to follow.

**Other revisions**

We have reduced the number of references to 70.

**Reviewer comments, second version:**

Reviewer #1 (Remarks to the Author):

This reviewer was satisfied with their careful responses。


Reviewer #3 (Remarks to the Author):

The manuscript has substantially improved compared with the first submission. I congratulate you, the authors, on being able to include a replication sample. You have addressed my comments sufficiently. This paper represents interesting research findings that will further the field of imaging genetics.

However, I would like to point out for future projects: If you don't use a mixed model to account for relatedness in your sample by including a genomic relatedness matrix (GRM), one individual of two closely related individuals should be excluded based on a relatedness cut off prior to performing the GWAS. Relatedness in the sample can lead to false positives which negatively can impact secondary analysis.

Additionally, I have one last question: Has the MHC region been excluded before performing MAGMA analyses. If so, please state this in your methods section otherwise it should be performed before running MAGMA analyses.

**Author rebuttal, second version:**

**Reviewer #1:**

***This reviewer was satisfied with their careful responses.***

We thank the reviewer for all the comments and constructive suggestions, which have clearly helped us improve the manuscript.

**Reviewer #3:**

***The manuscript has substantially improved compared with the first submission. I congratulate you, the authors, on being able to include a replication sample. You have addressed my comments sufficiently. This paper represents interesting research findings that will further the field of imaging genetics.***

We thank the reviewer, and are very grateful for the positive comments and all constructive suggestions, which have clearly improved the manuscript.

***However, I would like to point out for future projects: If you don't use a mixed model to account for relatedness in your sample by including a genomic relatedness matrix (GRM), one individual of two closely related individuals should be excluded based on a relatedness cut off prior to performing the GWAS. Relatedness in the sample can lead to false positives which negatively can impact secondary analysis.***

Thank you very much for the fruitful comment, which will be useful to implement in future projects.

***Additionally, I have one last question: Has the MHC region been excluded before performing MAGMA analyses. If so, please state this in your methods section otherwise it should be performed before running MAGMA analyses.***

Thank you for pointing this out. The MHC region was excluded before performing the MAGMA analyses and we now state this in the methods section on p. 34:

*“The MHC region was excluded before running the MAGMA analyses.”*

**Reviewer comments, third version:**

**Author rebuttal, third version:**
